# Phenylimino Indolinone: A Green‐Light‐Responsive T‐Type Photoswitch Exhibiting Negative Photochromism

**DOI:** 10.1002/anie.202111748

**Published:** 2021-10-22

**Authors:** Stefano Crespi, Nadja A. Simeth, Mariangela Di Donato, Sandra Doria, Charlotte N. Stindt, Michiel F. Hilbers, Ferdinand L. Kiss, Ryojun Toyoda, Sammo Wesseling, Wybren Jan Buma, Ben L. Feringa, Wiktor Szymański

**Affiliations:** ^1^ Stratingh Institute for Chemistry University of Groningen Nijenborgh 4 9747 AG Groningen The Netherlands; ^2^ ICCOM-CNR via Madonna del Piano 10 50019 Sesto Fiorentino Italy; ^3^ European Laboratory for Non Linear Spectroscopy (LENS) via N. Carrara 1 50019 Sesto Fiorentino Italy; ^4^ Van't Hoff Institute for Molecular Sciences University of Amsterdam Science Park 904 1098 XH Amsterdam The Netherlands; ^5^ Department Chemie Ludwig-Maximilians-Universität München 81377 München Germany; ^6^ Institute for Molecules and Materials FELIX Laboratory Radboud University Toernooiveld 7c 6525 ED Nijmegen The Netherlands; ^7^ Department of Radiology, Medical Imaging Center University Medical Center Groningen, University of Groningen Hanzeplein 1 9713 GZ Groningen The Netherlands

**Keywords:** computational chemistry, isomerization, molecular dynamics, photochromism, time-resolved spectroscopy

## Abstract

Imines are photoaddressable motifs useful in the development of new generations of molecular switches, but their operation with low‐energy photons and control over isomer stability remain challenging. Based on a computational design, we developed phenylimino indolinone (PIO), a green‐light‐addressable T‐type photoswitch showing negative photochromism. The isomerization behavior of this photoactuator of the iminothioindoxyl (ITI) class was studied using time‐resolved spectroscopies on time scales from femtoseconds to the steady state and by quantum‐chemical analyses. The understanding of the isomerization properties and substituent effects governing these photoswitches opens new avenues for the development of novel T‐type visible‐light‐addressable photoactuators based on C=N bonds.

## Graphical abstract

Visible‐light‐responsive T‐type photoswitches with negative photochromism are rare and interesting candidates for a wide range of applications. Here, we describe the computer‐assisted rational design of phenylimino indolinone (PIO) and its characterization in the ground and excited state using the full toolbox of physical organic chemistry.

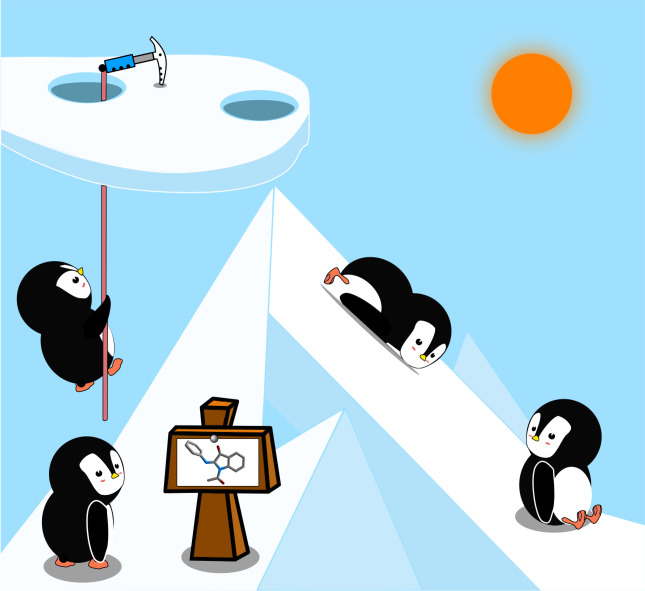

Photoswitches are the cornerstone of molecular machinery.[^1^, ^2^, ^3^, ^4^, ^5^] Upon irradiation, these *photoactuators* can populate a metastable state from which they can be reverted to the initial isomer using another photochemical (P‐type photoswitch) or a thermal (T‐type photoswitch) stimulus.^[6]^ Accessing the different physical and chemical properties of the two isomers by light irradiation allows to control the photoswitch both from a spatial and a temporal point of view, thus opening countless possibilities for applications among others in optics, smart materials, molecular logic or photopharmacology.[^1^, ^7^, ^8^, ^9^, ^10^, ^11^]

In the development of new photoactuators, the photochemical *E*/*Z* isomerization of C=N bonds is an alternative to the ones offered by the well‐established C=C and N=N motifs.[^12^, ^13^, ^14^, ^15^, ^16^, ^17^, ^18^, ^19^] Similar to azobenzene,^[20]^ the presence of the nitrogen lone‐pair in the C=N systems dictates the photochemistry and thermal isomerization of imines.[^19^, ^21^] The interconversion between the isomers is assumed to occur following two different mechanisms: an excited state *rotation* of the nitrogen substituent about the C=N bond in which the double bond is formally broken, or a ground state in‐plane *inversion* without change in the bond order.^[22]^ Taming this complex scenario allowed the realization of the first two‐ and four‐stroke molecular motors by the group of Lehn[^14^, ^15^] and bistable switches based on hydrazones.^[16]^

Recently, we developed the new class of iminothioindoxyl (**ITI**) photoswitches based on a C=N chromophore (see Figure 1 A).^[23]^ This new photoactuator exhibits a remarkable band separation of over 100 nm between its two photoisomers, a prerogative to address each isomeric form selectively. **ITI** is structurally related to other indigoid photoswitches and readily absorbs light in the visible range. Irradiation with blue light excites the thermally stable *Z* isomer to its bright S_2_ state. From there, it relaxes and forms the metastable *E* isomer with quantum yields Φ around 5 % and showing positive photochromism.^[6]^ The *E* form can be isomerized back to the stable form using orange light, or by thermal relaxation with recovery times in the ms time range making it a T‐type switch.^[23]^ Triggering the photochemical *E*→*Z* isomerization, which in the **ITI** system represents the back‐switching direction, employs electromagnetic radiation of low energy and is consequently more attractive for practical applications.

**Figure 1:**
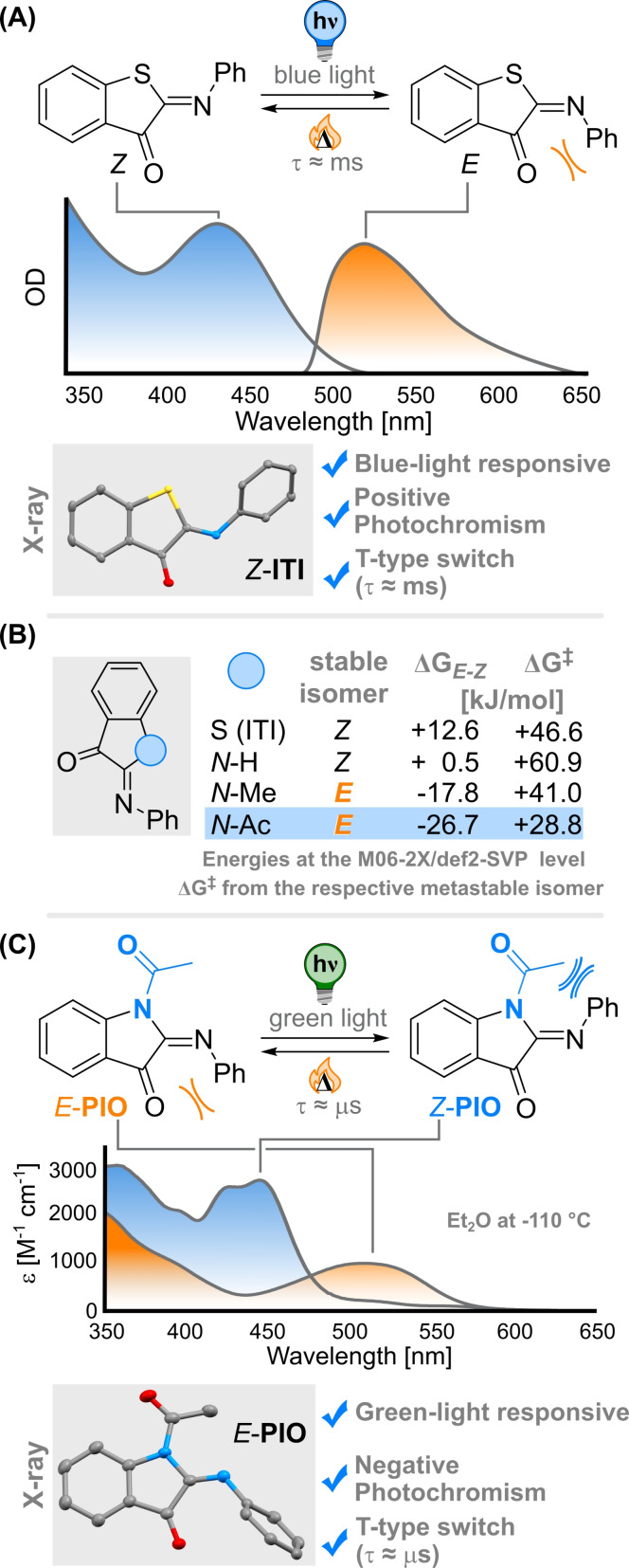
Iminothioindoxyl (**ITI**) and phenylimino indolinone switches (**PIO**). (A) Properties of the parent **ITI** photoswitch.^[23]^ The X‐ray structure of *Z*‐**ITI** (50 % probability ellipsoids, H atoms omitted for clarity) is reported. (B) Computational screening of the principal thermal characteristics of different substituents on the original **ITI** core. (C) Main features of **PIO**, the experimental UV/Vis spectra of *E‐* and *Z*‐**PIO** (the latter was extrapolated from the PSS mixture) in Et_2_O, together with the X‐ray structure of *E*‐**PIO** (50 % probability ellipsoids, H atoms omitted for clarity).

Hence, we were intrigued by the possibility of inverting the stability of the isomers in **ITI** while retaining their photophysical properties. Such a novel structure, for which the *E* isomer would be the stable one, would then respond to low‐energy light irradiation and, more importantly, be characterized by *negative photochromism*.[^6^, ^24^]

In this respect, the light‐induced bleaching of the sample would avoid inner filter effects, a factor that limits the efficiency of photoisomerization for strongly colored photoswitches, especially at high concentrations.^[24]^

The reason behind the thermodynamical stability of *Z*‐**ITI**, as compared with *E*‐**ITI** (by 12.6 kJ mol^−1^), is the limited repulsion between the phenyl group and the sulfur atom of the benzothiophene‐3‐one moiety in *Z*‐**ITI**, whereas *E*‐**ITI** is destabilized by steric clashes between the phenyl ring and the carbonyl group (see Figure 1 B).^[23]^ Using computational modeling, we realized that replacing the sulfur with bulkier N‐R moieties destabilizes the *Z*‐form and inverts the relative population of the two photoisomers, with *N*‐Ac affording a Δ*G* of 26.7 kJ mol^−1^ favoring the *E*‐form (Figure 1 B, see Supporting Information for computational analysis). Similar effects have been observed for hemiindigo P‐type photoswitches upon alkylation of the nitrogen atom.^[25]^ In addition, the introduction of bulkier substituents reduces the predicted thermal isomerization barrier of the metastable form (see Figure 1 B) and increases its thermal lability. T‐type photoswitches characterized by a fast thermal recovery^[26]^ are applied as photoactuators in optical lenses,^[27]^ super‐resolution microscopy,^[28]^ real‐time holography,^[29]^ photomechanical systems,[^30^, ^31^] and are designed as photopharmacological tools.^[32]^

Taking the challenge to reverse the stability of the isomers, i.e., from positive to negative photochromism while maintaining a large band separation in the visible, we designed phenylimino indolinone (**PIO**, see Figure 1 C), expected to be more sterically hindered than **ITI**, by the synthetically facile introduction of an *N*‐acetyl moiety into its core part. The synthesis of **PIO** consisted of a three‐step procedure starting from 1H‐indol‐3‐yl acetate with an overall 80 % yield (see SI for the detailed synthetic procedure and characterization). The synthesis afforded a purple solid absorbing at 508 nm in cyclohexane (see Table 1); characteristics that closely matched the computed TD‐DFT spectrum of the *E*‐isomer (see SI). The predicted difference in the absolute configuration of the stable isomers of **PIO** and **ITI** was confirmed by X‐ray analysis (see Figure 1 A and 1C). *E*‐**PIO** shows a very limited solvatochromism, in line with what has been observed in **ITI**,^[23]^ and relatively low *ϵ* values (see Table 1).

**Table 1:** Main photophysical, photochemical and thermal parameters for **PIO** in various solvents.

Solvent	Φ_*EZ*_^[a]^ [%]	*E λ*_max_ [nm]	*ϵ*/1000 [M^−1^ cm^−1^]	*Z λ*_max_ [nm]^[a,b]^	τ_*ZE*_ [μs]^[a]^
Cyclohexane	9.0	508	0.93	426	27
Toluene	6.7	510	0.93	436	77
DCM	6.9	509	1.0	437	74
MeOH	11.6	498	0.62	431	111
MeCN	5.6	500	0.78	435	76
DMSO	4.8	496	0.91	435	67

[a] Measured with ns transient absorption spectroscopy at 20 °C. [b] Data inferred from the *λ*_max_ of the positive transient signal recorded with ns transient absorption spectroscopy after excitation at *λ*=510 nm.

Indeed, the S_0_→S_1_ transition is only partially allowed (f=0.02, predicted in vacuo at the TD‐M062X/6–311+G(2d,p) level of theory).^[23]^ This transition has a mixed n→π* and π→π* character (see SI). On the other hand, the simulated UV/Vis spectrum of the metastable *Z*‐isomer predicted the expected negative photochromism, when compared with the *E*‐form (see Figure 1 C).

Encouraged by these results, we studied the excited‐state behavior of **PIO**. We recorded femtosecond transient absorption (TA) spectra of *E*‐**PIO** in different organic solvents upon excitation at 500 nm (*cf*. Figure 2 A for MeOH and toluene and Table S14). In toluene, directly after excitation we observed an intense and broad excited state absorption band with *λ*_max_ at ca. 524 nm (see Figure 2 A, inset). The intensity of this signal is significantly reduced in about 1.4 ps (τ_1_). In analogy to **ITI**, we interpret this initial decay as the excited‐state evolution toward the S_1_→S_0_ conical intersection (CInt, see Figure 2 B). This transient evolves, forming two positive signals peaked at 425 and 550 nm. The latter band decays completely in 4.8 ps (τ_2_). These two positive bands are attributed to absorption from the hot ground state of *Z*‐ and *E*‐**PIO**, respectively. The final spectral component has a differential line shape with a negative contribution in the 470–550 nm region where ground state absorption of the initial isomer is observed, and a positive product band at shorter wavelengths (ca. 70–80 nm blue‐shifted, see Table 1). The persistence of these long‐living contributions confirms the photoisomerization and the predicted negative photochromism of **PIO**.

**Figure 2:**
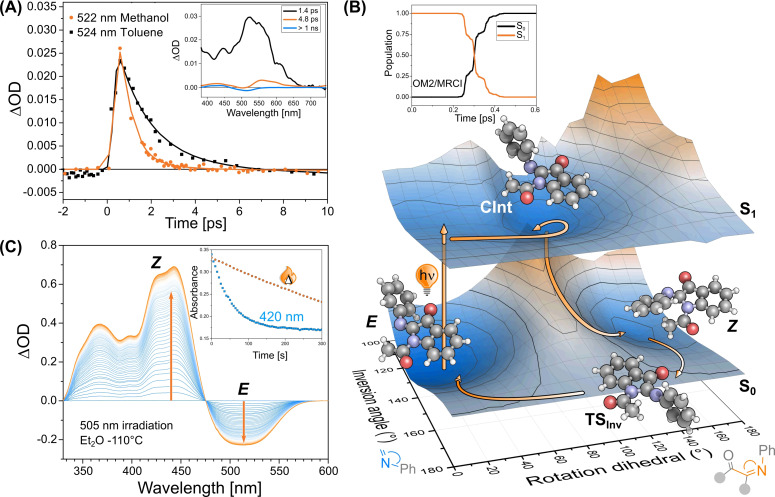
Spectroscopic and computational studies on the isomerization of **PIO**. (A) Comparison of the short timescale kinetic traces of the excited‐state absorption band of **PIO** dissolved in toluene and methanol, upon excitation at 500 nm. Inset: Evolution‐associated difference spectrum (EADS) obtained from global analysis of transient absorption data recorded for **PIO** in toluene upon excitation at 500 nm. (B) Representation of the full isomerization cycle in **PIO**. Surfaces obtained as a SF‐BH&HLYP/cc‐pVDZ single point calculation on the geometry optimized at the TD‐ωB97X‐D/MIDI! (S_1_) or GFN2‐xTB (S_0_) level of theory. Inset: Population of S_0_ and S_1_ states as a function of time obtained from OM2/MRCI NAMD calculations. (C) Differential spectra of *E*‐**PIO** in Et_2_O (3×10^−4^ M) upon irradiation with a 505 nm LED. PSS was reached after 22 min. Inset: kinetics observed at 443 nm when irradiating *Z*‐**PIO** with 420 nm LED vs. thermal isomerization.

The excited‐state decay is observed to be faster in polar solvents than in non‐polar ones (see Figure 2 A and SI). The Φ_*EZ*_ values are slightly higher than the ones found for **ITI** (see Table 1),^[23]^ showing a tendency of the excited system to form the *Z*‐isomer with 5–12 % efficiency. We qualitatively reproduced this trend by mapping the ground and excited state with spin‐flip DFT (SF‐DFT) and simulating the first picoseconds after excitation using nonadiabatic molecular dynamics simulations (NAMD) at the OM2/MRCI level of theory, starting from 320 trajectories of *E*‐**PIO** in a canonical ensemble (see Figure 2 B and SI).[^33^, ^34^] After excitation, **PIO** undergoes a barrierless rotation of the C=N bond^[19]^ that drives the system toward the region of the CInt. When reaching a perpendicular arrangement of the imine bond, the molecule hops to the ground state with a predicted lifetime τ_S1_ of 250 fs and quantum yield of 24 %, which correctly mirrors the preference of the system toward the photochemical recovery of the *E*‐form.

Low‐temperature NMR confirmed the predicted geometrical features of *Z*‐**PIO** (see SI). Upon irradiation at *λ*=505 nm, the signal attributed to the acetyl CH_3_ shifted upfield about 1.2 ppm, due to the shielding exerted by the phenyl ring in the *Z* form (see SI). The thermal recovery of *Z*‐**PIO** was followed by ns transient absorption spectroscopy. Table 1 shows that the thermal isomerization process is influenced only to a minor extent by the solvent with recovery times τ_*ZE*_ ranging from 27 to 111 μs.

Irradiating *E*‐**PIO** with a 505 nm LED at −110 °C in Et_2_O (Figure 2 C) enabled to reach a photostationary state (PSS) with a distribution of 80:20 of the *Z* and *E* isomers as determined from low‐temperature NMR (see SI), a value higher than the one reported for **ITI**, as expected due to the negative photochromism of **PIO**. After achieving the *Z*‐**PIO**‐enriched PSS under irradiation with 505 nm light in the UV/Vis at −110 °C, we were able to switch it back to the *E*‐isomer by irradiating the band associated with *Z*‐**PIO** with a 420 nm LED (Figure 2 C, inset and SI). Thus, we could also prove that reversibility of the photochemical reaction can be achieved using light, by increasing the thermal stability of **PIO** at low temperatures for enhanced temporal control by P‐type isomerization. In addition, we followed the thermal decay of the same band with *λ*_max_ at 443 nm at increasing temperatures, and consequently determined the Δ*G* ^≠^ for the thermal isomerization to be 48.3 kJ mol^−1^ (see SI). Using these values, a lifetime of 73 μs is extrapolated for 20 °C, which nicely agrees with the data recorded with ns TA spectroscopy at room temperature in other solvents.

The electronic nature of the substituents on the phenyl moiety has a remarkable effect on the properties of **PIO**. While the UV/Vis absorption is bathochromically shifted by the presence of electron‐donating groups (see Figure 3 A), the thermal *Z*→*E* transformation can be tuned from *τ*=1.5 μs when NO_2_ is used to 800 μs for MeO (both values in MeOH, see Figure 3 B and SI). Probing the kinetics in both MeOH and MeCN for seven differently phenyl‐substituted **PIO**s, we obtained the two Hammett plots depicted in Figure 3 B. In both solvents, the positive ρ value (2.11 for MeCN and 2.46 for MeOH) and the relatively high linearity of the plots reflect the stabilization effect of electron‐withdrawing groups on the partial negative charge on the imine nitrogen while reaching the inversion transition state geometry (TSInv, Figure 3 B), as correctly predicted by DFT when the *Z→E* thermal isomerization proceeds via nitrogen inversion. For **PIO**, the Natural charge at the imine nitrogen obtained from NBO analysis was calculated to slightly increase going from the *Z* form to the transition state (see Table S16). This tendency is more marked when electron‐donating substituents are present, while it shows opposite sign in the case of electron‐withdrawing ones (see δ^−^ and δδ^−^ values reported in Table S16). Indeed, the substituents affect the predicted activation energies following the same experimental trend, with a computed positive *ρ*=1.66 in the simulated Hammett plot (see Figure 3 B).

**Figure 3:**
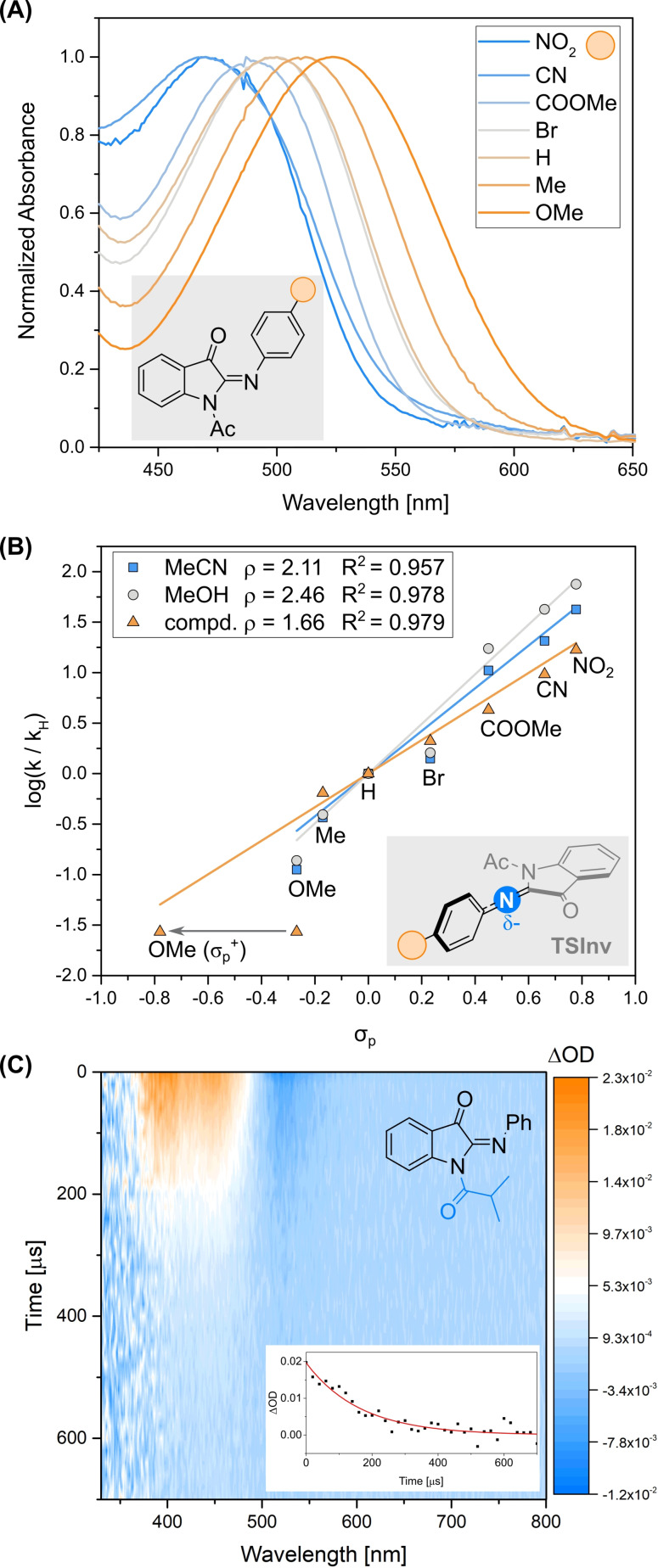
Properties of substituted **PIO** derivatives. (A) Normalized UV/Vis spectra of substituted **PIO** derivatives. (B) Hammett plots for the thermal back‐isomerisation step kinetics, obtained using the σ_p_ values for the phenyl‐substituted **PIO** derivatives in MeCN and MeOH (measured at 20 °C) compared to the computed values obtained at the M06‐2X/def2‐SVP level of theory in vacuo. For the latter, a better correlation was obtained employing the σ_p_^+^ value for OMe. (C) Heatmap of the ns transient absorption spectra of **iBu‐PIO**. In the inset, fit of the decay of the absorption maximum (395 nm) of the transient signal (red line) as obtained from a global analysis of the transient spectra.

To further probe the generality of this new switch towards functionalization, we focused on the amidic nitrogen and studied the synthetically easily accessible *iso*‐butyryl **PIO** derivative (**iBu‐PIO**, Figure 3 C). Modifications of the substituent α to the amidyl CO has only a limited effect compared to the acetyl group, both in terms of quantum yields (3–6 %) and lifetimes (in MeCN, *τ*=164 μs, see Figure 3 C and Table S15). The latter are similar, yet slightly longer compared to the unsubstituted **PIO** (Figure 3 B), an effect attributable to an increased destabilization of the TSInv, which could be correctly modelled by DFT (see Table S16), validating our predictive method for this new class of photoswitches.

In conclusion, using an extensive spectroscopic and computational characterization, we demonstrated that negative photochromism in phenyl iminoindigoid photoswitches can be achieved by increasing the steric bulk of the bicyclic core. Simultaneously, we could retain the pronounced band separation of ca. 80 nm between the two photoisomers typical of the class. In this way, we designed and synthesized **PIO**, a novel T‐type photoswitch that is responsive to green light.

The photochromism of **PIO** and its derivatives constitutes one of the few cases in which the simple molecular engineering allowed for the inversion of the stability of the isomers in the photoswitch.^[25]^ A similar inversion was achieved for example, for azobenzenes, but requires the covalent linking of the two aromatic rings to enforce the higher stability of the usually metastable cis isomer.^[35]^ Visible‐light responsive T‐type photoswitches with negative photochromism reported here are interesting candidates for designing new molecular machines and photoresponsive molecular systems.[^7^, ^27^, ^28^, ^29^, ^30^, ^31^, ^32^]

## Conflict of interest

The authors declare no conflict of interest.

## Supporting information

As a service to our authors and readers, this journal provides supporting information supplied by the authors. Such materials are peer reviewed and may be re‐organized for online delivery, but are not copy‐edited or typeset. Technical support issues arising from supporting information (other than missing files) should be addressed to the authors.

Supporting Information

Supporting Information

Supporting Information

Supporting Information

Supporting Information

Supporting Information
