# Supplementary material for: Phenylimino Indolinone: A Green‐Light‐Responsive T‐Type Photoswitch Exhibiting Negative Photochromism
Source: Angew Chem Int Ed Engl. 2021 Oct 22;60(48):25290–5. doi: 10.1002/anie.202111748 (PMC9298291; doi:10.1002/anie.202111748)
Supplement: Supplementary file 2 — Supporting Information [file ANIE-60-25290-s002.pdf]

Angewandte Chemie
Eine Zeitschrift der Gesellschaft Deutscher Chemiker

Supporting Information

Phenylimino Indolinone: A Green-Light-Responsive T-Type Photoswitch Exhibiting Negative Photochromism

Stefano Crespi[+], Nadja A. Simeth[+], Mariangela Di Donato, Sandra Doria, Charlotte N. Stindt, Michiel F. Hilbers, Ferdinand L. Kiss, Ryojun Toyoda, Sammo Wesseling, Wybren Jan Buma, Ben L. Feringa,* and Wiktor Szymański**

anie_202111748_sm_miscellaneous_information.pdf
anie_202111748_sm_ITI_xray.mp4
anie_202111748_sm_NAMD_EZ.mp4
anie_202111748_sm_PIO_formation.mp4
anie_202111748_sm_PIO_xray.mp4
anie_202111748_sm_SI.zip

Table of Contents

Experimental Procedures **3**

General Methods *3*

X-Ray analysis *3*

Femtosecond Transient Absorption Spectroscopy *3*

Nanosecond Transient Absorption Spectroscopy *4*

Computational methods *4*

Results and Discussion **5**

Synthesis and characterization *5*

1-Acetyl-indol-3-yl acetate (**2**) 5

1-Acetylindolin-3-one (**3**) 6

(E)-1-Acetyl-2-(phenylimino)indolin-3-one (**PIO**) 6

1-Bromo-4-nitrosobenzene (**8**) 8

4-Nitrosobenzonitrile (**9**) 8

(E)-1-acetyl-2-(p-tolylimino)indolin-3-one (**PIO-Me**) 9

Methyl (E)-4-((1-acetyl-3-oxoindolin-2-ylidene)amino)benzoate (**PIO-COOMe**) 11

(E)-1-acetyl-2-((4-nitrophenyl)imino)indolin-3-one (**PIO-NO_2**) 12

(E)-1-acetyl-2-((4-bromophenyl)imino)indolin-3-one (**PIO-Br**) 14

(E)-4-((1-acetyl-3-oxoindolin-2-ylidene)amino)benzonitrile (**PIO-CN**) 15

(E)-1-acetyl-2-((4-methoxyphenyl)imino)indolin-3-one (**PIO-OMe**) 17

1-Isobutyrylindolin-3-one (**11**) 18

(E)-1-isobutyryl-2-(phenylimino)indolin-3-one (**iBu-PIO**) 20

UV-Vis characterization 22

X-Ray structural data *24*

E-**PIO** 24

Z-**ITI** 27

Femtosecond Transient Absorption Spectroscopy *30*

Nanosecond Transient Absorption Spectroscopy *32*

Low-temperature NMR, UV-Vis and Activation Parameter Analysis *42*

Low temperature NMR 42

Activation parameters 45

Z-to-E isomerisation by irradiation with 455 and 420 nm light 46

Computational analysis *47*

References **53**

Author Contributions **53**

Experimental Procedures

General Methods

Reagents were purchased from Sigma-Aldrich, Alfa Aesar, TCI, Boom, and Combi-Blocks, and used without further purification. Dry solvents were collected from a Pure Solve MD5 solvent dispenser from Demaco or by drying them for 48 h over 3 Å molecular sieves. All reactions were performed under magnetic stirring. For dry or oxygen-free reactions, standard Schlenk techniques were used, employing nitrogen as the inert gas. The crimp top vials were purchased from Wicom.
Flash chromatography was performed on silica gel (Screening devices B.V.) with a particle size of 40−64 μM and pore size of 60 Å or on Buchi EcoFlex silica columns (4 -25 g, 60 Å) using a Buchi Reveleris X2 system. TLC analysis was conducted on TLC aluminum foils with a silica gel matrix (Supelco, silica gel 60) with detection by UV (254 nm or 366 nm) or suitable staining.
^{1}H NMR and ^{13}C NMR were recorded in DMSO-d_6, MeOD-d_3, chloroform-*d*, or diethylether-*d10* with the chemical shift (δ) relative to the solvent peak on an Agilent Technologies 400-MR (400/54 Premium Shielded) spectrometer (400 MHz) or on a Bruker 600 MHz NMR spectrometer. All spectra were measured at room temperature (22–24 °C). Characterization of the signals: s = singlet, d = doublet, t = triplet, q = quartet, m = multiplet, *b*s = broad singlet, dd = doublet of doublet, dt = doublet of triplet. Integration is directly proportional to the number of protons. Characterization of the ^{13}C-NMR signals: (+) for CH_3 or CH, (-) for CH_2 and (q) for quaternary C-atoms. This assignment resulted from APT or DEPT135.
Low-temperature NMR was recorded on a Varian Unity Plus 500 MHz NMR at –105 °C and irradiation was performed in-situ with a 505 nm LED, via a fiber optic cable from Thorlabs (FT400UMT) modified to fit into the NMR tube itself, as described elsewhere.[1]
High Resolution Mass Spectra were recorded on a Thermofisher LTQ Orbitrap XL with eluent MeOH (0.1% TFA) and flow rate of 0.15 mL min-1 in positive (ACPI/ESI) mode. UPLC-MS measurements were performed using a Thermo Fisher Scientific Vanquish UPLC System on a C18 column with a LCQ Fleet ion trap mass spectrometer.
Infrared (IR) spectra were recorded on a PerkinElmer UATR (Single Reflection Diamond) Spectrum Two device (4000−700 cm^{-1}; resolution 4 cm^{-1}).
Melting point ranges were determined on a Stuart analogue capillary melting point SMP11 apparatus.
Electronic absorption spectra at room temperature were measured using an Agilent 8453, while the low-temperature measurements were carried out on an Agilent 8454 equipped with an Unisoku USP-203 cryostat. Raw data were processed using Agilent UV-Vis ChemStation B.02.01 SP1, Spectragryph 1.2, Glotaran 1.5.1[2] and Origin 2018.

X-Ray analysis

Z-**ITI** was crystallized from slow diffusion of a mixture of chloroform and heptane. *E*-**PIO** was recrystallized and single crystals grown in Et_2O. A single-crystal of either *E*-**PIO** or *Z*-**ITI** was mounted on a cryoloop and placed in the nitrogen stream (100 K) of a Bruker-AXS D8 Venture diffractometer. Data collection and processing was carried out using the Bruker APEX3 software suite.[3] A multi-scan absorption correction was applied, based on the intensities of symmetry-related reflections measured at different angular settings (*SADABS*).[4] The structure was solved using *SHELXT*[5] and refinement was performed using *SHELXL*.[6] The hydrogen atoms were generated by geometrical considerations, constrained by idealized geometries and allowed to ride on their carrier atoms with an isotropic displacement parameter related to the equivalent displacement parameter of their carrier atoms. No A- or B-level alerts were raised by CheckCIF for the fully refined structure.
The cif files and videos of the structures are provided as additional files.

Femtosecond Transient Absorption Spectroscopy

The apparatus used for the transient absorption spectroscopy (TAS) measurements has been described in detail before.[7] Briefly, 80 fs pulses centred at 810 nm were produced by an integrated home-made Ti:sapphire oscillator coupled with a regenerative amplifier system (Amplitude Pulsar). The excitation wavelength was set at 500 or 520 nm and excitation power was set at 30-50 nJ for all measurements. Visible pulses were generated by pumping a home-made non-collinear optical parametric amplifier (NOPA) with a portion of the fundamental 810 nm radiation. The pump beam polarization has been set to magic angle with respect to the probe beam by rotating a λ\2 plate, to exclude rotational contributions. The white light probe pulse was generated by focusing a small portion of the fundamental laser radiation on a 3 mm thick CaF_2 window. A portion of the generated white light was sent to the sample through a different path and used as a reference signal. After passing through the sample the white light probe and reference pulses were both directed to a flat field monochromator coupled to a home-made CCD detector. Transient signals were acquired in a time interval spanning up to 500 ps. The sample was contained in a 2 mm quartz cuvette, mounted on a movable holder in order to minimize photodegradation. Measurements were performed at room temperature. Concentrations were adjusted to an absorbance of 0.9 – 1.0 OD (for the respective optical path) at the absorption maximum which amounted to about 0.3 – 0.5 OD at excitation wavelength. Before and after the measurements, the integrity of the sample was checked on a PerkinElmer LAMBDA 950 spectrophotometer.

Nanosecond Transient Absorption Spectroscopy

Nanosecond transient absorption spectra were recorded with an in-house assembled setup. A different excitation wavelength (480 - 530 nm) was used depending on the absorption properties of the sample. The excitation wavelength was generated using a tunable Nd:YAG-laser system (NT342B, Ekspla) comprising the pump laser (NL300) with harmonics generators (SHG, THG) producing 355 nm to pump an optical parametric oscillator (OPO) with SHG connected in a single device. The laser system was operated at a repetition rate of 10 Hz with a pulse length of 5 ns. The probe light running at 20 Hz was generated by a high-stability short arc xenon flash lamp (FX-1160, Excelitas Technologies) using a modified PS302 controller (EG&G). Using a 50/50 beam splitter, the probe light was split equally into a signal beam and a reference beam and focused (bi-convex lens 75mm) on the entrance slit of a spectrograph (SpectraPro-150, Princeton Instruments) with a grating of 150 ln/mm, blaze at 500 nm. The probe beam (A = 1 mm^2) was passed through the sample cell and orthogonally overlapped with the excitation beam on a 1 mm × 1 cm area. The excitation energy was recorded by measuring the excitation power at the back of an empty sample holder. In order to correct for fluctuations in the flash lamp spectral intensity, the reference was used to normalize the signal. Both beams were recorded simultaneously using a gated intensified CCD camera (PI-MAX3, Princeton Instruments) which has an adjustable gate of minimal 2.9 ns, normally a gate of 20 ns and software binning is used to improve the dynamic range and signal to noise ratio. Two delay generators (DG535 and DG645, Stanford Research Systems, Inc.) were used to trigger the excitation and to change the delay of the flash lamp together with the gate of the camera during the experiment. The setup was controlled by an in-house written Labview program.

Computational methods

A series of different methods were employed to optimize the structures of the ground state minima and transition states of the differently substituted compounds of the ITI family presented in Fig. 1 of the main text. All the structures were pre-screened using the CREST driver in the xTB 6.3.2 software,[8] using the GFN2-xTB semiempirical level of theory.[9] In this way, the most stable conformers for each structure were picked via the default series of metadynamics and dynamics runs implemented in the driver. The most stable conformers and the transition states connecting them were optimized at the M06-2X/def2-SVP level.[10,11] The nature of the stationary point found was confirmed computing the hessian matrix and inspecting the number of imaginary frequencies found (0 for the minima, 1 for the transition states). Electronic spectra were simulated on the stable and metastable configurations previously optimized, predicting the 30 lowest singlet transitions at the M06-2X/6-311+G(2d,p). The feasibility of the functional to correctly simulate the state ordering in the ITI photoswitches was previously benchmarked.[12] All DFT optimizations were conducted with the Gaussian 16, Rev B.01 software package.[13]

Semiempirical calculations and nonadiabatic molecular dynamics simulations were conducted at the OM2/MRCI level of theory,[14,15] as implemented in the MNDO program.[16] The active space in the MRCI calculations included twelve electrons in eleven orbitals (12,11). All the orbitals were of π character or a mixed n and π character (see Figure S62).

For the MRCI treatment, three configuration state functions were chosen as references, namely the leading configuration with two singly occupied orbitals (which defines the ROHF formalism) and the two closed-shell configurations derived therefrom (i.e., the singlet configurations with doubly occupied HOMO or LUMO of the closed-shell ground state). The MRCI wavefunction was built by allowing all single and double excitations from these three references (CISD).

All the optimizations were conducted including the lowest three singlet states. The geometry optimization of minima employed the BFGS update. The nature of the minima and transition states was checked inspecting the number of imaginary frequencies after the calculation of the force constants at the optimized geometry (i.e., 0 for minima, 1 for transition states). Transition states were located using the eigenvector following algorithm, while the default optimizer was employed for the minima. The conical intersection was optimized using a modified version[17] of the Lagrange-Newton algorithm proposed by Manaa and Yarkony.[18]

Nonadiabatic molecular dynamics (NAMD) simulations on the E geometry were performed using the Tully surface-hopping (TSH) method as implemented in the MNDO program, with an analytical evaluation of the nonadiabatic coupling vectors.[19] All simulations were in a canonical ensemble maintained by a Nosé-Hoover thermostat (T=300 K). The sampling of the initial structures and relative initial velocities was obtained via a preliminary ground-state Born-Oppenheimer dynamics run of 20 ps with 0.5 fs time step. The initial structures for the surface-hopping dynamics were chosen from these ground-state trajectories using the filtering procedure implemented in the MNDO program, combining the active space mapping with a threshold reduced to 70%, and the transition probability to the target state, based on a stochastic algorithm. The three lowest singlet states were included in the NAMD runs. The initial state chosen for starting the dynamics was S1. 320 runs of 600 fs with time step of 0.1 fs were evaluated. The default empirical decoherence correction of 0.1 Hartree was used.[20]

The S_0-S_1 conical intersection was also optimized at the SF-BH&HLYP/cc-pVDZ level over three roots,[21] using the penalty function algorithm as implemented in GAMESS-US (Ver: 30 SEP 2020 (R2)).[22] The excited state landscape was obtained as a constrained optimization of the inversion angle and rotation dihedral of the C=N bonds using the TDA(3 states)-ωB97X-D/MIDI! level of theory[23,24] as implemented in the Gaussian 16, Rev B.01 software. The ground state optimizations using the same constraints were done using the GFN2-xTB level from the xTB 6.3.2 package. All these geometries were then corrected at the SF-BH&HLYP/cc-pVDZ level over five states, using GAMESS-US (Ver: 30 SEP 2020 (R2)).

Natural charges were computed using NBO 7.0.9 interfaced with Gaussian 16.[25]

All xyz coordinates for all the compounds considered are provided as a separate additional file.

A video of a productive E-Z NAMD trajectory computed at the OM2/MRCI level is provided as a separate file.

Results and Discussion

Synthesis and characterization

Parent Compounds

Z-**ITI** was synthesized according to a literature procedure.[12]
E-**PIO** was synthesized following the synthetic scheme presented in Scheme S1.

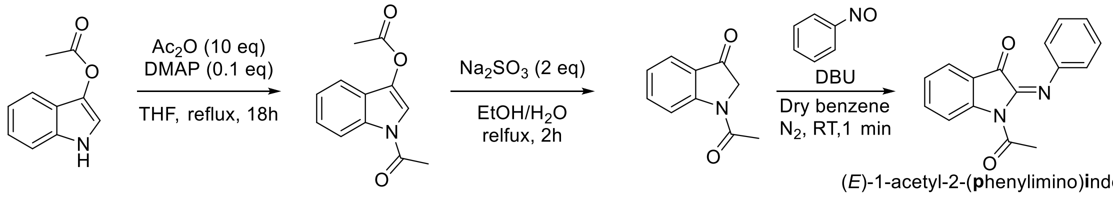

1 **2** (93%) **3** (86%) (*E*)-1-acetyl-2-(**p**henylimino)**i**ndolin-3-**o**ne **PIO** (*quant.*)

Scheme S1. Overview of the synthetic route towards *E*-**PIO**.

1-Acetyl-indol-3-yl acetate (**2**)

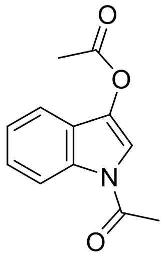

A crimp top vial was charged with 1H-indol-3-yl acetate (2.00 g, 11.4 mmol, 1.00 eq.) and DMAP (0.20 g, 1.6 mmol, 0.14 eq.) dissolved in THF (60 mL). Acetic anhydride (11.7 g, 10.8 mL, 0.11 mol, 10.0 eq.) was added, the vial was sealed, and the mixture was heated to 85 °C for 16 h. The mixture was cooled to ambient temperature, diluted with EtOAc (50 mL), and poured onto NH_4Cl (aq, sat, 100 mL). The phases were separated, and the organic phase was washed with brine (2 × 60 mL). The organic layer was dried over Na_2SO_4 and the volatiles were evaporated. The light brown solid was used in the next step without further purification (2.30 g, 10.6 mmol, 93%). An analytical sample was purified over a silica plug using EtOAc as eluent.
^{1}H NMR (400 MHz, DMSO-*d*$_6$) δ = 8.36 (dt, *J* = 8.3, 0.9 Hz, 1H), 7.89 (s, 1H), 7.52 (ddd, *J* = 7.8, 1.3, 0.7 Hz, 1H), 7.39 (ddd, *J* = 8.4, 7.2, 1.4 Hz, 1H), 7.31 (ddd, *J* = 8.1, 7.3, 1.1 Hz, 1H), 2.62 (s, 3H), 2.39 (s, 3H).
R$_f$ (SiO2; EtOAc : Hexanes, 1:2, v/v): 0.61. The analytical data are in agreement with published data.[26]

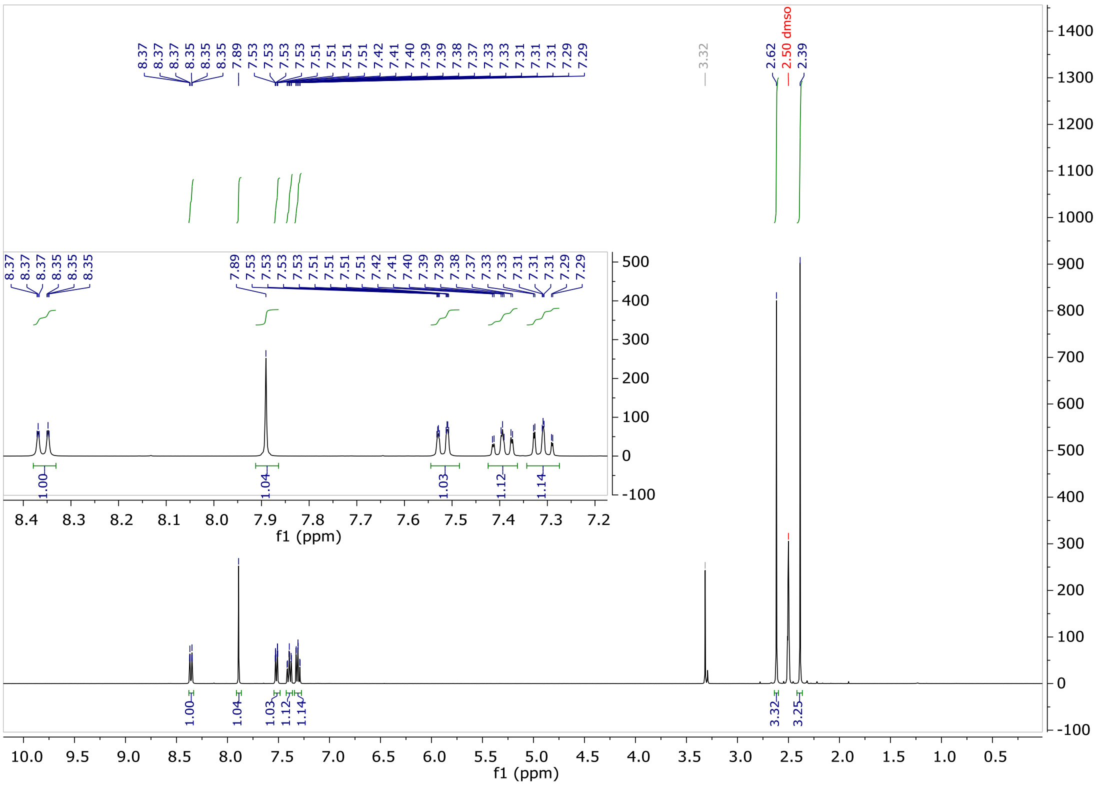

Figure S1. ^{1}H-NMR of **2**.

*1-Acetylindolin-3-one (**3**)*

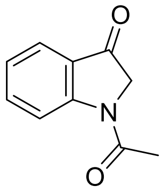

Compound **3** was synthesized following a literature procedure.[26] Compound **2** (500 mg, 2.30 mmol, 1.00 eq.) and Na_2SO_3 (754 mg, 5.99 mmol, 2.60 eq.) were dissolved in ethanol (7 mL) and water (2 mL), and heated to 90 °C for 2 h. The mixture was cooled to ambient temperature, diluted with water (30 mL), and extracted with EtOAc (2 × 50 mL). The combined organic phases were washed with brine (2 x 50 mL) and dried over Na_2SO_4. The solvent was evaporated *in vacuo* and the crude product was purified by flash column chromatography (SiO_2, EtOAc: Petroleum ether, 1:3 to 1:1, v/v). Evaporation of the solvent and drying at high vacuum yielded the title compound as colorless solid (345 mg, 1.99 mmol, 86%).

^{1}H NMR (400 MHz, Chloroform-*d*) δ = 8.54 (d, *J* = 8.4 Hz, 1H), 7.73 (d, *J* = 7.7 Hz, 1H), 7.69 – 7.59 (m, 1H), 7.24 – 7.14 (m, 1H), 4.28 (s, 2H), 2.31 (s, 3H).

R$_f$ (SiO2; EtOAc : Hexanes, 1:2, v/v): 0.21.

The analytical data are in agreement with published data.[26]

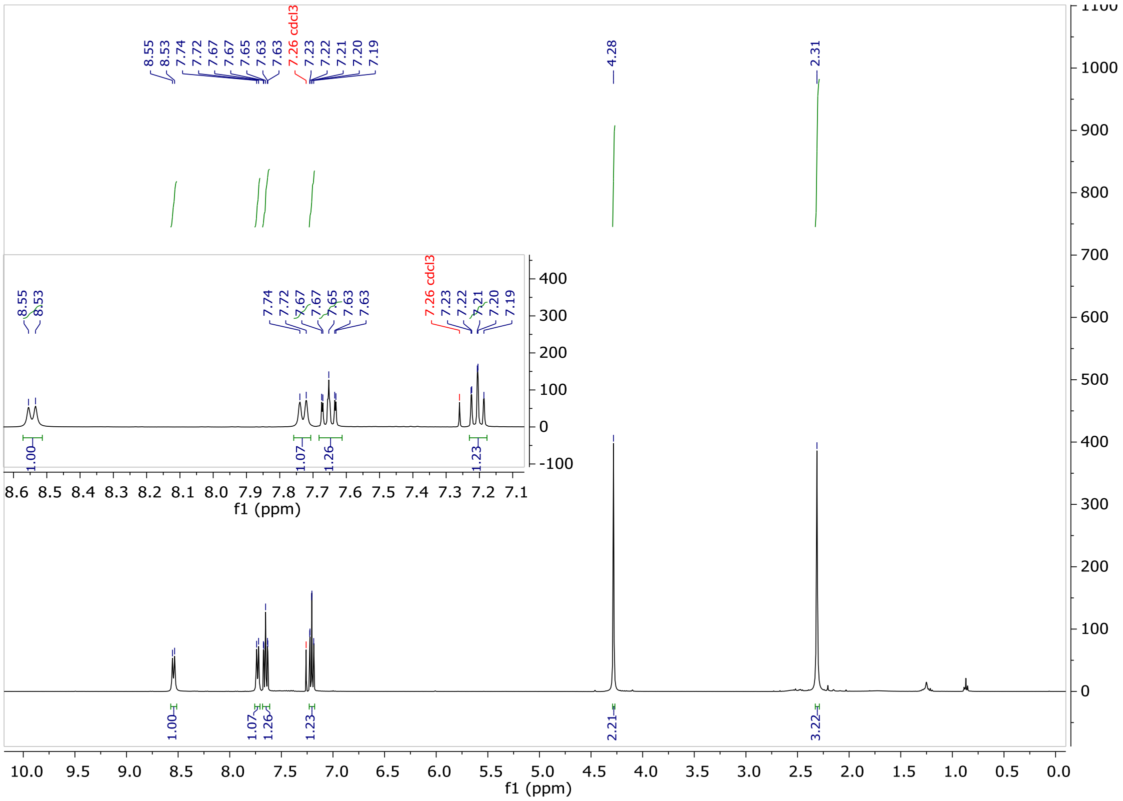

Figure S2. ^{1}H-NMR of **3**.

*(E)-1-Acetyl-2-(phenylimino)indolin-3-one (**PIO**)*

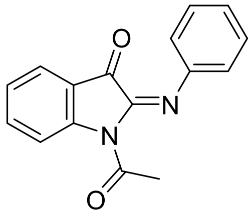

A crimp top vial was heated under vacuum, cooled to ambient temperature, filled with nitrogen atmosphere, and equipped with compound **3** (22 mg, 0.13 mmol, 1 eq) and nitrosobenzene (14 mg, 0.13 mmol, 1 eq). Dry benzene (2 mL) was added to dissolve the reagents. Then, two drops of DBU were added to the reaction mixture, which turned immediately red. The reaction mixture was stirred for one minute, completion was confirmed by TLC. Subsequently the mixture was diluted with EtOAc (20 mL) and poured onto water (20 mL). The phases were separated, and the organic phase was washed with water (20 mL), NH_4Cl (aq, sat., 2 × 20 mL), $NaHCO_3$ (aq, sat., 2 × 20 mL), brine (20 mL), and dried over Na_2SO_4. The volatiles were removed *in vacuo* and the pure product was obtained as red crystalline solid (34 mg, 0.13 mmol, *quant.*).

Note: Extensive washing of the organic phase was crucial as **PIO** is sensitive to high concentrations of acid or base. Incomplete removal of the base lead to decomposition during concentration, while heating the water bath of the rotary evaporator to 50 °C, normal phase (pentane, EtOAc), or reversed phase column (water, MeCN with 0.1% formic acid) is tolerated. Sensitivity to acid is particularly pronounced in the presence of a nucleophile, such as methanol, and lead to hydrolysis of the imine.

^{1}H NMR (400 MHz, Methanol-d_4 : $CDCl_3$, 1:1, v/v) δ = 8.49 (d, *J* = 8.4 Hz, 1H), 7.74 – 7.61 (m, 2H), 7.37 – 7.29 (m, 2H), 7.26 (td, *J* = 7.5, 0.8 Hz, 1H), 7.18 – 7.08 (m, 1H), 7.00 – 6.84 (m, 2H), 2.74 (s, 3H).

^{13}C NMR (101 MHz, Methanol-d_4 : $CDCl_3$, 1:1, v/v) δ = 177.4 (q), 71.0 (q), 149.6 (q), 146.3 (q), 143.6 (q), 137.9 (+), 128.6 (+), 125.1 (+), 124.5 (+), 124.3 (+), 121.2 (q), 118.8 (+), 118.2 (+), 27.1 (+).
HRMS (ESI$^+$): calc. ($C_{16}H_{13}N_2O_2^+$): 265.0972, found: 265.0969 (MH$^+$, 5%), 223.0864 ((MH–Ac)$^+$,100%).
IR: ν [cm^{-1}]= 2924 (w), 1728 (m), 1663 (m), 1701 (m), 1605 (m), 1587 (m), 1458 (m), 1328 (m), 1278 (m), 1167 (m), 1098 (w), 909 (w), 756 (s), 600 (m), 537 (m).
Melting Point: 101–103 °C.
R$_f$ (SiO_2; EtOAc:heptane, 3:5, v/v): 0.8, pink spot.

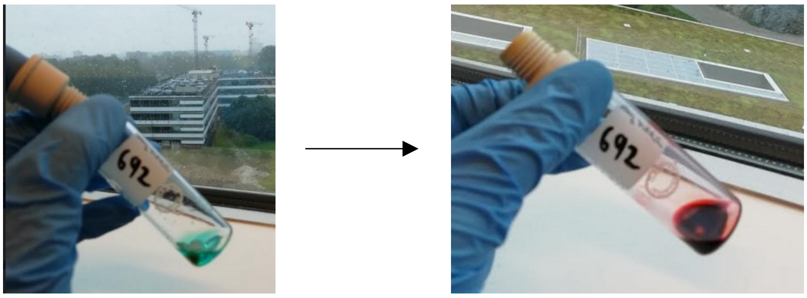

Figure S3. Change of color in the solution observed during the synthesis of **PIO**. A video showing the rapid color change upon addition of DBU is given as separated Supporting file.

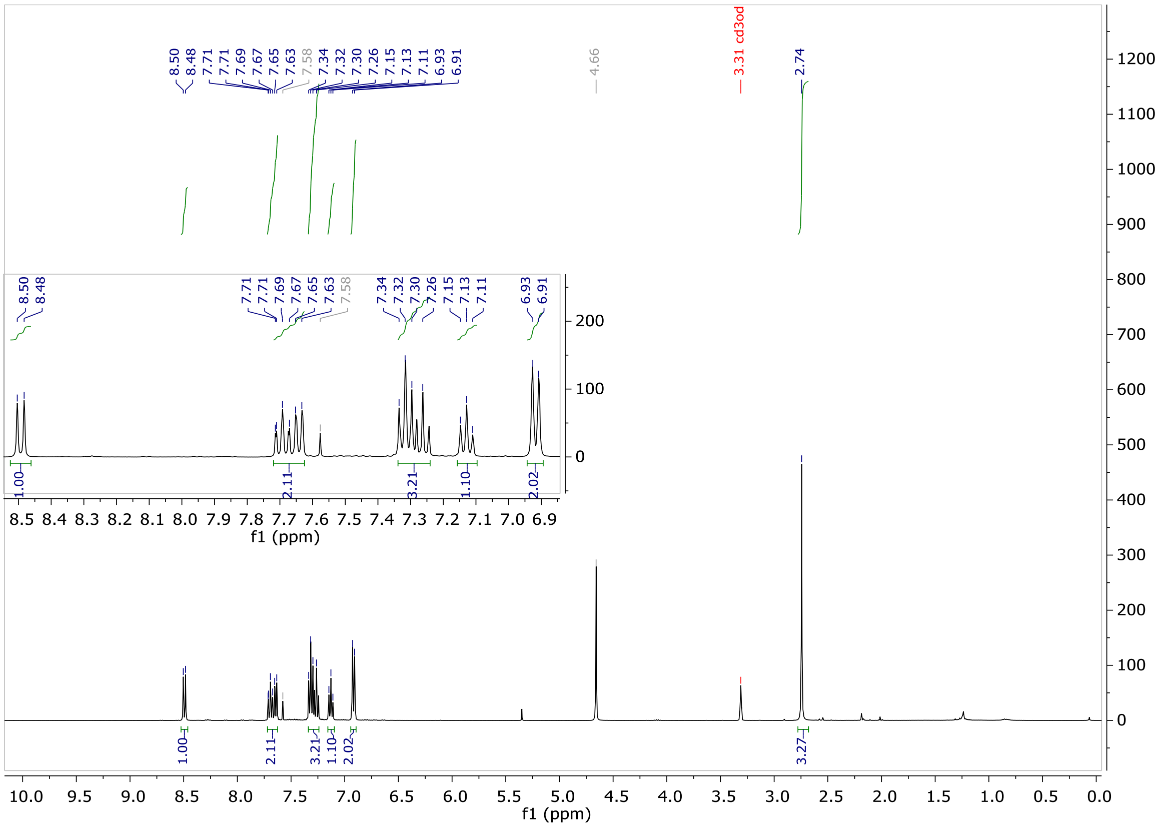

Figure S4. ^{1}H-NMR of *E*-**PIO**.

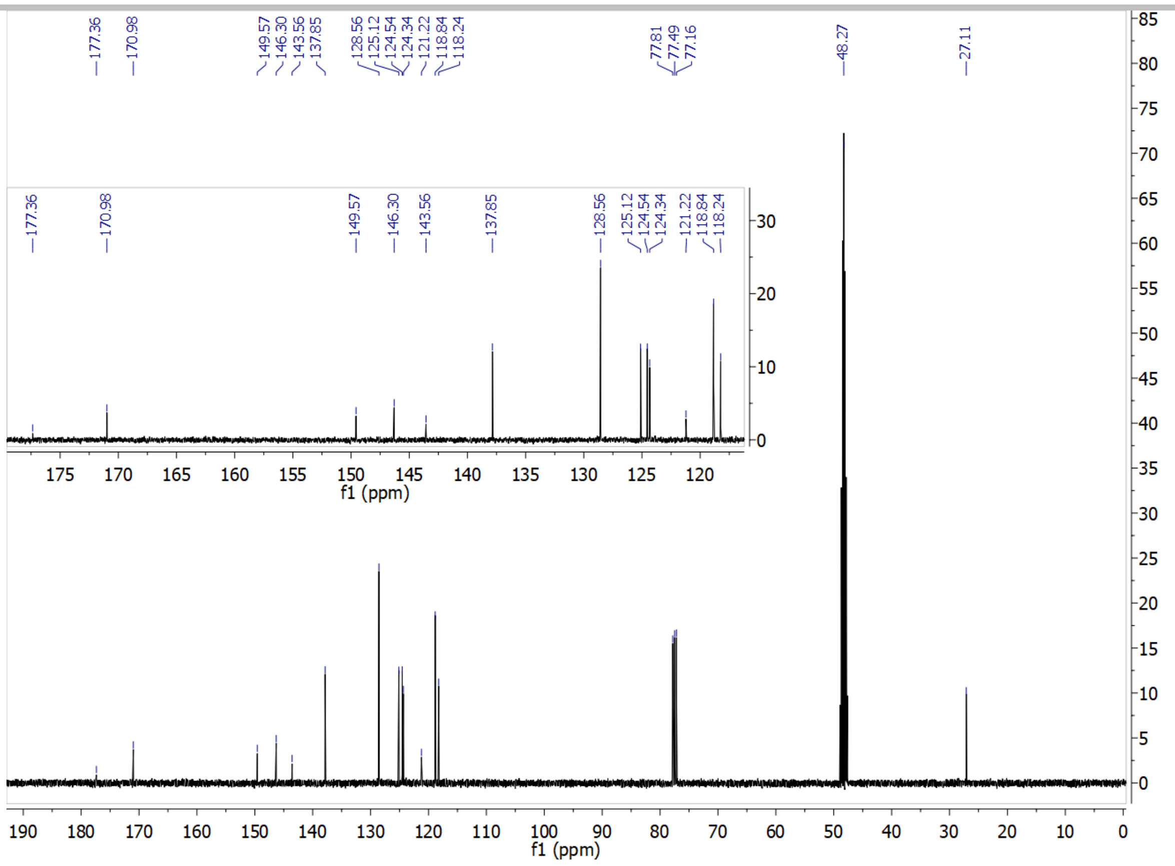

Figure S5. ^{13}C-NMR of *E*-**PIO**.

Nitrosobenzenes

All nitroso benzenes were either freshly prepared or stored in the fridge until usage. Nitrosobenzenes **4**–**7** were synthesized following a procedure our groups published earlier.[12]

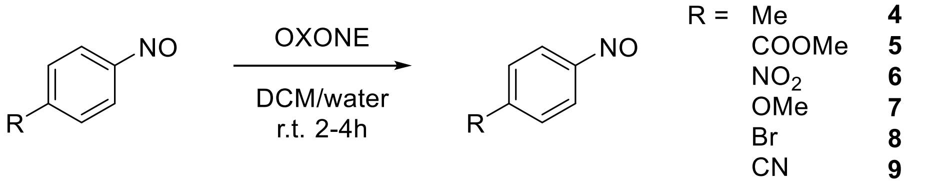

Scheme S2. Overview of the synthesis of nitrosobenzenes used in the study.

1-Bromo-4-nitrosobenzene (**8**)

4-Bromoaniline (1.73 g, 10.1 mmol) and OXONE (6.14 g, 20.0 mmol) were dissolved in DCM (30 mL) and water (40 mL), and vigorously stirred for 3 h at ambient temperature. The process of the reaction was monitored by TLC (SiO_2; EtOAc:heptane, 2:6, v/v). Then, the phases were separated, and the aqueous layer was extracted with DCM (2 x 40 mL). The combined organic extracts were dried over Na_2SO_4 and the volatiles were evaporated in vacuo. The title compound was obtained as yellow powder (862 mg, 4.63 mmol, 46%) and used without further purification.

4-Nitrosobenzonitrile (**9**)

4-Aminobenzonitrile (1.13 g, 9.56 mmol) and OXONE (6.39 g, 20.8 mmol) were dissolved in DCM (30 mL) and water (40 mL), and vigorously stirred for 3 h at ambient temperature. The process of the reaction was monitored by TLC (SiO_2; EtOAc:heptane, 2:6, v/v). Then, the phases were separated, and the aqueous layer was extracted with DCM (2 x 40 mL). The combined organic extracts were dried over Na_2SO_4 and the volatiles were evaporated in vacuo. The title compound was obtained as yellow powder (1.10 g, 8.33 mmol, 87%) and used without further purification.

PIO Derivatives

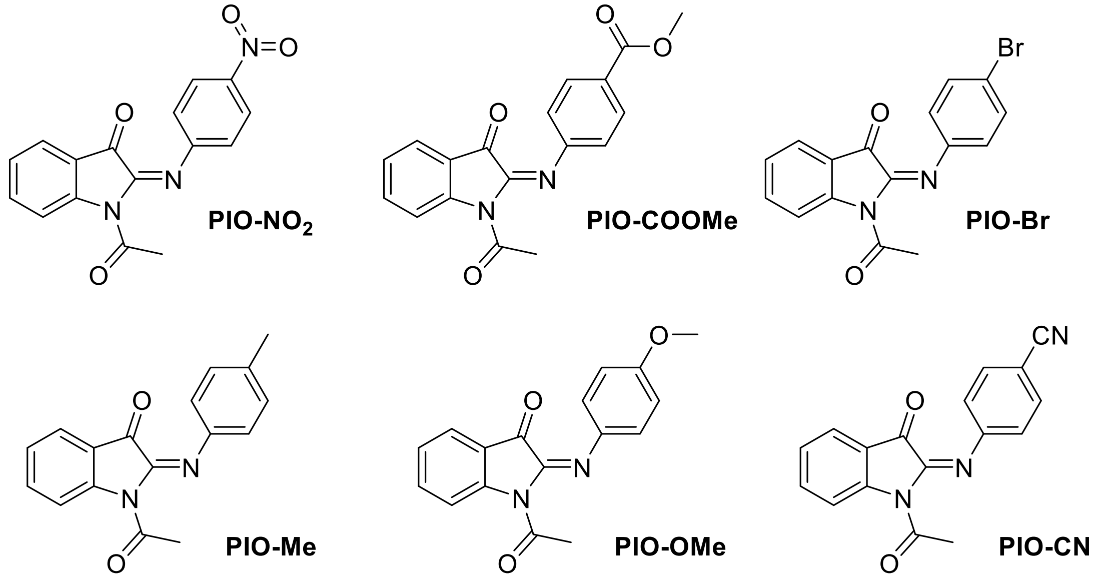

Figure S6. Phenyl substituted **PIO** derivatives.

(E)-1-acetyl-2-(p-tolylimino)indolin-3-one (**PIO-Me**)

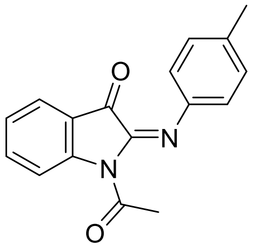

The title compound was synthesized following the same procedure as developed for **PIO** using 1-methyl-4-nitrosobenzene (15.0 mg, 0.125 mmol). After workup, **PIO-Me** was obtained as pink solid (34 mg, 0.12 mmol, 96%).

^{1}H NMR (600 MHz, Acetonitrile-*d*$_3$) δ = 8.46 (dd, *J* = 8.4, 0.8 Hz, 1H), 7.72 (ddd, *J* = 8.6, 7.4, 1.5 Hz, 1H), 7.64 (ddd, *J* = 7.6, 1.5, 0.6 Hz, 1H), 7.29 (td, *J* = 7.5, 0.9 Hz, 1H), 7.16 (AA'BB', 2H), 6.87 (AA'BB', 2H), 2.67 (s, 3H), 2.34 (s, 3H).

^{13}C NMR (151 MHz, Acetonitrile-*d*$_3$) δ = 177.7 (q), 170.6 (q), 149.7 (q), 144.3 (q), 144.0 (q), 137.7 (+), 133.9 (q), 129.2 (+), 125.0 (+), 124.3 (+), 121.5 (q), 119.3 (+), 117.9 (+), 26.8 (+), 20.0 (+).

HRMS (ESI$^+$): calc. $(C_{17}H_{15}N_2O_2–Ac)^+$: 237.1022, found 237.1026 $(MH–Ac)^+$.

IR: ν [cm^{-1}]= 2924 (w), 1696 (s), 1608 (m), 1588 (m), 1374 (m), 1284 (s), 1170 (m), 812 (m), 752 (s), 447 (m).

R$_f$ (SiO_2; EtOAc:heptane, 2:6, v/v): 0.6.

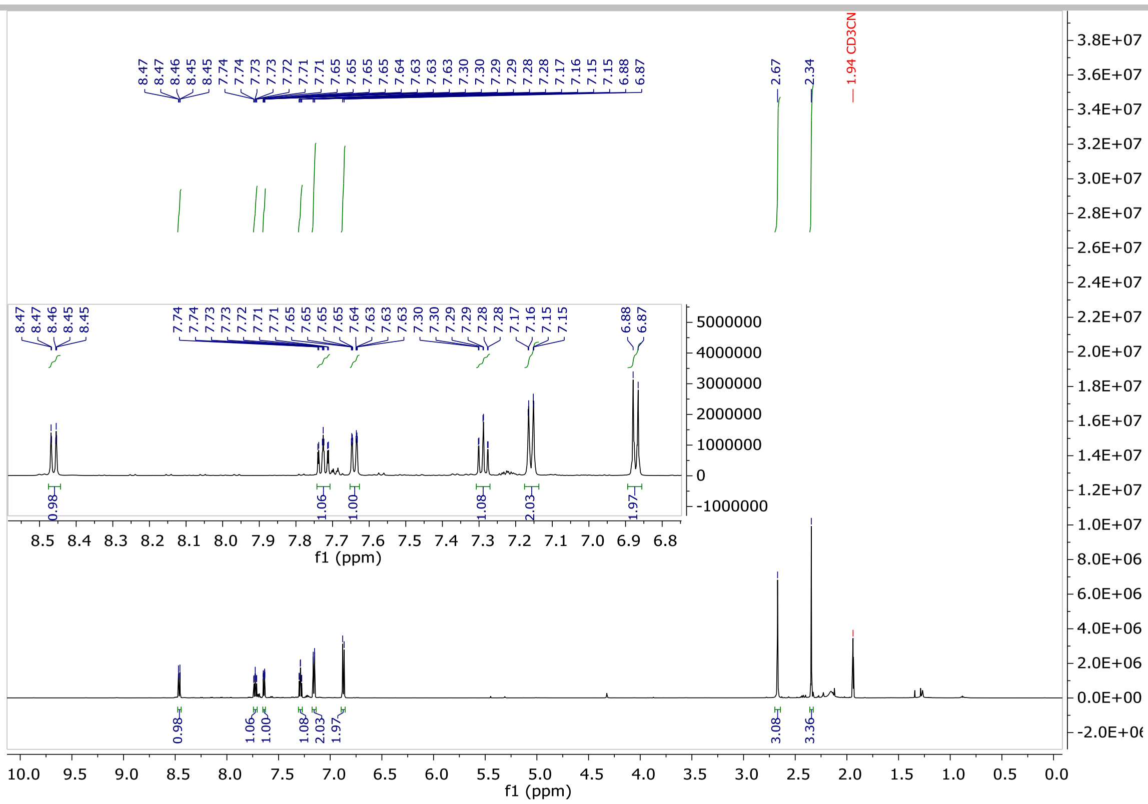

Figure S7. ^{1}H-NMR of *E*-**PIO-Me**.

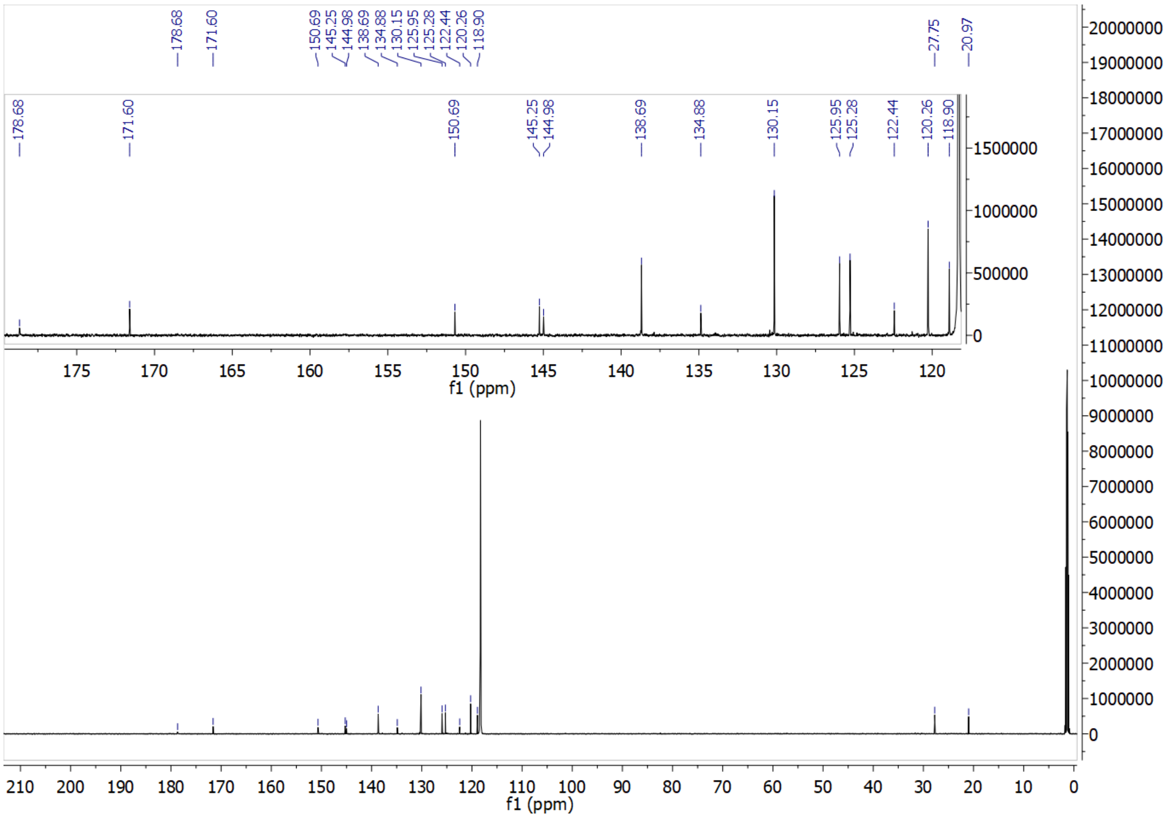

Figure S8. ^{13}C-NMR of *E*-**PIO-Me**.

Methyl (E)-4-((1-acetyl-3-oxoindolin-2-ylidene)amino)benzoate (**PIO-COOMe**)

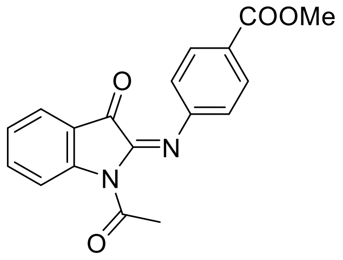

The title compound was synthesized following the same procedure as developed for **PIO** using methyl 4-nitrosobenzoate (17.0 mg, 0.236 mmol). After workup, **PIO-COOMe** was obtained as red solid (19 mg, 0.060 mmol, 59%).

^{1}H NMR (400 MHz, Acetone-d_6) δ = 8.50 (dt, *J* = 8.4, 0.8 Hz, 1H), 7.99 (AA'BB', 2H), 7.79 (ddd, *J* = 8.6, 7.4, 1.4 Hz, 1H), 7.66 (ddd, *J* = 7.5, 1.5, 0.7 Hz, 1H), 7.35 (td, *J* = 7.5, 0.9 Hz, 1H), 7.10 (AA'BB', 2H), 3.88 (s, 3H), 2.74 (s, 3H).

^{13}C NMR (101 MHz, Acetone-d_6) δ = 177.5 (q), 170.1 (q), 166.2 (q), 152.2 (q), 150.0 (q), 143.8 (q), 137.7 (+), 130.2 (+), 125.4 (q), 125.1 (+), 124.3 (+), 121.2 (q), 118.8 (+), 118.0 (+), 51.2 (+), 26.7 (+).

HRMS (ESI$^+$): calc. $(C_{18}H_{15}N_2O_4–Ac)^+$: 281.0921, found: 281.0926 $(MH–Ac)^+$.

IR: ν [cm^{-1}]= 2998 (w), 1712 (m), 1696 (m), 1589 (m), 1461 (m), 1374 (m), 1276 (s), 1152 (m), 1099 (m), 981 (w), 919 (w), 767 (m), 693 (m), 580 (w), 444 (m).

Melting Point: 119–122 °C.

R$_f$ (SiO_2; EtOAc:heptane, 2:6, v/v): 0.5.

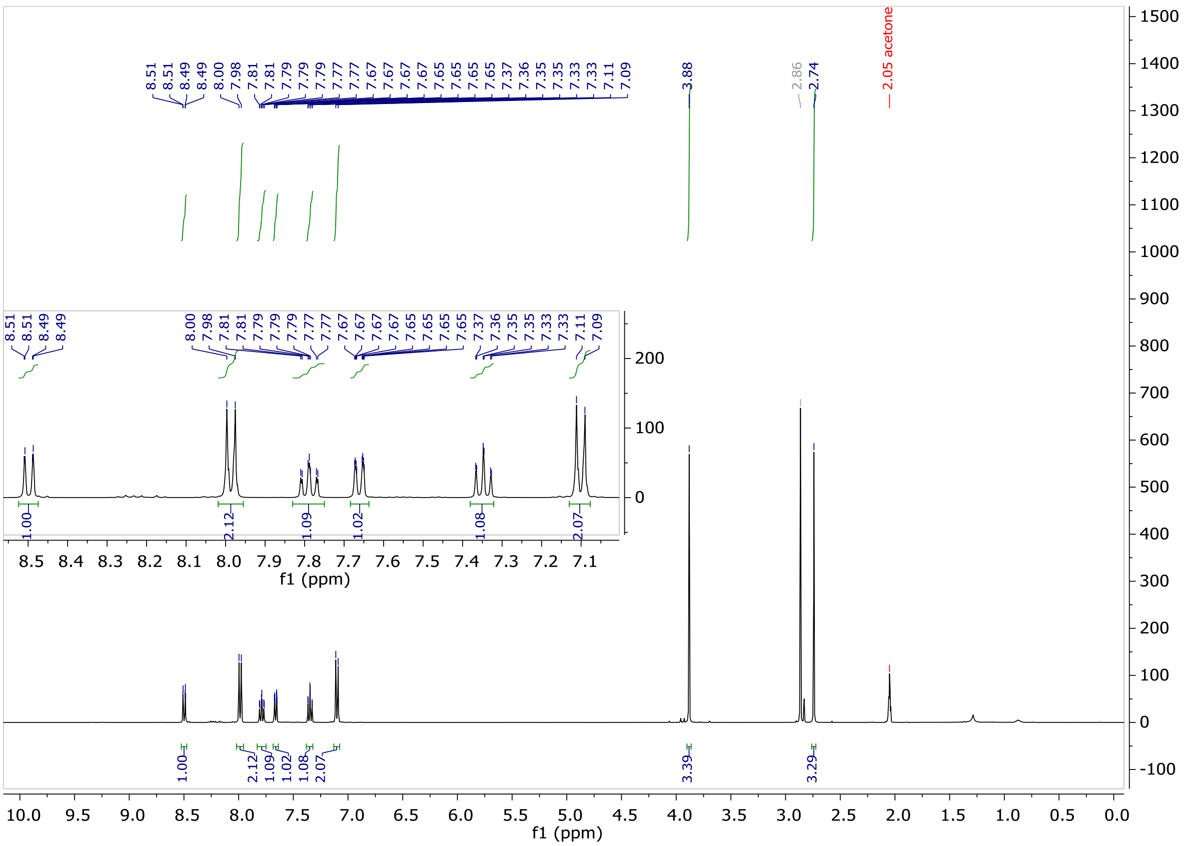

Figure S9. ^{1}H-NMR of *E*-**PIO-COOMe**.

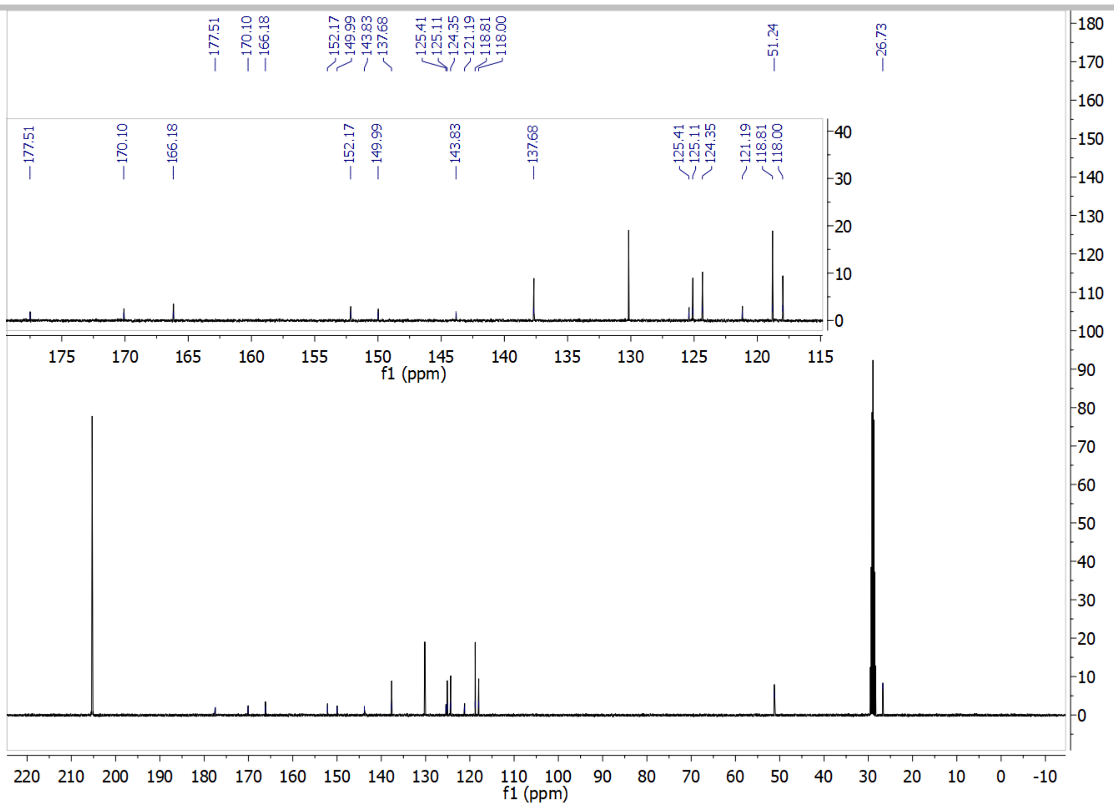

Figure S10. ^{13}C-NMR of *E*-**PIO-COOMe**.

(E)-1-acetyl-2-((4-nitrophenyl)imino)indolin-3-one (**PIO-NO$_2$**)

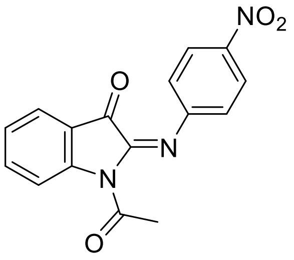

The title compound was synthesized following the same procedure as developed for **PIO** using 1-nitro-4-nitrosobenzene (16 mg, 0.108 mmol). After workup, **PIO-NO$_2$** was purified by automated flash column chromatography (SiO_2, EtOAc: heptane, 2:8 to 1:1, v/v) and isolated as dark red solid (15 mg, 0.048 mmol, 45%).

^{1}H NMR (600 MHz, Acetonitrile-d_3) δ = 8.50 (dt, *J* = 8.4, 0.8 Hz, 1H), 8.25 (AA'BB', 2H), 7.79 (ddd, *J* = 8.4, 7.4, 1.5 Hz, 1H), 7.70 (ddd, *J* = 7.5, 1.5, 0.7 Hz, 1H), 7.35 (td, *J* = 7.5, 0.9 Hz, 1H), 7.10 (AA'BB', 2H), 2.70 (s, 3H).

^{13}C NMR (151 MHz, Acetonitrile-d_3) δ = 178.0 (q), 170.5 (q), 154.2 (q), 150.2 (q), 144.0 (q), 143.8 (q), 138.2 (+), 125.4 (+), 124.8 (+), 124.7 (+), 120.9 (q), 119.1 (+), 118.0 (+), 26.8 (+). One quarterly carbon could not be resolved.

HRMS (ESI$^+$): calc. ($C_{16}H_{12}N_3O_4$–Ac)$^+$: 268.0717, found: 268.0720 (MH–Ac)$^+$.

IR: ν [cm^{-1}]= 3321 (w), 1734 (w), 1682 (s). 1584 (s), 1507 (m), 1462 (m), 1327 (s), 1285 (s), 1174 (s), 1154 (m), 1103 (m), 850 (m), 757 (s), 689 (m), 442 (s).

Melting Point: 173–175 °C.

R$_f$ (SiO_2; EtOAc:heptane, 2:6, v/v): 0.4.

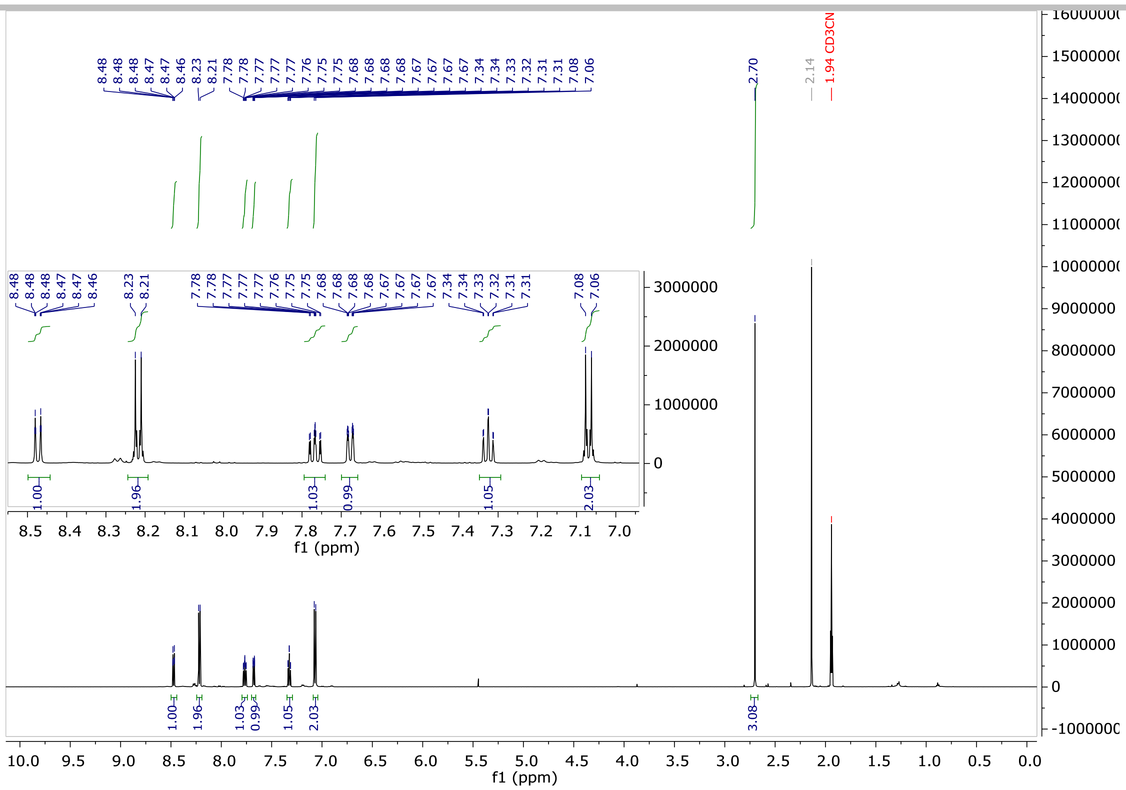

Figure S11. ^{1}H-NMR of *E*-**PIO-NO$_2$**.

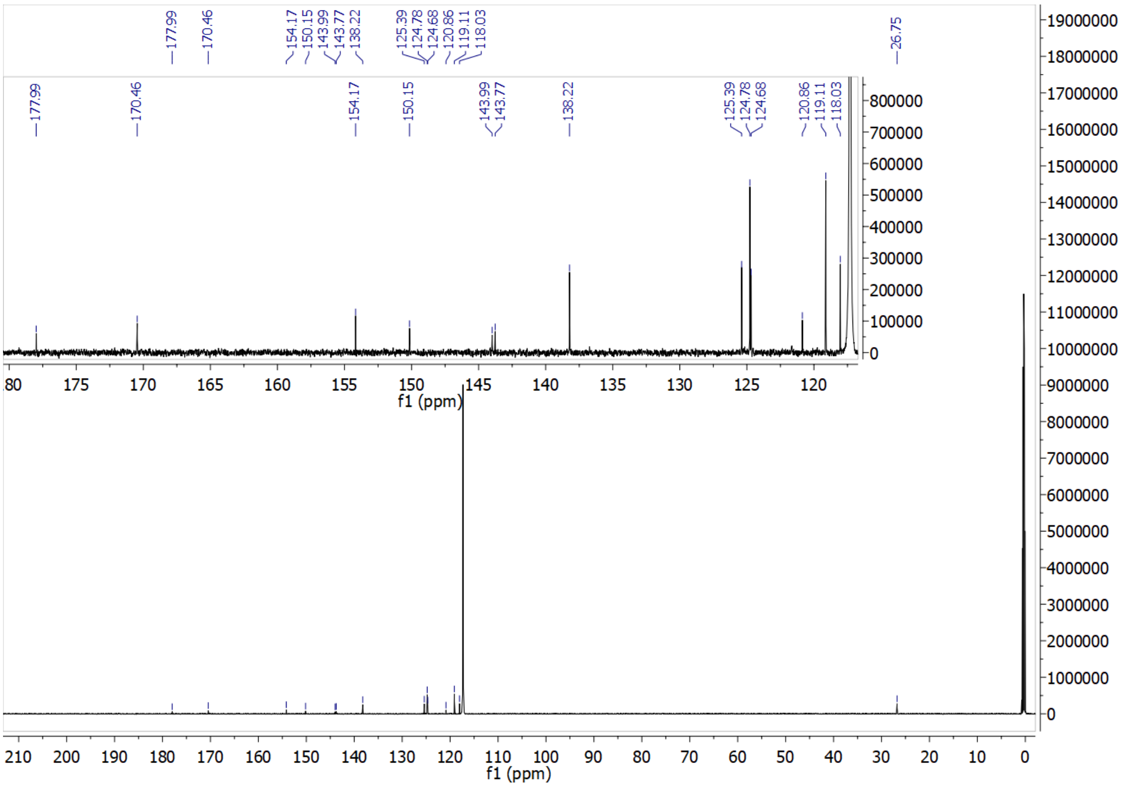

Figure S12. ^{13}C-NMR of *E*-**PIO-NO$_2$**.

(E)-1-acetyl-2-((4-bromophenyl)imino)indolin-3-one (**PIO-Br**)

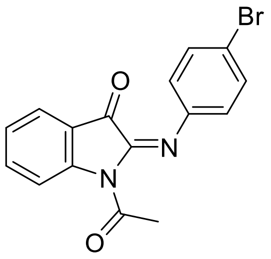

The title compound was synthesized following the same procedure as developed for **PIO** using 1-bromo-4-nitrosobenzene (18 mg, 0.098 mmol). After workup, **PIO-Br** was purified by automated flash column chromatography (SiO_2, EtOAc: heptane, 2:8 to 1:1, v/v) and isolated as pink solid (26 mg, 0.076 mmol, 77%).

^{1}H NMR (600 MHz, Acetonitrile-*d*$_3$) δ = 8.48 (dt, *J* = 8.4, 0.8 Hz, 1H), 7.79 – 7.73 (m, 1H), 7.68 (ddd, *J* = 7.6, 1.5, 0.7 Hz, 1H), 7.50 (AA'BB', 2H), 7.36 – 7.30 (m, 1H), 6.91 (AA'BB', 2H), 2.70 (s, 3H).

^{13}C NMR (151 MHz, Acetonitrile-*d*$_3$) δ = 177.8 (q), 170.5 (q), 149.9 (q), 146.5 (q), 144.3 (q), 137.9 (+), 131.6 (+), 125.2 (+), 124.5 (+), 121.2 (q), 121.0 (+), 118.0 (+), 116.3 (q), 26.89 (+).

HRMS (ESI$^+$): calc. ($C_{16}H_{12}BrN_2O_2$–Ac)$^+$: 300.9971 (for ^{79}Br) and 302.9951 (for ^{81}Br), found: 300.9971 ((MH–Ac))$^+$, ^{79}Br), 302.9951 ((MH–Ac))$^+$, ^{81}Br).

IR: ν [cm^{-1}]= 1723 (m), 1704 (m), 1670 (m), 1605 (m), 1588 (m), 1475 (m), 1456 (m), 1306 (m), 1278 (s), 1144 (m). 824 (m), 758 (s). 545 (m), 440 (m).

Melting Point: 114–117 °C.

R$_f$ (SiO_2; EtOAc:heptane, 2:6, v/v): 0.6.

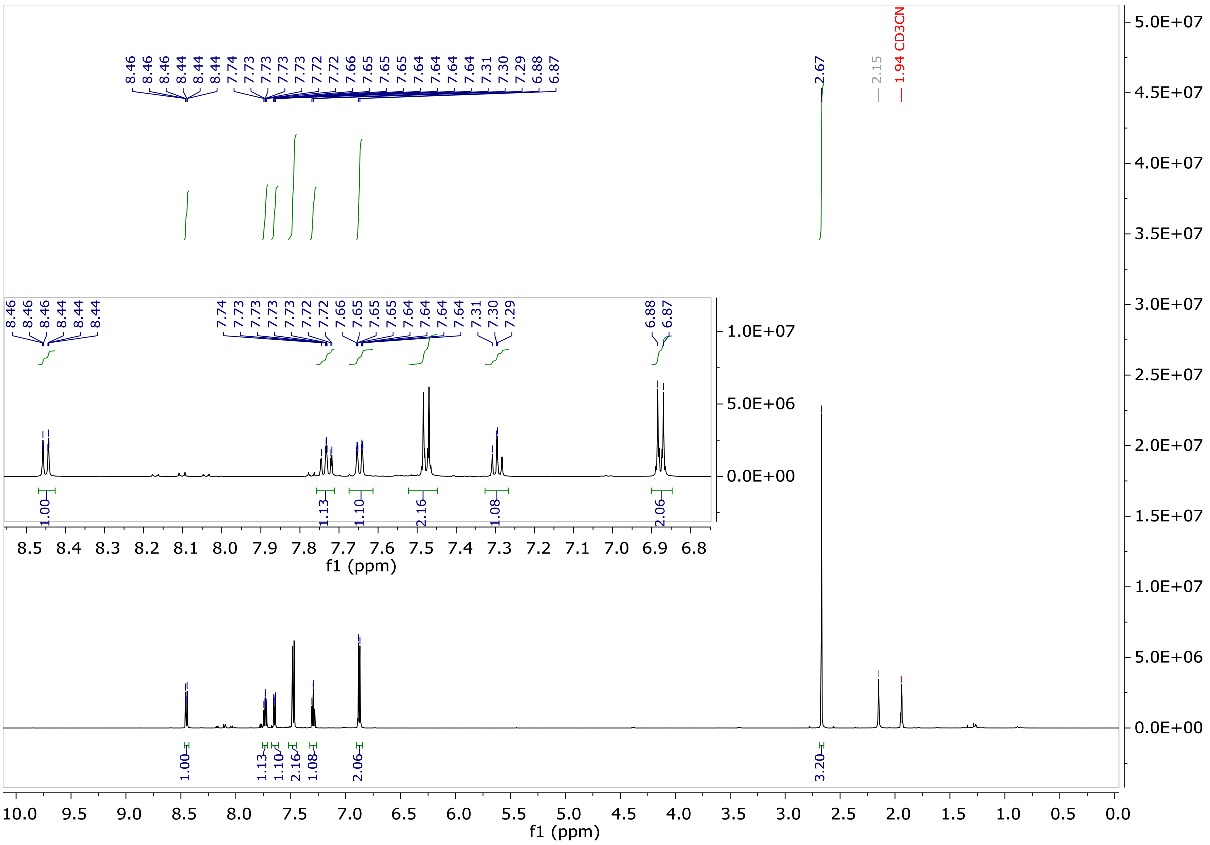

Figure S13. ^{1}H-NMR of *E*-**PIO-Br**.

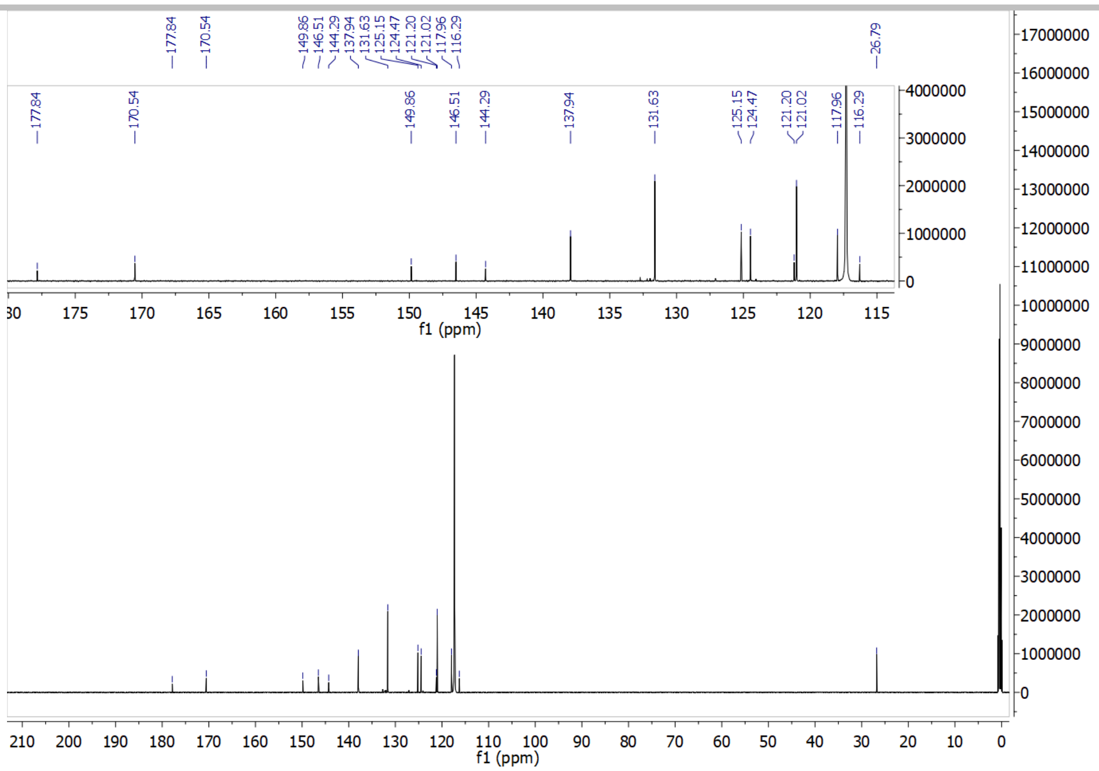

Figure S14. ^{13}C-NMR of *E*-**PIO-Br**.

(E)-4-((1-acetyl-3-oxoindolin-2-ylidene)amino)benzonitrile (**PIO-CN**)

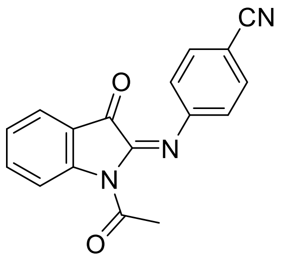

The title compound was synthesized following the same procedure as developed for **PIO** using 4-nitrosobenzonitrile (20.0 mg, 0.151 mmol). After workup, **PIO-CN** was purified by automated flash column chromatography (SiO_2, EtOAc: heptane, 2:8 to 1:1, v/v) and isolated as red solid (16 mg, 0.054 mmol, 36%).

^{1}H NMR (400 MHz, Acetone-d_6) δ = 8.53 – 8.46 (m, 1H), 7.80 (ddd, *J* = 8.4, 7.4, 1.5 Hz, 2H), 7.73 (AA'BB', 2H), 7.67 (ddd, *J* = 7.7, 1.5, 0.7 Hz, 1H), 7.36 (td, *J* = 7.5, 0.8 Hz, 2H), 7.19 (AA'BB', 2H), 2.74 (s, 3H).

^{13}C NMR (101 MHz, Acetone-d_6) δ = 177.6 (q), 170.1 (q), 152.1 (q), 150.0 (q), 144.0 (q), 137.8 (+), 132.8 (+), 125.2 (+), 124.4 (+), 121.1 (q), 119.8 (+), 118.9 (q), 118.0 (+), 106.6 (q), 26.7 (+).

HRMS (ESI$^+$): calc. $(C_{17}H_{12}N_3O_2\text{–Ac})^+$: 248.0818, found: 248.0821 (MH–Ac)$^+$.

IR: ν [cm^{-1}]= 2992 (w), 2230 (m), 1730 (m), 1704 (s), 1675 (m), 1593 (s), 1560 (m), 1375 (m), 1332 (s), 1282 (s), 1101 (m), 980 (m), 239 (m), 756 (m), 559 (s), 444 (s).

Melting Point: 115–117 °C.

R$_f$ (SiO_2; EtOAc:heptane, 2:6, v/v): 0.4.

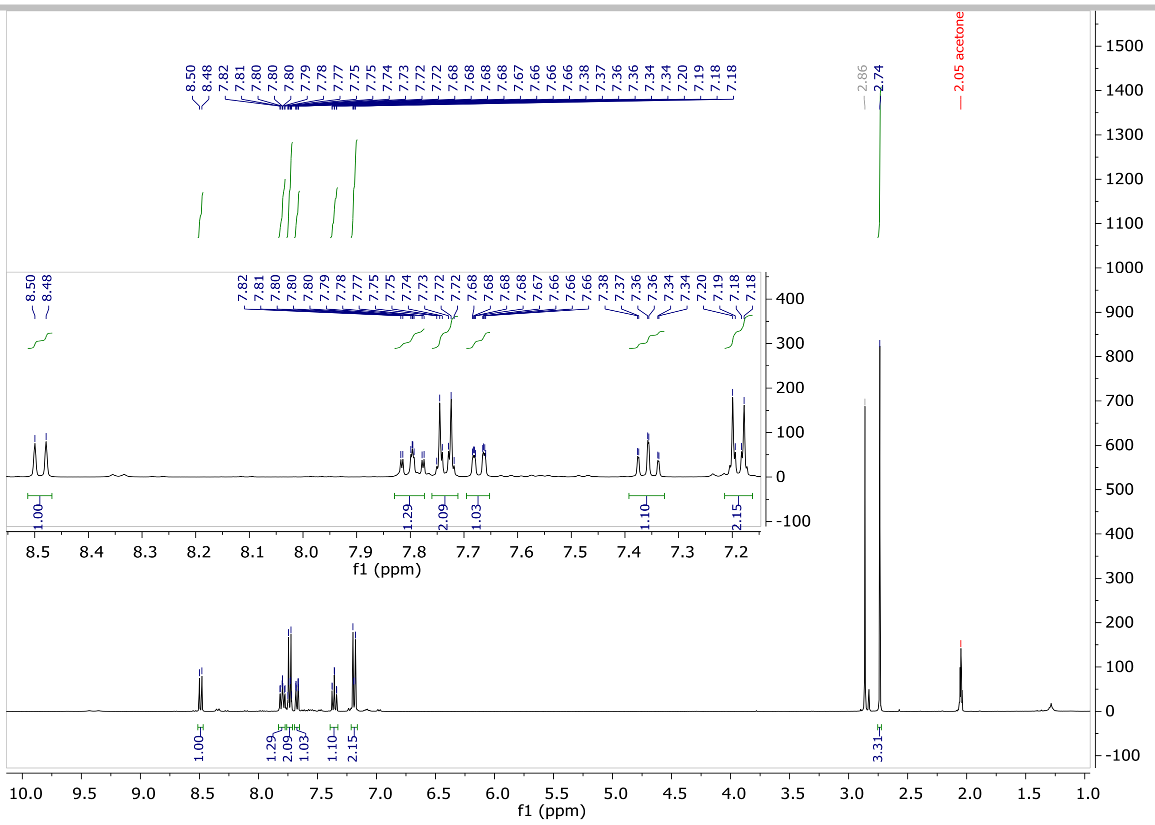

Figure S15. ^{1}H-NMR of *E*-**PIO-CN**.

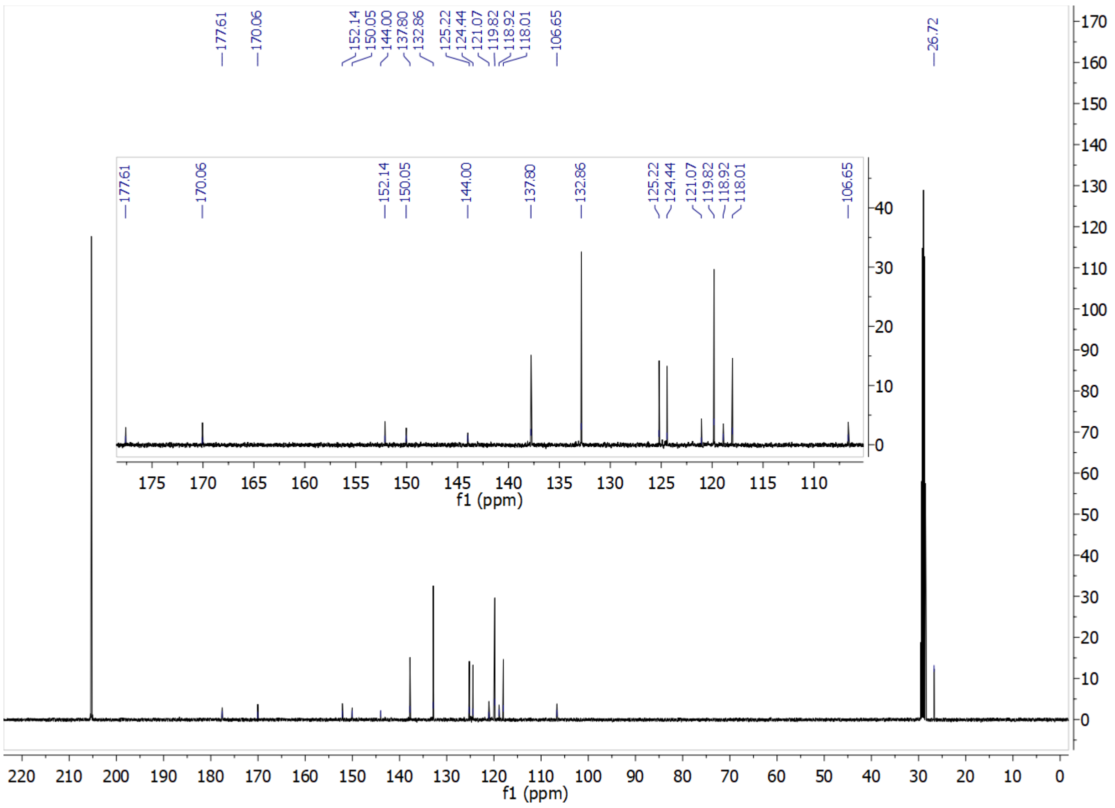

Figure S16. ^{13}C-NMR of *E*-**PIO-CN**.

(E)-1-acetyl-2-((4-methoxyphenyl)imino)indolin-3-one (**PIO-OMe**)

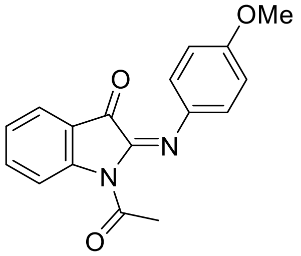

The title compound was synthesized following the same procedure as developed for **PIO** using 1-methoxy-4-nitrosobenzene (32 mg, 0.236 mmol). After workup, **PIO-OMe** was purified by automated flash column chromatography (SiO_2, EtOAc: heptane, 2:8 to 1:1, v/v) and isolated as purple solid (15 mg, 0.051 mmol, 22%).

^{1}H NMR (400 MHz, Acetone-d_6) δ = 8.54 – 8.41 (m, 1H), 7.76 (ddd, *J* = 8.6, 7.4, 1.5 Hz, 1H), 7.66 (ddd, *J* = 7.5, 1.6, 0.7 Hz, 1H), 7.32 (td, *J* = 7.5, 0.9 Hz, 1H), 7.11 (AA'BB', 2H), 6.91 (AA'BB', 2H), 3.82 (s, 3H), 2.70 (s, 3H).

^{13}C NMR (101 MHz, Acetone-d_6) δ = 177.2 (q, signal was confirmed by 2D techniques), 170.2 (q), 157.5 (q), 149.4 (q), 144.0 (q), 139.2 (q), 137.3 (+), 124.8 (+), 124.1 (+), 122.0 (+), 121.6 (q), 117.9 (+), 113.6 (+), 54.8 (+), 26.7 (+).

HRMS (ESI$^+$): calc. ($C_{17}H_{15}N_2O_3$–Ac)$^+$: 253.0972, found: 253.0975 (MH–Ac)$^+$.

IR: ν [cm^{-1}]= 2925 (w), 1719 (m), 1691 (m), 1606 (m), 1503 (m), 1461 (m), 1330 (m), 1283 (m), 1243 (s), 1167 (s), 1099 (m), 1026 (m), 826 (m), 758 (s), 590 (m), 443 (m).

Melting Point: 96–99 °C.

R$_f$ (SiO_2; EtOAc:heptane, 2:6, v/v): 0.6.

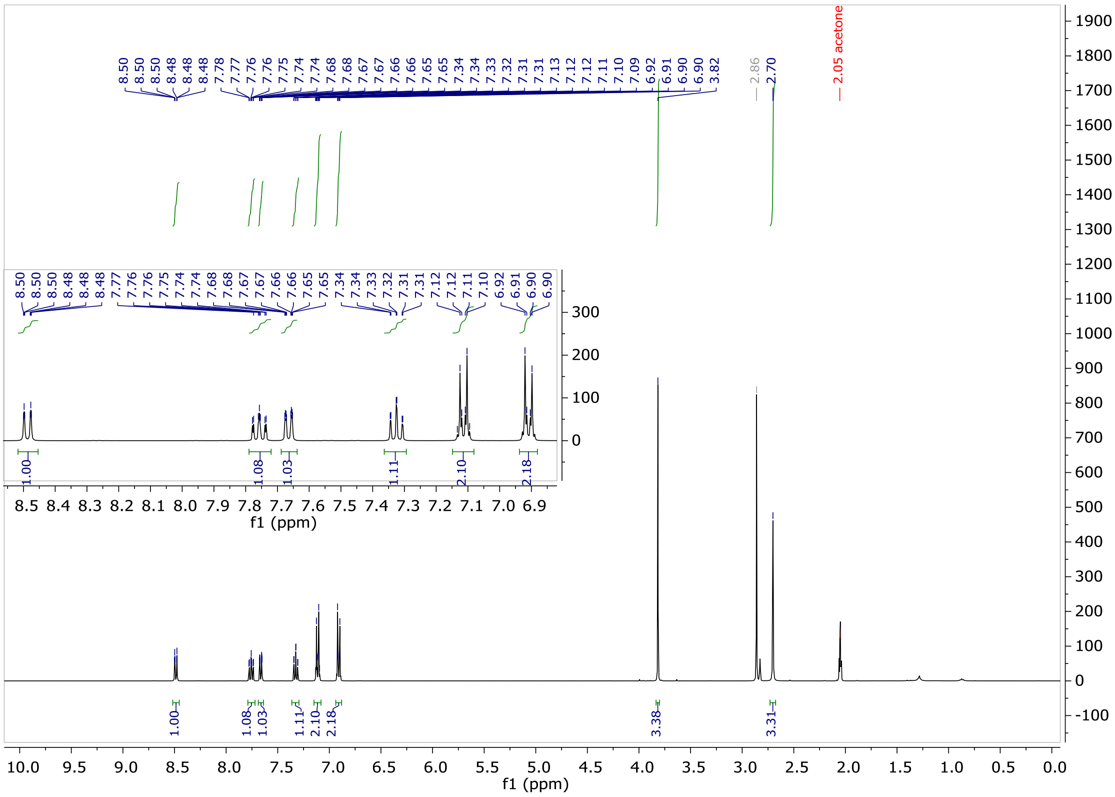

Figure S17. ^{1}H-NMR of *E*-**PIO-OMe**.

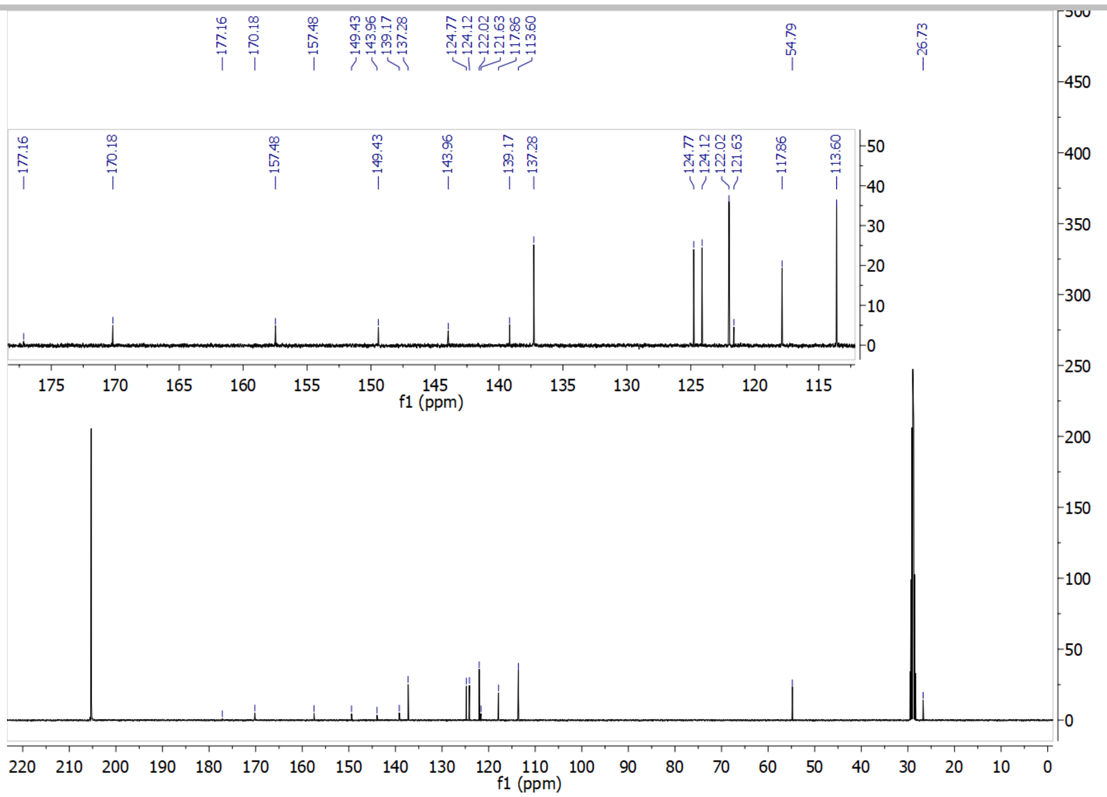

Figure S18. ^{13}C-NMR of *E*-**PIO-OMe**.

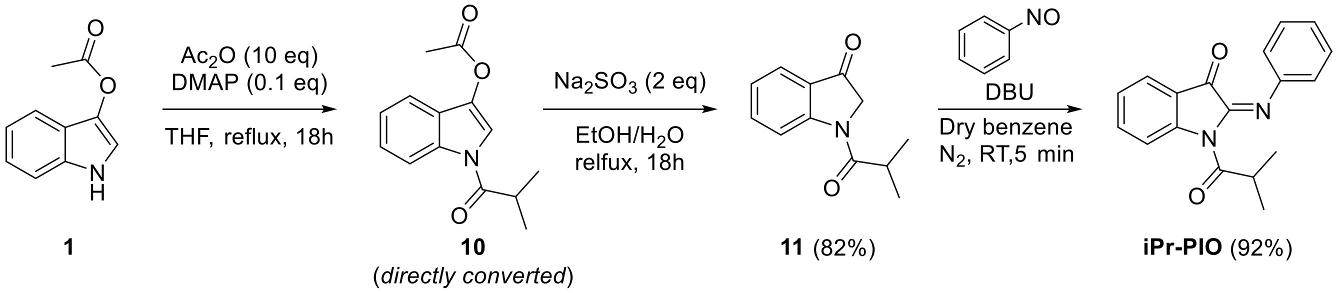

Scheme S3. Overview of the synthetic route towards *E*-**iBu-PIO**.

1-Isobutyrylindolin-3-one (**11**)

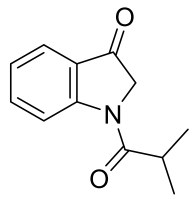

The title compound was synthesized analogously to compound **3**.
A crimp top vial was charged with 1H-indol-3-yl acetate (500 mg, 2.85 mmol, 1.00 eq.) and DMAP (49 mg, 0.40 mmol, 0.14 eq.) dissolved in THF (16 mL). Acetic anhydride (4.5 g, 4.7 mL, 28 mmol, 10.0 eq.) was added, the vial was sealed, and the mixture was heated to 85 °C for 16 h. The mixture was cooled to ambient temperature, diluted with EtOAc (30 mL), and poured onto NH_4Cl (aq, sat, 50 mL). The phases were separated, and the organic phase was washed with brine (2 × 30 mL). The organic layer was dried over Na_2SO_4 and the volatiles were evaporated. The crude product **10** was converted without further purification.
Compound **10** (350 mg, 1.43 mmol, 1.00 eq.) and Na_2SO_3 (360 mg, 2.85 mmol, 2.00 eq.) were dissolved in ethanol (5 mL) and water (2 mL) and heated to 90 °C for 18 h. The mixture was cooled to ambient temperature, diluted with water (30 mL) and extracted with EtOAc (2 × 30 mL). The combined organic phases were washed with brine (2 x 30 mL) and dried over Na_2SO_4. The solvent was evaporated *in vacuo* and title compound as colorless solid (237 mg, 1.17 mmol, 82% over two steps) and used without further purification.
^{1}H NMR (400 MHz, Acetone-d_6) δ = 8.59 (d, *J* = 8.4 Hz, 1H), 7.73 – 7.63 (m, 2H), 7.31 – 7.15 (m, 1H), 4.54 (s, 3H), 2.99 (hept, J = 6.7 Hz,, 1H), 1.21 (d, *J* = 6.7 Hz, 6H).

^{13}C NMR (101 MHz, Acetone-d_6) δ = 195.0 (q), 175.1 (q), 136.5 (+), 125.3 (q), 123.5 (+), 122.9 (+), 118.4 (+), 55.3 (-), 33.4 (+), 18.6 (+). One quarterly carbon signal could not be resolved.
ESI-MS (ESI$^+$): calc. ($C_{12}H_{14}NO_2^+$): 204.1, found: 204.1 (MH$^+$).
IR: ν [cm^{-1}]= 3130 (w), 2976 (w), 2935 (w), 1721 (m), 1681 (s), 1600 (m), 1528 (w), 1461 (s), 1392 (m), 1328 (m), 1156 (m), 1253 (s), 1190 (m), 1042 (m), 921 (w), 882 (w), 852 (w), 758 (s), 617 (w), 578 (m), 514 (m), 410 (w).
Melting Point: 52–56 °C.
R$_f$ (SiO_2; EtOAc:heptane, 2:6, v/v): 0.4.

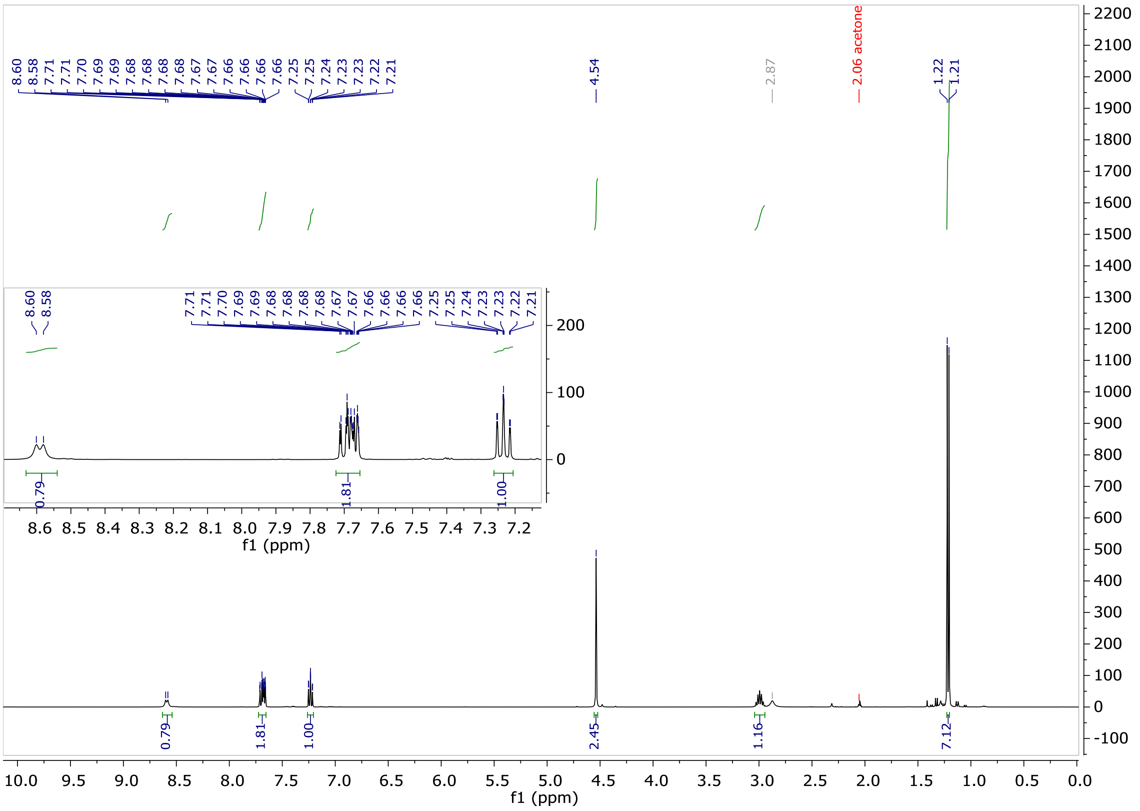

Figure S19. ^{1}H-NMR of **11**.

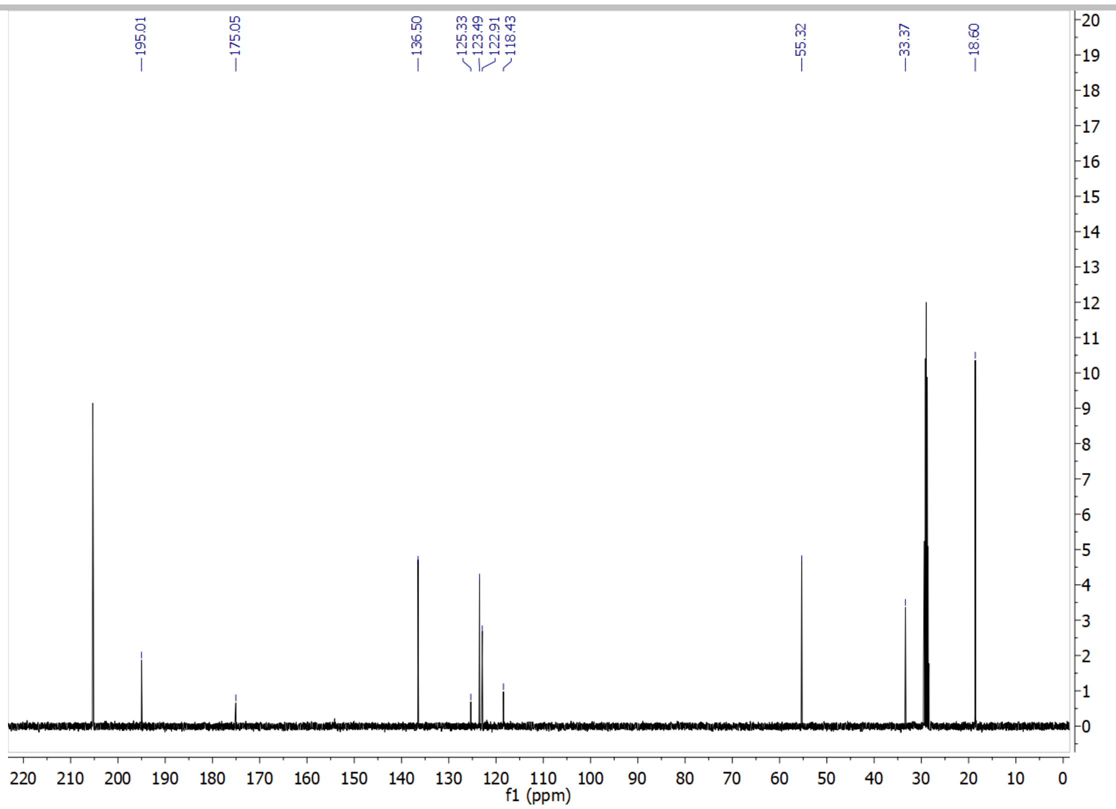

Figure S20. ^{13}C-NMR of **11**.

(E)-1-isobutyryl-2-(phenylimino)indolin-3-one (**iBu-PIO**)

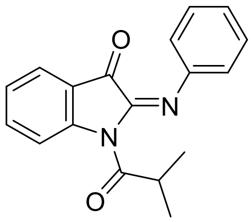

The title compound was synthesized following the same procedure as developed for **PIO** using compound **5** (38 mg, 0.187 mmol) and nitrosobenzene (20 mg, 0.187 mmol). After workup, **iBu-PIO** was obtained without further purification (51 mg, 0.17 mmol, 92%).

^{1}H NMR (400 MHz, Acetone-d_6) δ = 8.48 (d, *J* = 8.4 Hz, 1H), 7.75 (ddd, *J* = 8.6, 7.4, 1.5 Hz, 1H), 7.64 (ddd, *J* = 7.5, 1.5, 0.7 Hz, 1H), 7.39 – 7.28 (m, 3H), 7.21 – 7.11 (m, 1H), 7.07 – 7.02 (m, 2H), 4.13 (h, *J* = 6.8 Hz, 1H), 1.27 (d, *J* = 6.8 Hz, 6H).

^{13}C NMR (101 MHz, Acetone-d_6) δ = 177.7 (q), 177.3 (q), 150.2 (q), 147.1 (q), 143.5 (q), 137.4 (+), 128.5 (+), 124.8 (+), 124.2 (+), 123.8 (+), 121.5 (q), 119.2 (+), 118.12 (+), 34.8 (+), 18.6 (+).

HRMS (ESI$^+$): calc. (($C_{18}H_{17}N_2O_2$–^{i}Pr(CO))$^+$: 223.0866, found: 223.0867 (MH - ^{i}Pr(CO))$^+$.

IR: ν [cm^{-1}]= 2985 (w), 1730 (m), 1699 (m), 1657 (m), 1607 (m), 1589 (m), 1462 (m), 1382 (w), 1353 (w), 1305 (m), 1268 (m), 1169 (m), 1119 (m), 989 (w), 913 (w), 759 (s), 692 (m), 645 (w), 442 (m).

Melting Point: 89–92 °C.

R$_f$ (SiO_2; EtOAc:heptane, 2:6, v/v): 0.7.

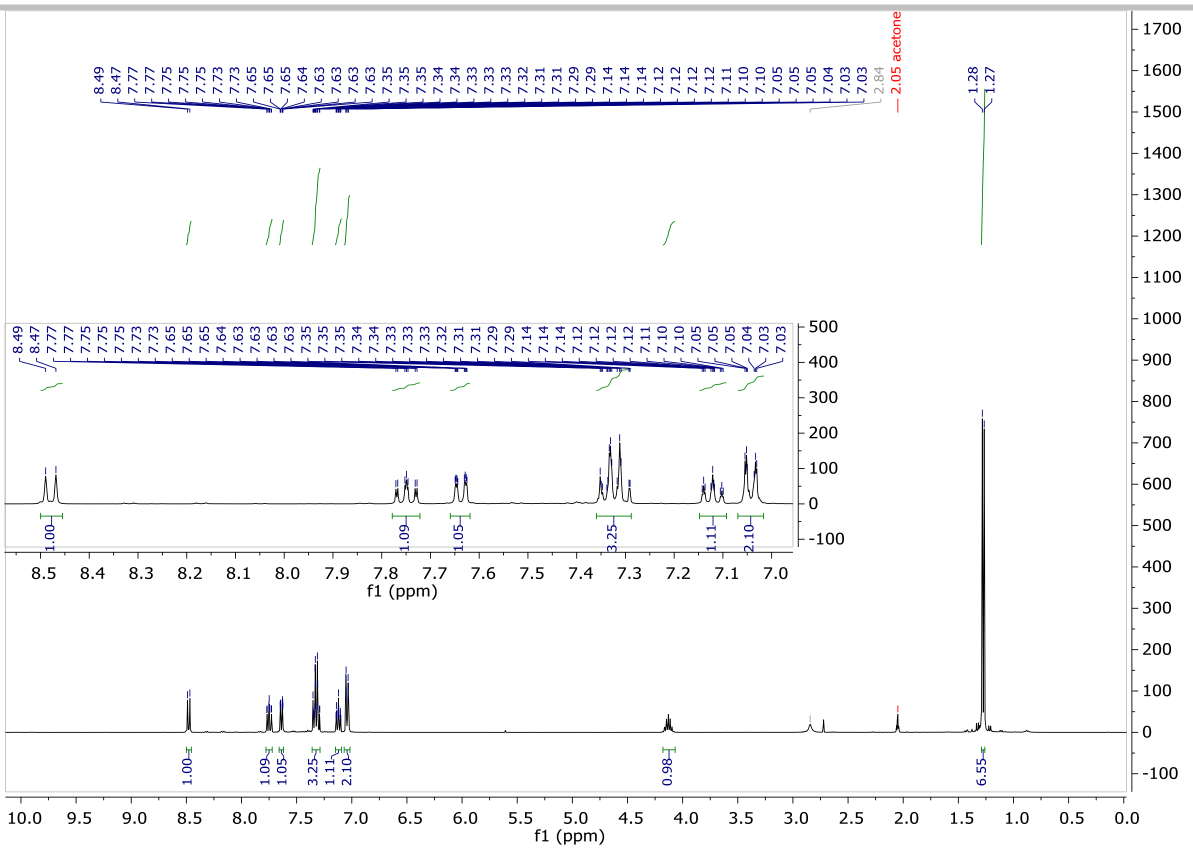

Figure S21. ^{1}H-NMR of **iBu-PIO**.

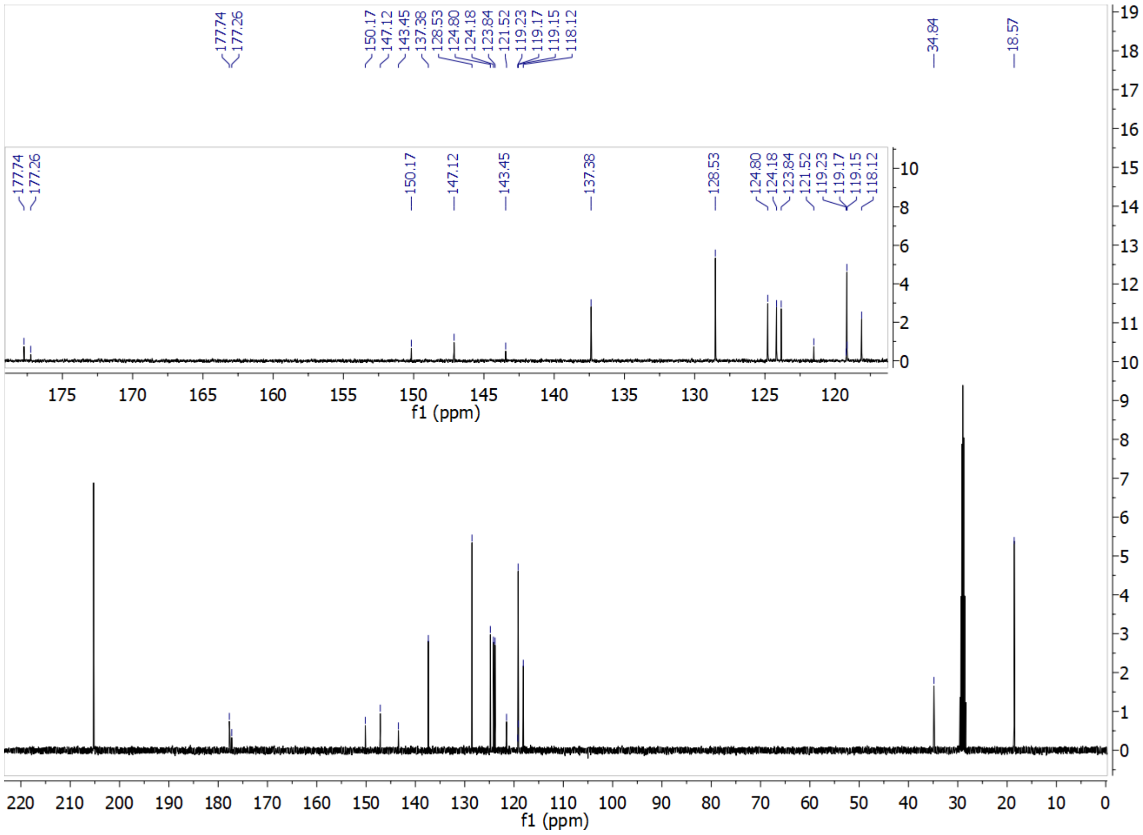

Figure S22. ^{13}C-NMR of **iBu-PIO**.

UV-Vis characterization

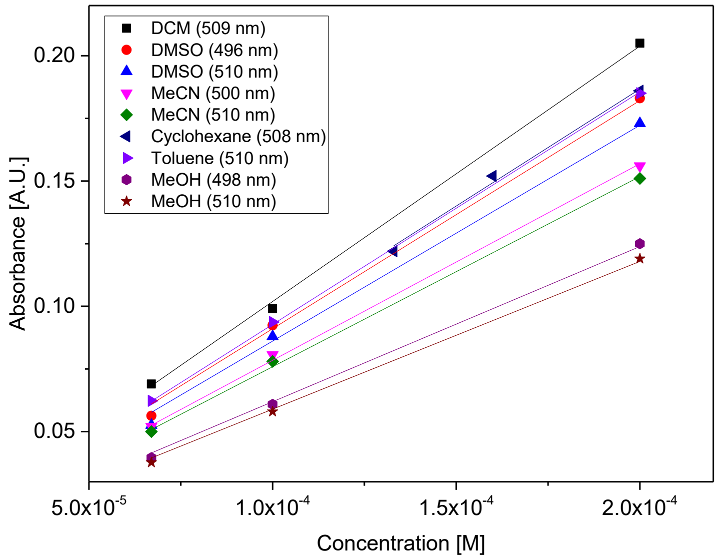

Figure S23. Absorbance of the lowest-energy absorption band of *E*-**PIO** at its λ_{max} at different concentrations in different solvents. The intercept of the linear fit was fixed at the origin.

Table S1. Linear regression of the absorbances plotted in Figure S23.

	Slope (ε [M^{-1} cm^{-1}])	R^2 (COD)
DCM (509 nm)	1019.15983 ± 9.48641	0.99983
DMSO (496 nm)	910.49753 ± 15.07182	0.99945
DMSO (510 nm)	861.16831 ± 16.62653	0.99926
MeCN (500 nm)	784.45191 ± 7.26233	0.99983
MeCN (510 nm)	758.86876 ± 7.28064	0.99982
Cyclohexane (508 nm)	933.44859 ± 8.52479	0.99983
Toluene (510 nm)	927.6019 ± 3.28603	0.99997
MeOH (498 nm)	619.26627 ± 7.3855	0.99972
MeOH(510)	589.58505 ± 6.99974	0.99972

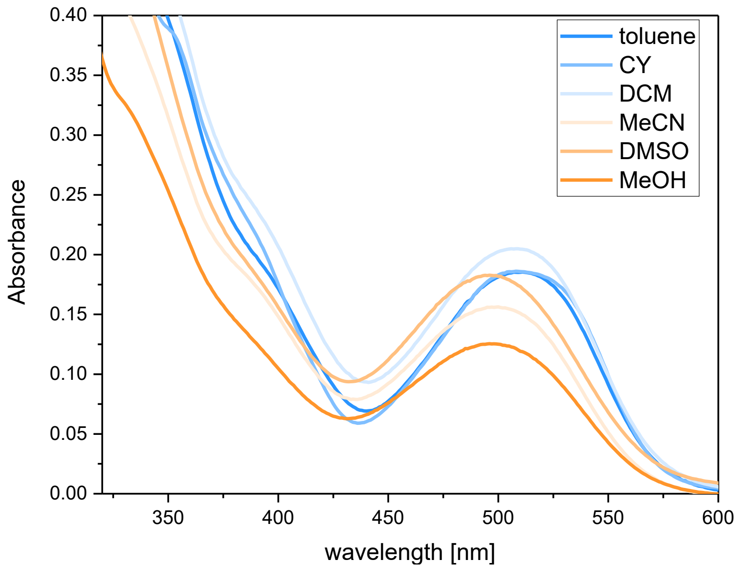

Figure S24. Limited solvatochromism of *E*-**PIO** (0.2 mM solutions).

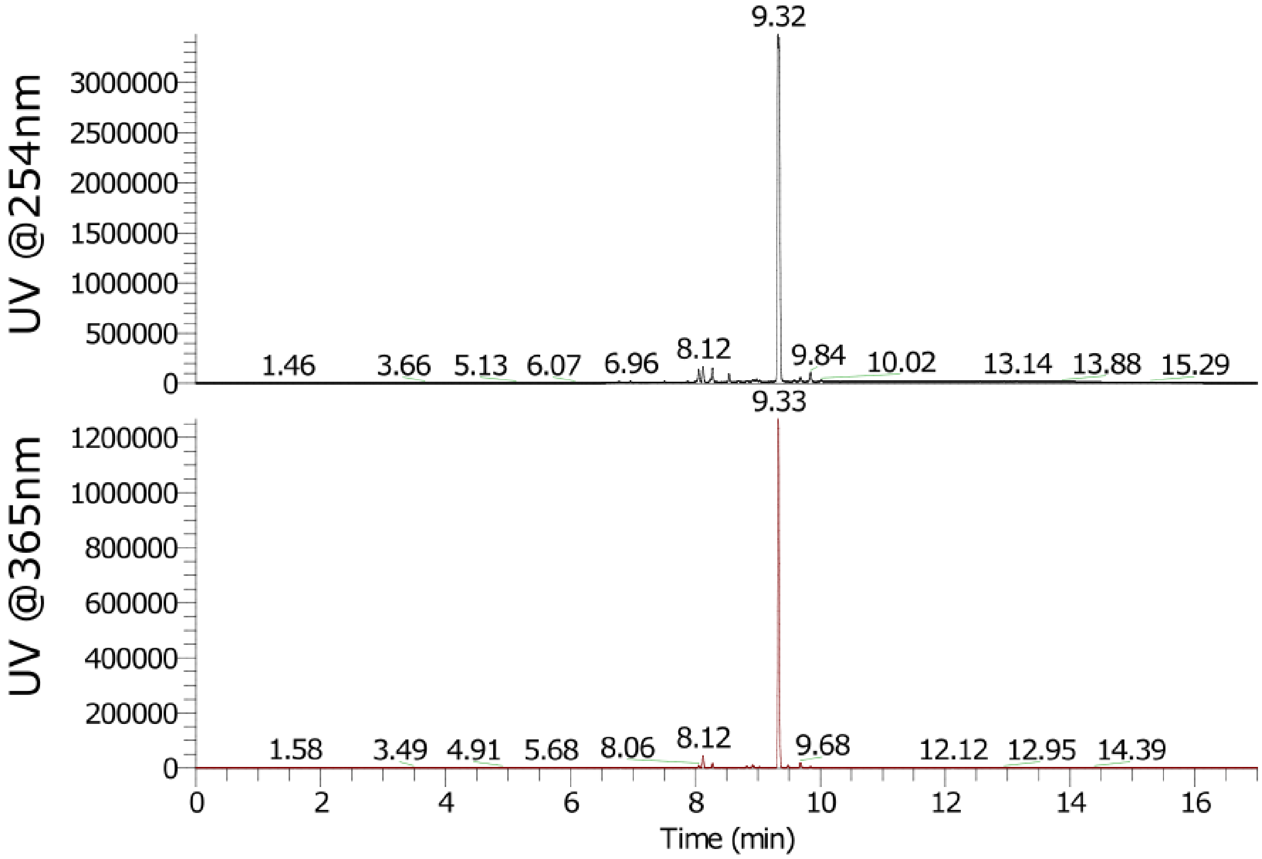

Figure S25. UPLC traces of *E*-**PIO**.

X-Ray structural data

E-PIO

Table S2. Crystal data and structure refinement for *E*-**PIO**.

Identification code	cu_NSredPIO_0ma_a
Empirical formula	$C_{16}H_{12}N_2O_2$
Formula weight	264.28
Temperature/K	100.0
Crystal system	monoclinic
Space group	$P2_1/c$
a/Å	16.6180(6)
b/Å	9.5463(3)
c/Å	7.9561(3)
α/°	90
β/°	101.335(2)
γ/°	90
Volume/Å^3	1237.54(8)
Z	4
ρ_{calc}g/cm^3	1.418
μ/mm^{-1}	0.776
F(000)	552.0
Crystal size/mm^3	0.209 × 0.154 × 0.074
Radiation	CuKα (λ = 1.54178)
2Θ range for data collection/°	5.424 to 136.664
Index ranges	-19 ≤ h ≤ 19, -11 ≤ k ≤ 11, -9 ≤ l ≤ 9
Reflections collected	32130
Independent reflections	2234 [R_{int} = 0.0675, R_{sigma} = 0.0259]
Data/restraints/parameters	2234/0/183
Goodness-of-fit on F^2	1.270
Final R indexes [I>=2σ (I)]	R_1 = 0.0517, wR_2 = 0.1208
Final R indexes [all data]	R_1 = 0.0593, wR_2 = 0.1248
Largest diff. peak/hole / e Å^{-3}	0.28/-0.19

Table S3. Fractional Atomic Coordinates (×10^4) and Equivalent Isotropic Displacement Parameters (Å^2×10^3) for *E*-**PIO**. U_{eq} is defined as 1/3 of of the trace of the orthogonalised U_{IJ} tensor.

Atom	*x*	*y*	*z*	U(eq)
O1	3421.6(10)	7638.1(18)	8240(2)	29.9(4)
O2	424.6(10)	6854(2)	3237(2)	37.5(5)
N1	1625.1(11)	6989(2)	5164(2)	25.3(5)
N2	2571.1(12)	5173(2)	6197(3)	25.9(5)
C9	3369.2(14)	4722(2)	7057(3)	22.8(5)
C10	4073.7(14)	5181(2)	6529(3)	23.4(5)
C11	4831.6(14)	4657(2)	7316(3)	26.2(5)
C6	2344.2(14)	8937(3)	6405(3)	24.3(5)
C7	2821.4(14)	7712(2)	7093(3)	24.1(5)
C8	2374.9(14)	6449(3)	6156(3)	23.0(5)
C12	4893.0(16)	3681(2)	8620(3)	29.7(6)
C1	1636.0(14)	8485(3)	5282(3)	24.9(5)
C5	2495.1(15)	10341(3)	6771(3)	27.7(6)
C14	3426.5(16)	3707(3)	8324(3)	29.2(6)
C2	1058.1(15)	9457(3)	4497(3)	30.5(6)
C15	965.5(14)	6228(3)	4207(3)	28.9(6)
C13	4190.7(17)	3212(3)	9120(3)	33.0(6)
C16	949.1(15)	4679(3)	4464(3)	31.9(6)
C4	1925.8(15)	11319(3)	5978(3)	31.1(6)
C3	1218.8(16)	10863(3)	4871(3)	32.1(6)

Table S4. Anisotropic Displacement Parameters (Å^2×10^3) for *E*-**PIO**. The Anisotropic displacement factor exponent takes the form: $-2\pi^2[h^2a^{*2}U_{11}+2hka^*b^*U_{12}+\ldots]$.

Atom	U_{11}	U_{22}	U_{33}	U_{23}	U_{13}	U_{12}
O1	27.0(9)	30.9(10)	27.7(9)	-6.7(7)	-4.8(7)	6.0(7)
O2	26.6(10)	46.5(12)	34.4(10)	-2.2(8)	-6.5(8)	2.2(8)
N1	19.9(10)	29.7(11)	23.8(10)	-3.1(8)	-1.7(8)	1.4(8)
N2	22.2(10)	27.3(11)	26.9(10)	-2.2(8)	2.0(8)	0.6(8)
C9	25.0(12)	20.7(11)	20.7(11)	-4.0(9)	-0.6(9)	0.7(9)
C10	28.2(12)	20.3(11)	21.4(11)	-0.4(9)	4.3(9)	1.4(9)
C11	23.7(12)	25.7(12)	28.1(12)	-5.6(10)	2.7(10)	1.9(9)
C6	21.2(12)	30.1(13)	21.8(11)	0.1(10)	4.6(9)	2.7(10)
C7	22.4(12)	29.1(13)	20.9(11)	-2.8(9)	4.0(9)	2.3(9)
C8	19.8(11)	30.4(13)	18.7(11)	-2.7(9)	3.3(9)	1.8(9)
C12	32.2(14)	23.4(13)	29.2(12)	-5.7(10)	-4.2(10)	8.0(10)
C1	21.9(12)	30.3(13)	22.3(11)	-0.1(10)	4.0(9)	2.8(10)
C5	25.1(12)	29.4(13)	28.1(12)	0.4(10)	3.5(10)	1.4(10)
C14	34.2(14)	27.2(13)	25.4(12)	-0.6(10)	4.1(10)	-6.1(10)
C2	24.6(13)	38.8(15)	26.6(12)	2.0(11)	1.5(10)	5.1(11)
C15	21.7(12)	39.9(15)	24.6(12)	-5.3(11)	3.2(10)	0.7(11)
C13	48.5(16)	21.4(12)	25.5(13)	2.7(10)	-1.3(11)	-0.3(11)
C16	23.6(13)	37.8(15)	32.8(13)	-6.8(11)	1.3(10)	-3.2(11)
C4	32.9(14)	27.8(13)	32.5(13)	3.1(11)	6.3(11)	3.8(11)
C3	29.0(13)	35.7(15)	31.4(13)	8.3(11)	5.7(11)	9.1(11)

Table S5. Bond Lengths for *E*-**PIO**.

Atom	Atom	Length/Å	Atom	Atom	Length/Å
O1	C7	1.215(3)	C6	C7	1.458(3)
O2	C15	1.220(3)	C6	C1	1.398(3)
N1	C8	1.434(3)	C6	C5	1.384(3)
N1	C1	1.431(3)	C7	C8	1.531(3)
N1	C15	1.407(3)	C12	C13	1.379(4)
N2	C9	1.434(3)	C1	C2	1.392(3)
N2	C8	1.260(3)	C5	C4	1.389(3)
C9	C10	1.390(3)	C14	C13	1.386(4)
C9	C14	1.389(3)	C2	C3	1.390(4)
C10	C11	1.385(3)	C15	C16	1.494(4)
C11	C12	1.383(4)	C4	C3	1.393(4)

Table S6. Bond Angles for *E*-**PIO**.

Atom	Atom	Atom	Angle/˚	Atom	Atom	Atom	Angle/˚
C1	N1	C8	108.95(18)	N2	C8	N1	123.7(2)
C15	N1	C8	127.8(2)	N2	C8	C7	130.6(2)
C15	N1	C1	123.3(2)	C13	C12	C11	119.5(2)
C8	N2	C9	120.9(2)	C6	C1	N1	110.3(2)
C10	C9	N2	121.1(2)	C2	C1	N1	129.6(2)
C14	C9	N2	118.8(2)	C2	C1	C6	120.1(2)
C14	C9	C10	119.8(2)	C6	C5	C4	118.4(2)
C11	C10	C9	119.7(2)	C13	C14	C9	119.7(2)
C12	C11	C10	120.6(2)	C3	C2	C1	117.5(2)
C1	C6	C7	108.7(2)	O2	C15	N1	119.2(2)
C5	C6	C7	129.4(2)	O2	C15	C16	122.7(2)
C5	C6	C1	121.9(2)	N1	C15	C16	118.0(2)
O1	C7	C6	129.4(2)	C12	C13	C14	120.6(2)
O1	C7	C8	124.6(2)	C5	C4	C3	119.5(2)
C6	C7	C8	105.86(19)	C2	C3	C4	122.7(2)
N1	C8	C7	105.68(19)				

Table S7. Hydrogen Atom Coordinates (Å×10^4) and Isotropic Displacement Parameters (Å^2×10^3) for *E*-**PIO**.

Atom	*x*	*y*	*z*	U(eq)
H10	4035.55	5850.5	5634.06	28
H11	5313.42	4969.97	6956.15	31
H12	5415.11	3335.86	9167.61	36
H5	2976.56	10628.91	7546.73	33
H14	2944.02	3352.39	8644.65	35
H2	572.3	9169.49	3734.23	37
H13	4231.3	2543.49	10017.28	40
H16A	434.63	4296.64	3809.55	48
H16B	988.13	4474.55	5684.64	48
H16C	1413.87	4249.44	4066.99	48
H4	2018.18	12290.8	6190.07	37
H3	830.43	11541.47	4349.76	38

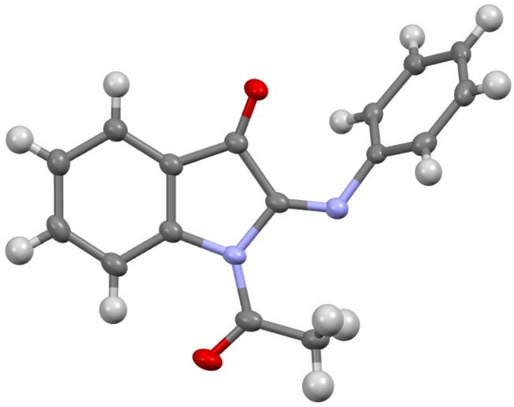

Figure S26. X-Ray structure of *E*-**PIO** (50% probability ellipsoids; explicit hydrogens are included).

Z-*ITI*

Table S8. Crystal data and structure refinement for *Z*-**ITI**.

Identification code	cu_NSyellowITI_0m_a
Empirical formula	$C_{14}H_9NOS$
Formula weight	239.28
Temperature/K	100.0
Crystal system	monoclinic
Space group	$P2_1/n$
a/Å	6.7568(2)
b/Å	23.9148(8)
c/Å	7.1253(2)
α/°	90
β/°	103.8330(10)
γ/°	90
Volume/Å^3	1117.97(6)
Z	4
ρ_{calc}g/cm^3	1.422
μ/mm^{-1}	2.401
F(000)	496.0
Crystal size/mm^3	0.17 × 0.156 × 0.078
Radiation	CuKα (λ = 1.54178)
2Θ range for data collection/°	13.322 to 136.416
Index ranges	-8 ≤ h ≤ 8, -28 ≤ k ≤ 28, -8 ≤ l ≤ 8
Reflections collected	19902
Independent reflections	2030 [R_{int} = 0.1137, R_{sigma} = 0.0426]
Data/restraints/parameters	2030/0/154
Goodness-of-fit on F^2	1.043
Final R indexes [I>=2σ (I)]	R_1 = 0.0328, wR_2 = 0.0828
Final R indexes [all data]	R_1 = 0.0336, wR_2 = 0.0836
Largest diff. peak/hole / e Å^{-3}	0.39/-0.36

Table S9. Fractional Atomic Coordinates (×10^4) and Equivalent Isotropic Displacement Parameters (Å^2×10^3) for *Z*-**ITI**. U_{eq} is defined as 1/3 of of the trace of the orthogonalised U_{IJ} tensor.

Atom	*x*	*y*	*z*	U(eq)
S1	7863.2(5)	5328.1(2)	3227.1(5)	13.57(14)
O1	2201.3(15)	5693.1(5)	934.3(15)	18.6(3)
N1	5765.7(18)	6326.6(5)	2376.6(16)	13.0(3)
C7	3794(2)	5470.7(6)	1721(2)	13.3(3)
C8	5788(2)	5795.5(6)	2391.1(19)	12.3(3)
C9	7541(2)	6655.7(6)	2949.9(19)	12.5(3)
C1	4147(2)	4875.1(6)	2164.1(19)	14.0(3)
C14	9370(2)	6523.7(6)	2463(2)	13.5(3)
C6	6192(2)	4748.2(6)	2970(2)	13.8(3)
C13	11029(2)	6881.3(6)	2982(2)	15.9(3)
C10	7387(2)	7155.4(6)	3928(2)	16.0(3)
C3	3258(2)	3912.7(7)	2365(2)	19.6(3)
C12	10885(2)	7369.4(6)	4001(2)	18.3(3)
C2	2665(2)	4458.5(7)	1859(2)	17.0(3)
C5	6796(2)	4202.0(6)	3468(2)	18.5(3)
C11	9057(2)	7505.9(7)	4466(2)	18.6(3)
C4	5306(3)	3788.1(6)	3154(2)	20.7(3)

Table S10. Anisotropic Displacement Parameters (Å^2×10^3) for *Z*-**ITI**. The Anisotropic displacement factor exponent takes the form: $-2\pi^2[h^2a^{*2}U_{11}+2hka^*b^*U_{12}+\ldots]$.

Atom	U_{11}	U_{22}	U_{33}	U_{23}	U_{13}	U_{12}
S1	11.8(2)	11.8(2)	16.5(2)	0.81(12)	2.21(14)	0.38(12)
O1	12.5(5)	18.8(5)	24.2(5)	-4.9(4)	4.2(4)	1.8(4)
N1	12.7(6)	14.2(6)	13.5(6)	-1.2(4)	6.0(5)	0.5(5)
C7	13.5(7)	15.9(7)	12.7(7)	-4.5(5)	7.4(5)	-1.0(6)
C8	12.6(7)	15.0(7)	10.9(6)	-0.4(5)	5.9(5)	1.0(6)
C9	13.0(7)	12.1(7)	12.5(6)	2.4(5)	3.2(5)	0.1(5)
C1	17.9(7)	15.9(7)	10.2(6)	-2.4(5)	7.1(5)	-1.3(6)
C14	15.8(7)	11.5(7)	14.3(7)	1.5(5)	5.7(5)	1.8(5)
C6	17.5(7)	14.2(7)	10.2(6)	-1.7(5)	4.7(5)	-2.2(6)
C13	13.6(7)	17.6(7)	18.2(7)	2.7(6)	7.0(6)	1.0(6)
C10	16.3(7)	15.1(7)	18.1(7)	-0.6(6)	7.4(6)	1.4(6)
C3	27.4(8)	16.9(8)	15.3(7)	-2.6(6)	6.9(6)	-8.9(6)
C12	16.9(7)	17.0(8)	21.3(7)	0.1(6)	5.0(6)	-4.5(6)
C2	18.4(7)	20.4(8)	13.9(7)	-3.5(6)	7.2(6)	-4.6(6)
C5	22.4(8)	16.6(7)	15.3(7)	1.5(6)	2.3(6)	1.1(6)
C11	22.1(8)	14.9(7)	19.9(7)	-3.0(6)	7.2(6)	-0.8(6)
C4	32.4(9)	13.2(7)	15.6(7)	0.9(6)	4.3(6)	-2.9(7)

Table S11. Bond Lengths for *Z*-**ITI**.

Atom	Atom	Length/Å	Atom	Atom	Length/Å
S1	C8	1.7809(14)	C1	C2	1.392(2)
S1	C6	1.7702(15)	C14	C13	1.388(2)
O1	C7	1.2110(18)	C6	C5	1.389(2)
N1	C8	1.270(2)	C13	C12	1.390(2)
N1	C9	1.4112(19)	C10	C11	1.384(2)
C7	C8	1.5294(19)	C3	C2	1.387(2)
C7	C1	1.466(2)	C3	C4	1.396(2)
C9	C14	1.397(2)	C12	C11	1.392(2)
C9	C10	1.400(2)	C5	C4	1.391(2)
C1	C6	1.397(2)			

Table S12. Bond Angles for *Z*-**ITI**.

Atom	Atom	Atom	Angle/°	Atom	Atom	Atom	Angle/°
C6	S1	C8	91.45(7)	C2	C1	C6	120.83(14)
C8	N1	C9	123.22(12)	C13	C14	C9	120.03(13)
O1	C7	C8	122.71(13)	C1	C6	S1	114.60(11)
O1	C7	C1	127.39(14)	C5	C6	S1	124.67(12)
C1	C7	C8	109.90(12)	C5	C6	C1	120.72(14)
N1	C8	S1	129.58(11)	C14	C13	C12	120.26(13)
N1	C8	C7	119.84(13)	C11	C10	C9	120.14(14)
C7	C8	S1	110.49(10)	C2	C3	C4	120.19(14)
C14	C9	N1	122.91(13)	C13	C12	C11	119.85(14)
C14	C9	C10	119.47(13)	C3	C2	C1	118.69(15)
C10	C9	N1	117.45(13)	C6	C5	C4	118.07(14)
C6	C1	C7	113.18(13)	C10	C11	C12	120.21(14)
C2	C1	C7	125.99(14)	C5	C4	C3	121.49(15)

Table S13. Hydrogen Atom Coordinates (Å×10^4) and Isotropic Displacement Parameters (Å^2×10^3) for *Z*-**ITI**.

Atom	*x*	*y*	*z*	U(eq)
H14	9478.43	6188.97	1776.51	16
H13	12267.22	6792.15	2640.25	19
H10	6133.79	7254.25	4222.9	19
H3	2267.98	3623.12	2174.07	23
H12	12032.22	7609.28	4379.29	22
H2	1274.49	4546.16	1314.93	20
H5	8186.78	4113.66	4007.7	22
H11	8954.99	7840.65	5154.11	22
H4	5691.81	3412.04	3483.86	25

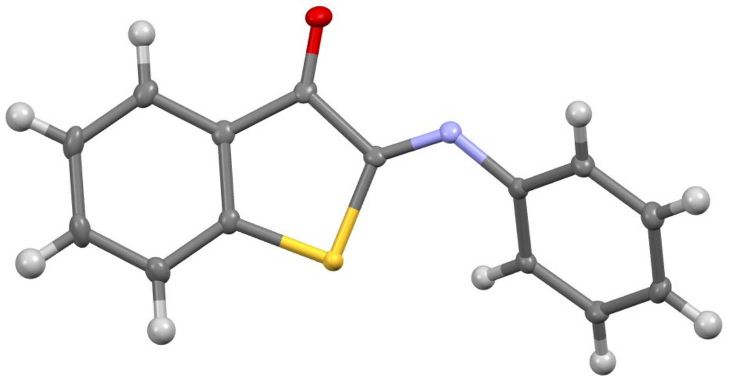

Figure S27. X-Ray structure of Z-**ITI** (50% probability ellipsoids; explicit hydrogens are included).

Femtosecond Transient Absorption Spectroscopy

We have recorded transient absorption spectra of *E*-**PIO** dissolved in toluene upon excitation at 500 nm. The data have been processed using global analysis, applying a linear decay kinetic scheme with three time constants. As can be noticed by observing the EADS in Figure S28, we observe directly after excitation an intense and broad excited state absorption band extending over the full probed spectral window and with maximum intensity at about 525 nm. The intensity of this band is greatly reduced in about 1.4 ps. The second EADS (red line) shows a dip in the 470-530 nm region and two positive contributions on both the red and blue side of the dip. The positive signal peaked at about 550 nm completely decays in the following 4.8 ps. The final spectral component has a differential line shape with a negative contribution in the 470-550 nm region where the ground state absorption of the initial isomer is observed, and a positive product band at shorter wavelengths.

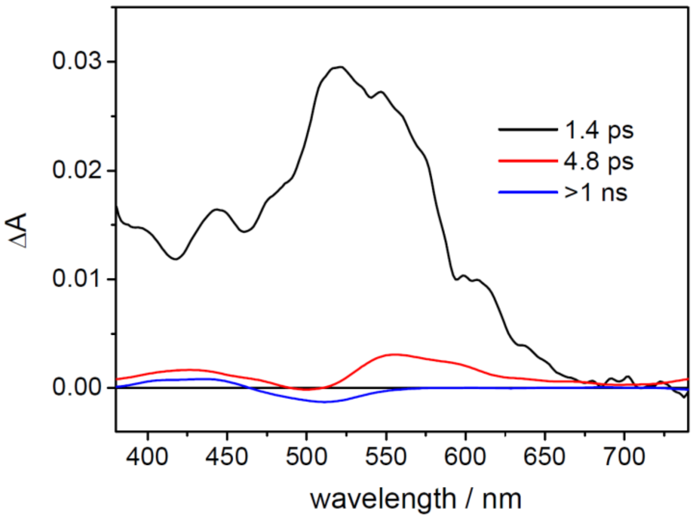

Figure S28. EADS obtained from global analysis of transient absorption data recorded for **PIO** in toluene upon excitation at 510 nm.

Analogous to what we observed in case of ITI,[12] we interpret the initial evolution occurring in 1.4 ps as being associated with reaching the conical intersection between the ground and electronically excited state of the molecule, from which it decays to the ground state of both the *Z* and *E* isomers. In the following spectral component, the positive band peaked at about 550 nm is associated with absorption from a hot ground state of the *E* isomer, while the one peaked at 425 nm with the absorption of the hot ground state of the *Z* isomer. The final spectral component reflects the differential absorption between the bleaching of the *E* isomer and the ground state absorption of the *Z* isomer product. Kinetic traces recorded near the maxima of the absorption bands of the two isomers reported in Figure S29 confirm the fast decay of the broad excited state band and the persistence of small, long-living positive and negative contributions.

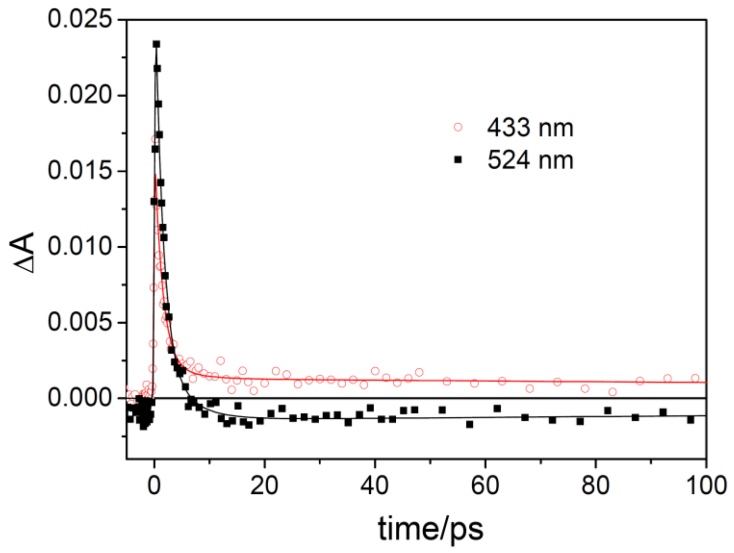

Figure S29. Kinetic traces recorded for PIO in toluene upon excitation at 510 nm.

Transient absorption measurements have been repeated in solvents with different polarities: cyclohexane, dichloromethane, methanol, DMSO, acetonitrile and diethyl ether. The overall excited state behavior of PIO in all solvents is similar to that observed in toluene although in polar solvents the kinetics of the excited state decay are accelerated compared to those in non-polar ones. The evolution of the transient spectra is instead qualitatively similar in all solvents. As a comparison to what is observed in toluene, Figure S30 reports the EADS obtained from a global analysis of PIO dissolved in methanol. In Figure S31 a comparison of the kinetic traces obtained on the maximum of the excited state absorption band in toluene and methanol is reported. The kinetic constants extracted from the global analyses in the various solvents are reported in Table S14.

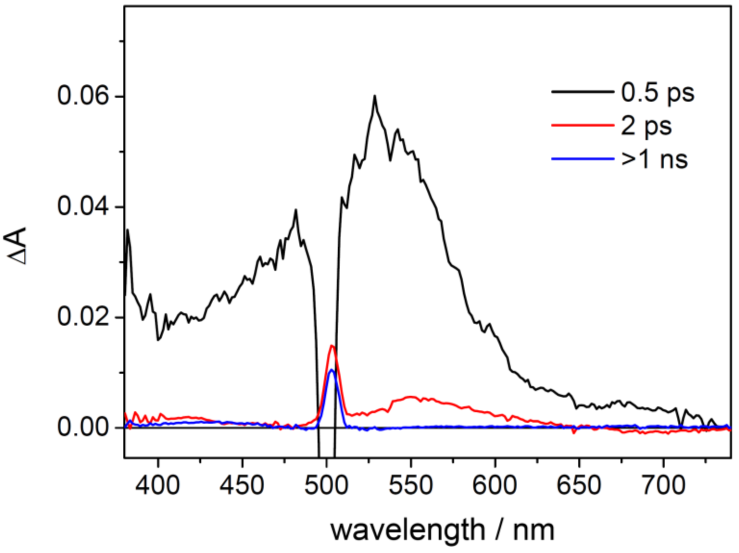

Figure S30. EADS obtained from global analysis of transient absorption data recorded for PIO in toluene upon excitation at 500 nm (scattered pump light is visible at the excitation wavelength).

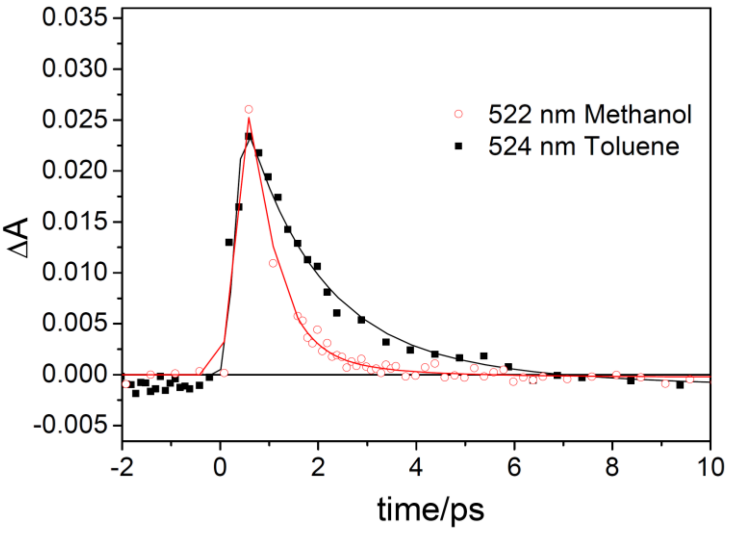

Figure S31. Comparison of the kinetic traces registered in toluene and methanol at the maximum of the excited state absorption band (zoom on the short timescale).

Table S14. Lifetimes extracted from global analysis in the solvents used for transient absorption measurements.

Solvent	τ_1 [ps]	τ_2 [ps]	τ_3 [ns]
Toluene	1.4	4.8	>1
Cyclohexane	1.2	3.8	>1
DCM	0.7	11.5	>1
Methanol	0.5	2.0	>1
DMSO	0.6	7.0	>1
MeCN	0.6	8.2	>1
Et_2O	0.8	6.3	>1

Nanosecond Transient Absorption Spectroscopy

An estimate of the quantum yield for photoisomerization of the **PIO** derivatives following excitation with 2.6 mJ laser pulses was obtained using the Lambert-Beer law by calculating the number of excited molecules and the observed bleach upon irradiation corrected for the probability of absorption (see Table S15). Herein it was assumed that the *Z*-isomer does not absorb in the 510 nm region. The very small absorption at 510 nm observed in the spectra of the *Z* isomer would lead to an increase of the reported quantum yields.

Table S15. Quantum yield and photophysical properties measured at 20 °C.

	λ_{max} [nm]	ε/1000 [$M^{-1}cm^{-1}$]	OD in 1 cm cell	Maximum bleach possible [ΔOD]	Maximum bleach observed [ΔOD]	Φ_{EZ} [%]	τ_{ZE} [µs]
**PIO** (Cyclohexane)	508	0.93	1.04	-0.364	-0.0250	9.0	27
**PIO** (Toluene)	510	0.93	1.10	-0.293	-0.0195	6.7	77
**PIO** (DCM)	509	1.0	1.26	-0.362	-0.0250	6.9	74
**PIO** (MeCN)	498	0.76	1.15	-0.249	-0.0140	5.6	76
**PIO** (MeOH)	500	0.59	1.30	-0.215	-0.0250	11.6	111
**PIO** (DMSO)	496	0.86	1.18	-0.289	-0.0140	4.8	67
**PIO-NO_2** (MeCN)	473	1.2	0.99	-0.291	-0.0040	1.4	1.8
**PIO-CN** (MeCN)	470	1.6	0.69	-0.291	-0.0025	0.9	3.7
**PIO-COOMe** (MeCN)	489	0.94	0.71	-0.174	-0.0042	2.4	7.2
**PIO-Br** (MeCN)	501	1.2	0.84	-0.262	-0.0025	1.0	54
**PIO-Me** (MeCN)	510	1.1	1.09	-0.322	-0.0068	2.1	206
**PIO-OMe** (MeCN)	525	2.0	1.11	-0.605	-0.0050	0.8	678
**iBu-PIO** (MeCN)	505	0.92	1.11	-0.269	-0.0078	2.9	164
**PIO-NO_2** (MeOH)	474	1.2	0.94	-0.280	-0.0128	4.6	1.5
**PIO-CN** (MeOH)	468	1.6	0.69	-0.296	-0.0016	0.5	2.6
**PIO-COOMe** (MeOH)	488	0.94	0.71	-0.174	-0.0025	1.4	6.4
**PIO-Br** (MeOH)	498	1.2	0.84	-0.271	-0.0078	2.9	69
**PIO-Me** (MeOH)	513	1.3	1.09	-0.365	-0.0060	1.6	283
**PIO-OMe** (MeOH)	522	2.0	0.99	-0.563	-0.0117	2.1	805
**iBu-PIO** (MeOH)	501	1.0	1.30	-0.323	-0.0180	5.6	269

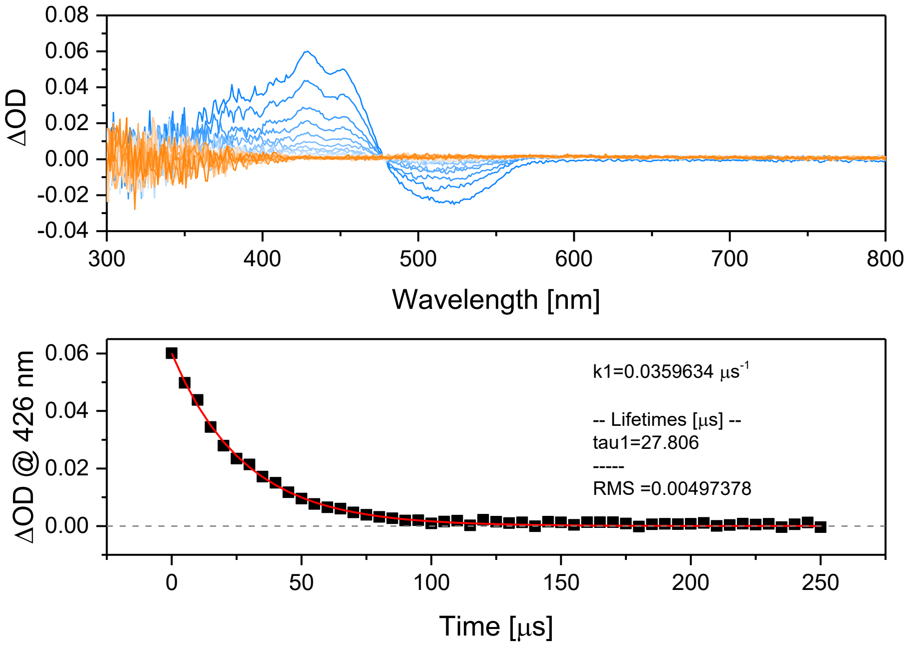

Figure S32. Top: Ns transient absorption of 1 mM *E*-**PIO** in cyclohexane at room temperature. The sample was irradiated at 510 nm upon which the spectrum was recorded in steps of 10 µs increasing delay. Bottom: Fit of the decay of the absorption maximum of the transient signal (red line) as obtained from a global analysis of the transient absorption spectra in the top panel.

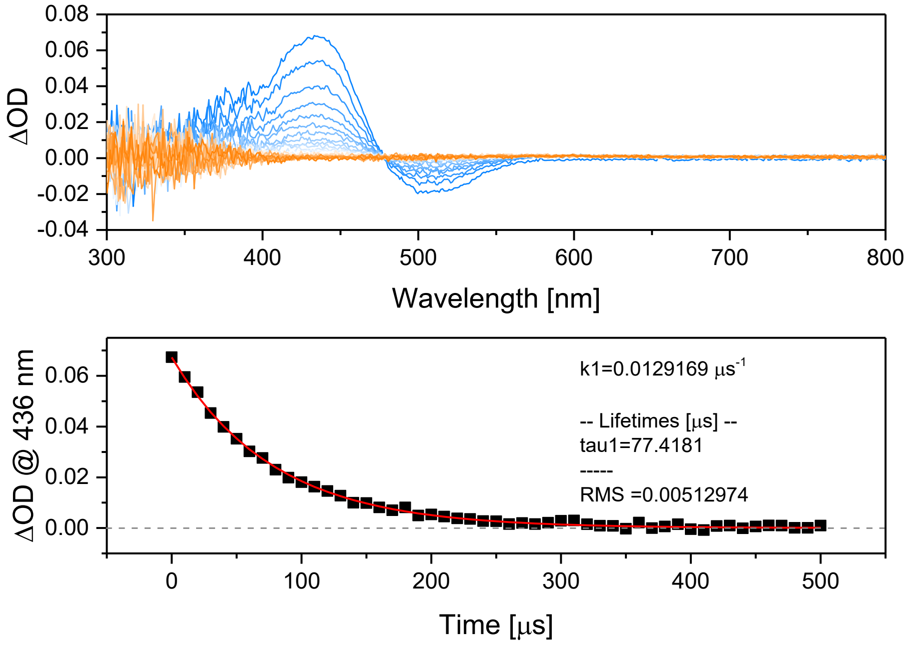

Figure S33. Top: Ns transient absorption of 1 mM *E*-**PIO** in toluene at room temperature. The sample was irradiated at 510 nm upon which the spectrum was recorded in steps of 20 µs increasing delay. Bottom: Fit of the decay of the absorption maximum of the transient signal (red line) as obtained from a global analysis of the transient absorption spectra in the top panel.

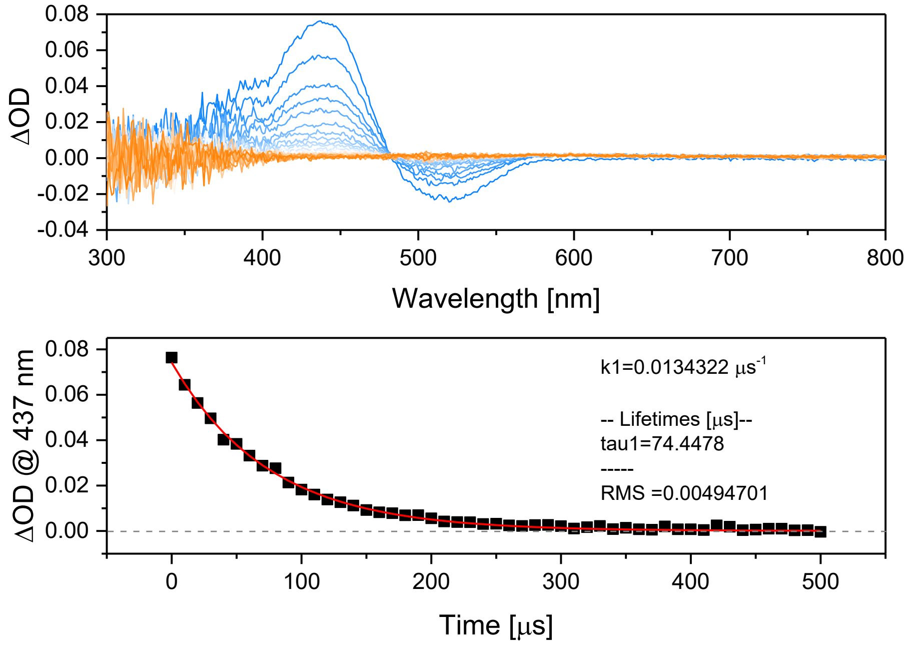

Figure S34. Top: Ns transient absorption of 1 mM *E*-**PIO** in DCM at room temperature. The sample was irradiated at 510 nm upon which the spectrum was recorded in steps of 20 µs increasing delay. Bottom: Fit of the decay of the absorption maximum of the transient signal (red line) as obtained from a global analysis of the transient absorption spectra in the top panel.

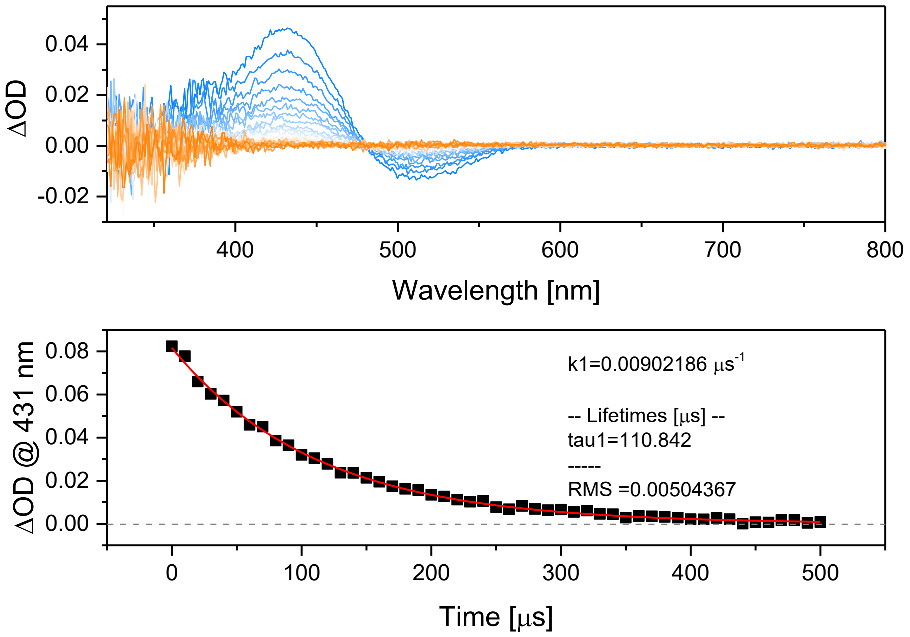

Figure S35. Top: Ns transient absorption of 1 mM *E*-**PIO** in MeOH at room temperature. The sample was irradiated at 510 nm upon which the spectrum was recorded in steps of 20 µs increasing delay. Bottom: Fit of the decay of the absorption maximum of the transient signal (red line) as obtained from a global analysis of the transient absorption spectra in the top panel.

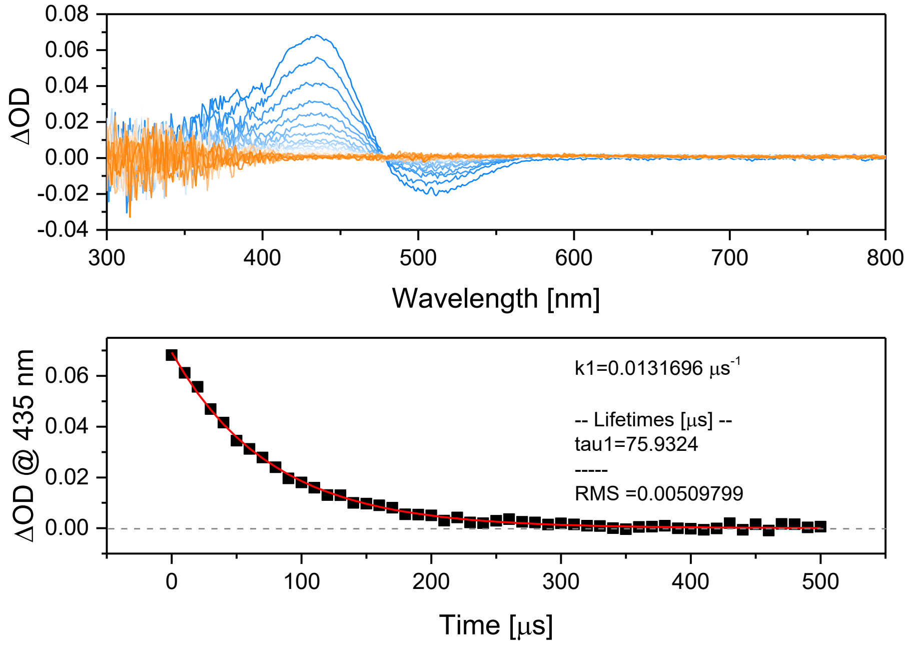

Figure S36. Top: Ns transient absorption of 1 mM *E*-**PIO** in MeCN at room temperature. The sample was irradiated at 510 nm upon which the spectrum was recorded in steps of 20 µs increasing delay. Bottom: Fit of the decay of the absorption maximum of the transient signal (red line) as obtained from a global analysis of the transient absorption spectra in the top panel.

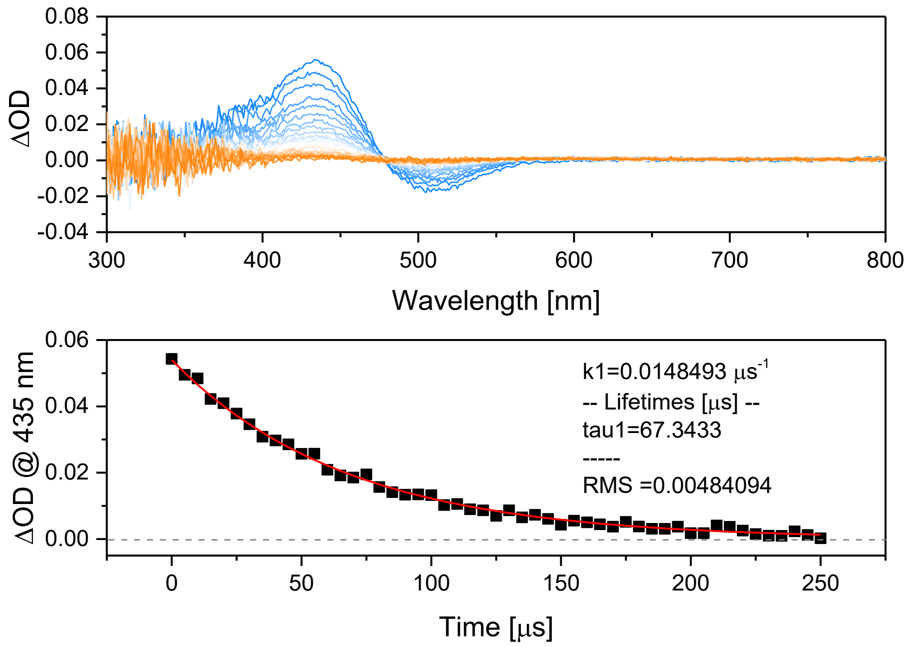

Figure S37. Top: Ns transient absorption of 1 mM *E*-**PIO** in DMSO at room temperature. The sample was irradiated at 510 nm upon which the spectrum was recorded in steps of 20 µs increasing delay. Bottom: Fit of the decay of the absorption maximum of the transient signal (red line) as obtained from a global analysis of the transient absorption spectra in the top panel.

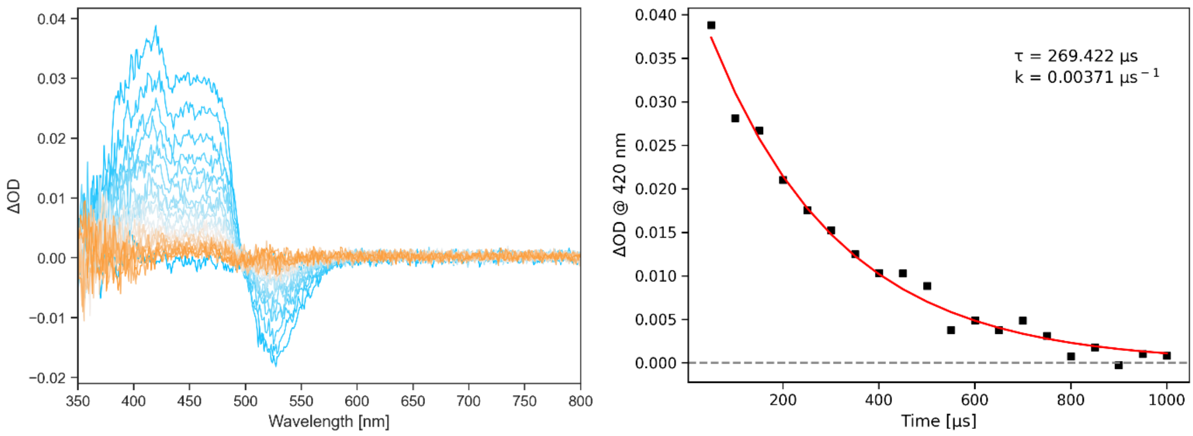

Figure S38. Left: Ns transient absorption of 1 mM *E*-**iBu-PIO** in MeOH at room temperature. The sample was irradiated at 505 nm upon which the spectrum was recorded in steps of 50 µs increasing delay. Right: Fit of the decay of the absorption maximum of the transient signal (red line) as obtained from a global analysis of the transient absorption spectra in the left panel.

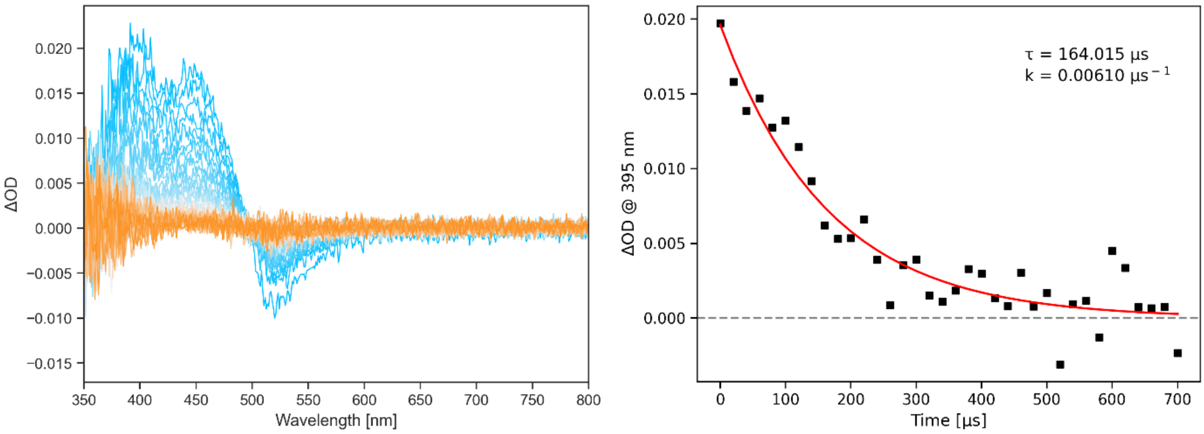

Figure S39. Left: Ns transient absorption of 1 mM *E*-**iBu-PIO** in MeCN at room temperature. The sample was irradiated at 505 nm upon which the spectrum was recorded in steps of 20 μs increasing delay. Right: Fit of the decay of the absorption maximum of the transient signal (red line) as obtained from a global analysis of the transient absorption spectra in the left panel.

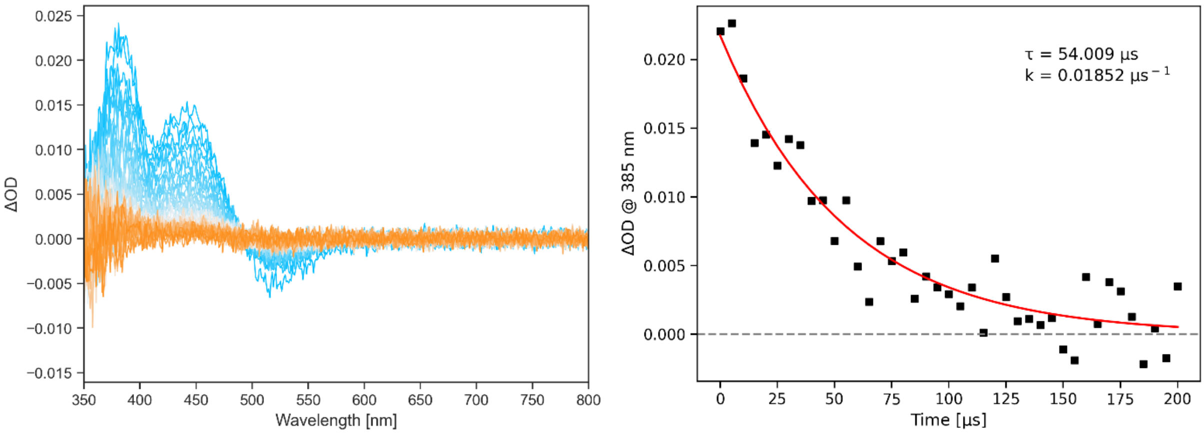

Figure S40. Left: Ns transient absorption of 1 mM *E*-**PIO-Br** in MeCN at room temperature. The sample was irradiated at 505 nm upon which the spectrum was recorded in steps of 5 μs increasing delay. Right: Fit of the decay of the absorption maximum of the transient signal (red line) as obtained from a global analysis of the transient absorption spectra in the left panel.

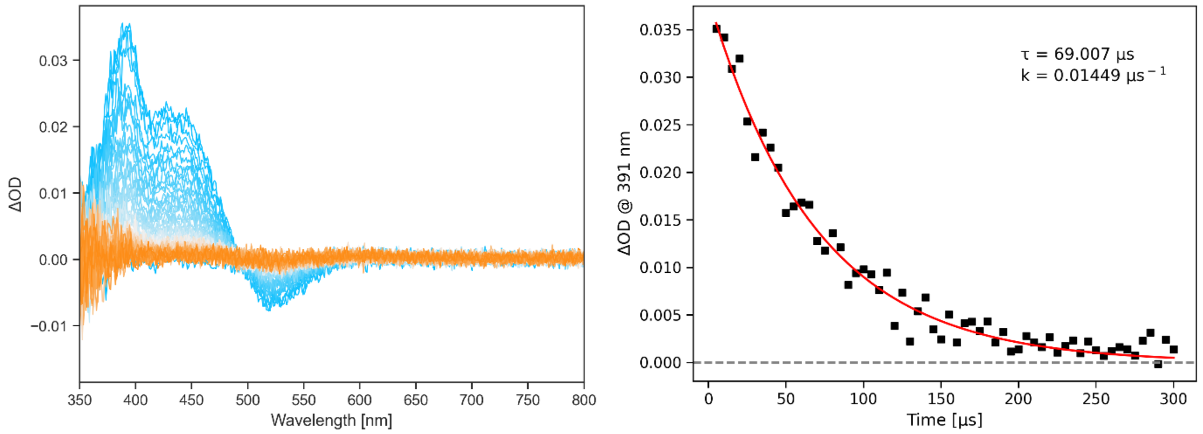

Figure S41. Left: Ns transient absorption of 1 mM *E*-**PIO-Br** in MeOH at room temperature. The sample was irradiated at 505 nm upon which the spectrum was recorded in steps of 10 μs increasing delay. Right: Fit of the decay of the absorption maximum of the transient signal (red line) as obtained from a global analysis of the transient absorption spectra in the left panel.

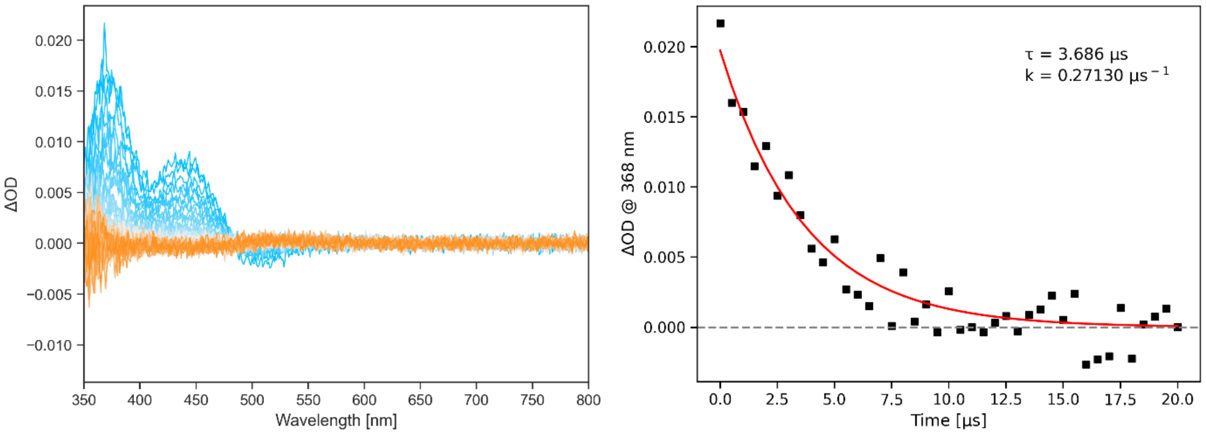

Figure S42. Left: Ns transient absorption of 1 mM *E*-**PIO-CN** in MeCN at room temperature. The sample was irradiated at 480 nm upon which the spectrum was recorded in steps of 0.5 μs increasing delay. Right: Fit of the decay of the absorption maximum of the transient signal (red line) as obtained from a global analysis of the transient absorption spectra in the left panel.

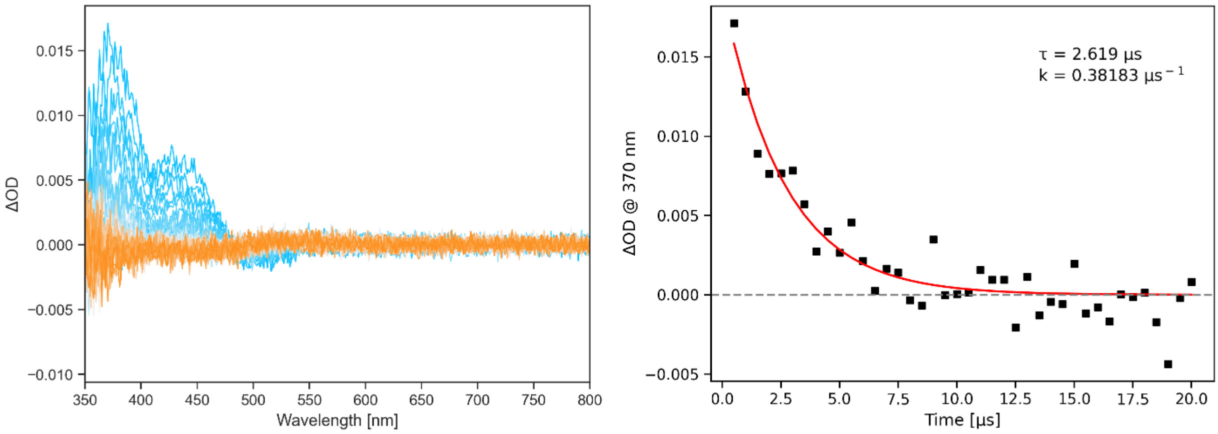

Figure S43. Left: Ns transient absorption of 1 mM *E*-**PIO-CN** in MeOH at room temperature. The sample was irradiated at 480 nm upon which the spectrum was recorded in steps of 0.5 μs increasing delay. Right: Fit of the decay of the absorption maximum of the transient signal (red line) as obtained from a global analysis of the transient absorption spectra in the left panel.

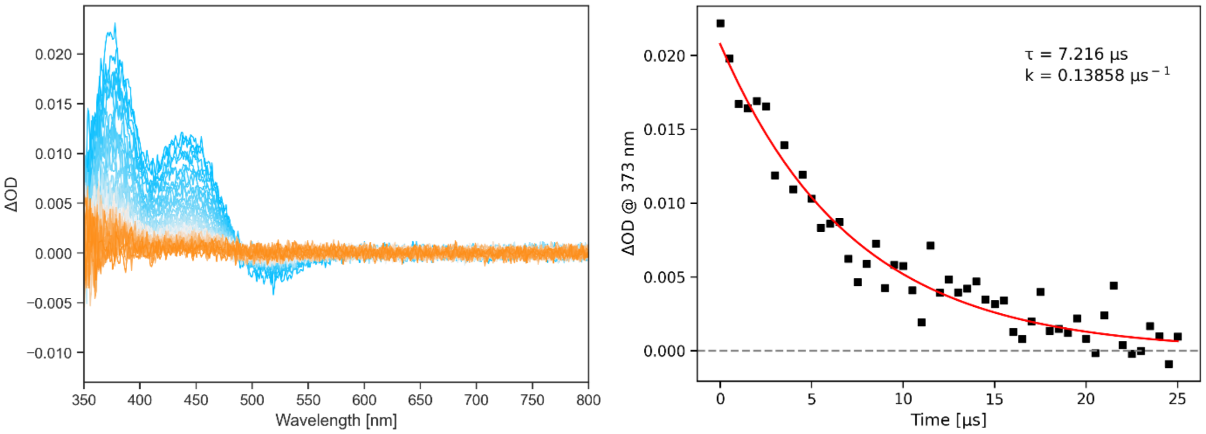

Figure S44. Left: Ns transient absorption of 1 mM *E*-**PIO-COOMe** in MeCN at room temperature. The sample was irradiated at 480 nm upon which the spectrum was recorded in steps of 1.0 μs increasing delay. Right: Fit of the decay of the absorption maximum of the transient signal (red line) as obtained from a global analysis of the transient absorption spectra in the left panel.

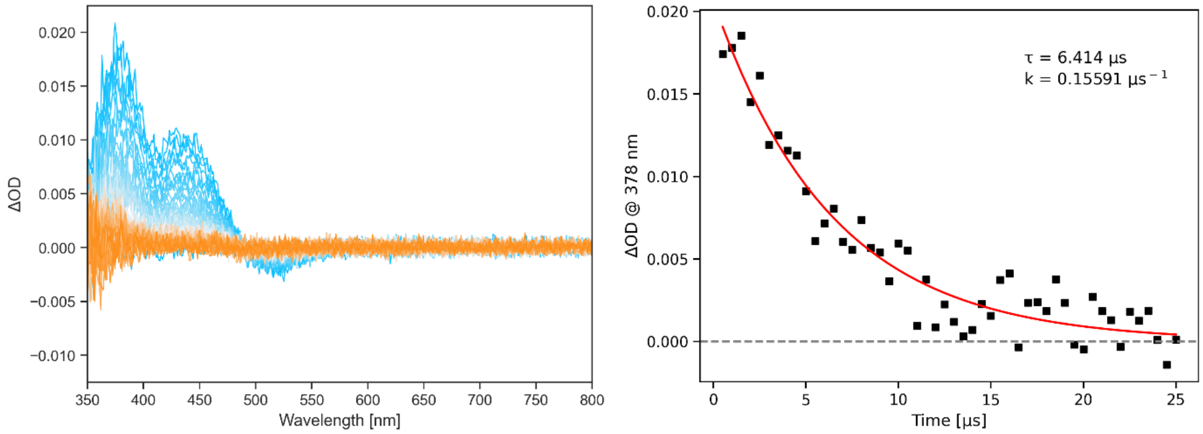

Figure S45. Left: Ns transient absorption of 1 mM *E*-**PIO-COOMe** in MeOH at room temperature. The sample was irradiated at 480 nm upon which the spectrum was recorded in steps of 1.0 μs increasing delay. Right: Fit of the decay of the absorption maximum of the transient signal (red line) as obtained from a global analysis of the transient absorption spectra in the left panel.

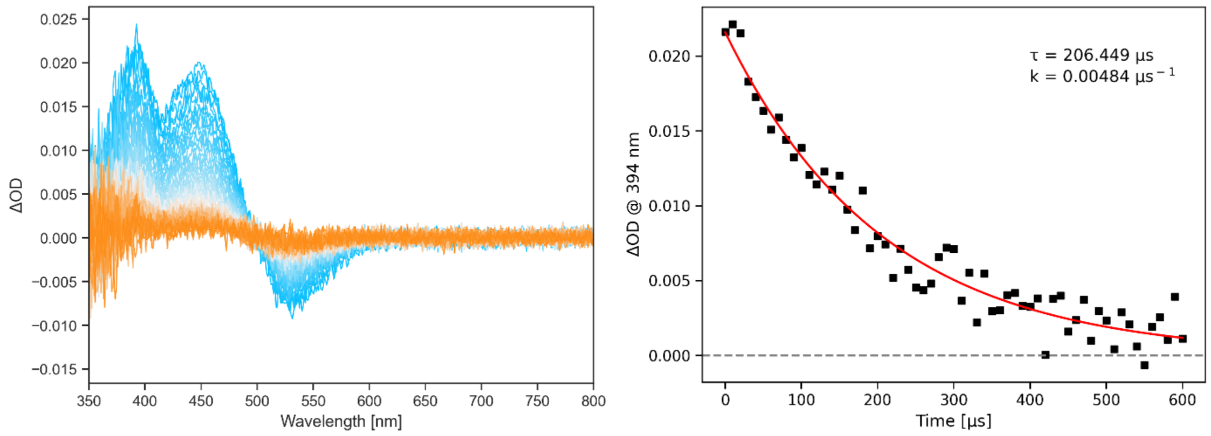

Figure S46. Left: Ns transient absorption of 1 mM *E*-**PIO-Me** in MeCN at room temperature. The sample was irradiated at 505 nm upon which the spectrum was recorded in steps of 10 μs increasing delay. Right: Fit of the decay of the absorption maximum of the transient signal (red line) as obtained from a global analysis of the transient absorption spectra in the left panel.

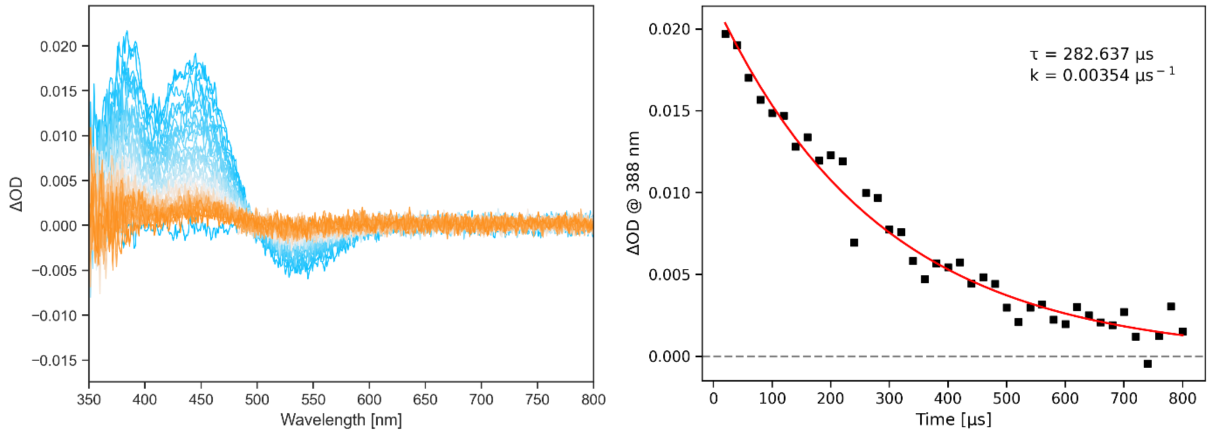

Figure S47. Left: Ns transient absorption of 1 mM *E*-**PIO-Me** in MeOH at room temperature. The sample was irradiated at 505 nm upon which the spectrum was recorded in steps of 10 μs increasing delay. Right: Fit of the decay of the absorption maximum of the transient signal (red line) as obtained from a global analysis of the transient absorption spectra in the left panel.

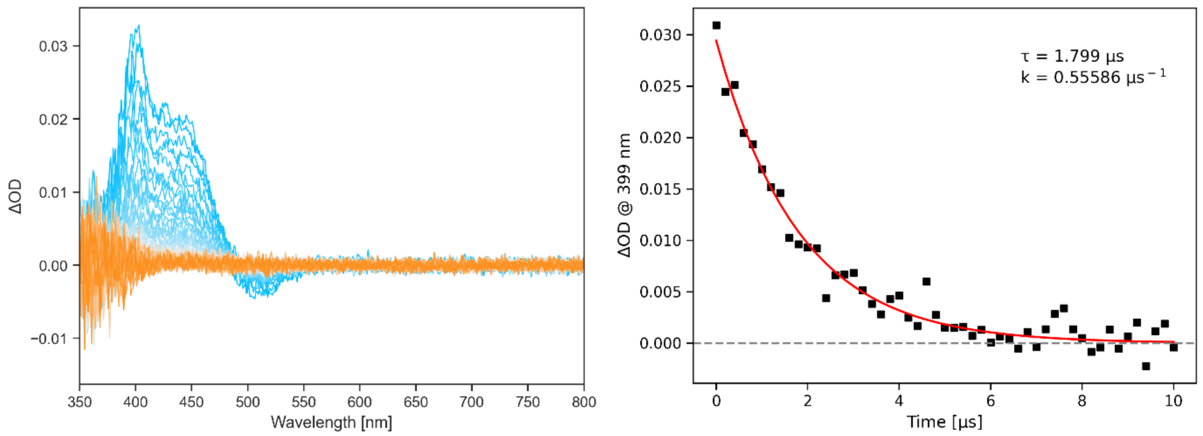

Figure S48. Left: Ns transient absorption of 1 mM *E*-**PIO-NO$_2$** in MeCN at room temperature. The sample was irradiated at 480 nm upon which the spectrum was recorded in steps of 0.25 μs increasing delay. Right: Fit of the decay of the absorption maximum of the transient signal (red line) as obtained from a global analysis of the transient absorption spectra in the left panel.

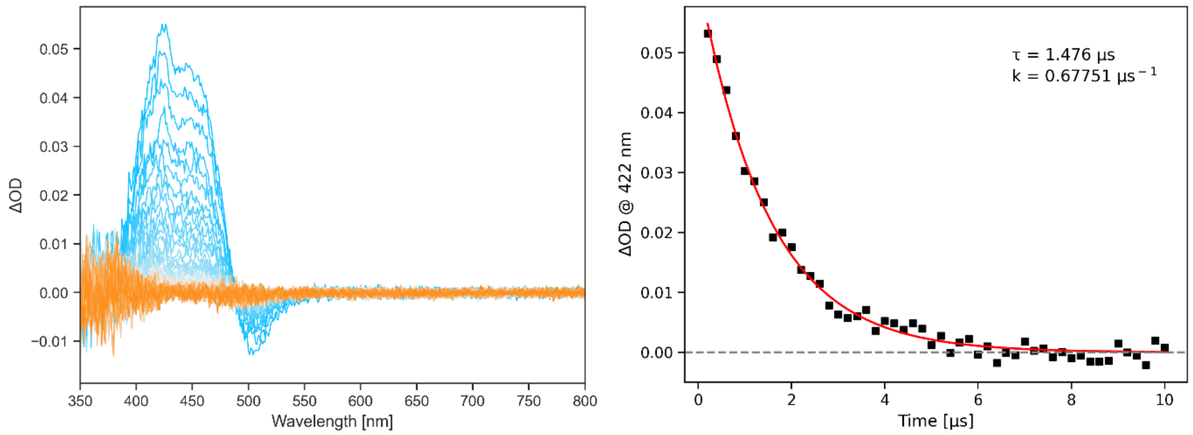

Figure S49. Left: Ns transient absorption of 1 mM *E*-**PIO-NO$_2$** in MeOH at room temperature. The sample was irradiated at 480 nm upon which the spectrum was recorded in steps of 0.25 μs increasing delay. Right: Fit of the decay of the absorption maximum of the transient signal (red line) as obtained from a global analysis of the transient absorption spectra in the left panel.

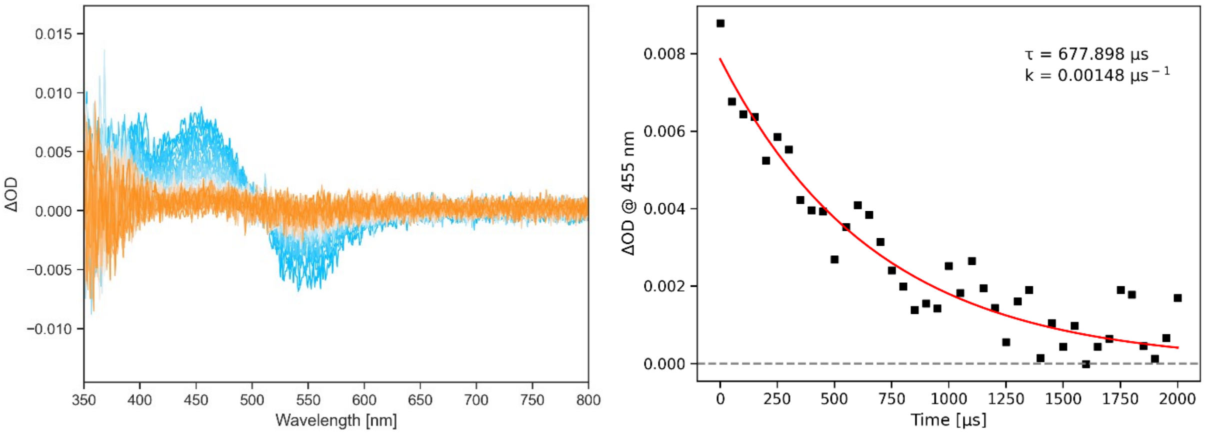

Figure S50. Left: Ns transient absorption of 1 mM *E*-**PIO-OMe** in MeCN at room temperature. The sample was irradiated at 480 nm upon which the spectrum was recorded in steps of 50 μs increasing delay. Right: Fit of the decay of the absorption maximum of the transient signal (red line) as obtained from a global analysis of the transient absorption spectra in the left panel.

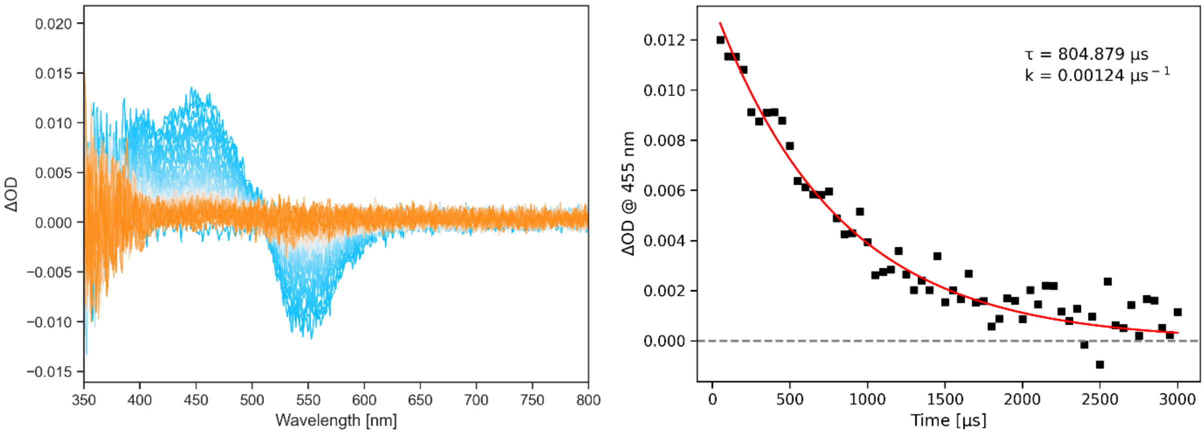

Figure S51. Left: Ns transient absorption of 1 mM *E*-**PIO-OMe** in MeOH at room temperature. The sample was irradiated at 480 nm upon which the spectrum was recorded in steps of 50 µs increasing delay. Right: Fit of the decay of the absorption maximum of the transient signal (red line) as obtained from a global analysis of the transient absorption spectra in the left panel.

Low-temperature NMR, UV-Vis and Activation Parameter Analysis

Low temperature NMR

A sample of *E*-**PIO** was dissolved in Et_2O-*d₁₀* (c = 1 × 10^{-3} M, 600 µL) and transferred into an NMR tube. Irradiation was conducted in situ via a fiber optic cable inserted into the NMR tube with a 505 nm LED at -105 °C. In this specific experimental conditions the NMR probe was cooled with liquid nitrogen: the error in the temperature detection is ±10 °C. Moreover, to acquire more points and achieve a better signal-to-noise ratio, the t1 relaxation was cut before its completion (with an additional ±10% error on the integration of the kinetic data). For the above, the kinetic results obtained are only qualitative. These considerations do not hold for the analysis of the distribution of the species at the photostationary state.

Upon irradiation, a new set of aromatic peaks appears and more interestingly, the acetyl CH_3 group shifts ~ 1.2 ppm upfield. This observation is in line with our theoretical calculations of the geometry of *Z*-**PIO**, the most stable conformer of which presents an interaction between the phenyl ring and the CH_3 group itself, with an orientation that leads to increased shielding in the NMR signal (for a visualization of the structure, see Figure 2).

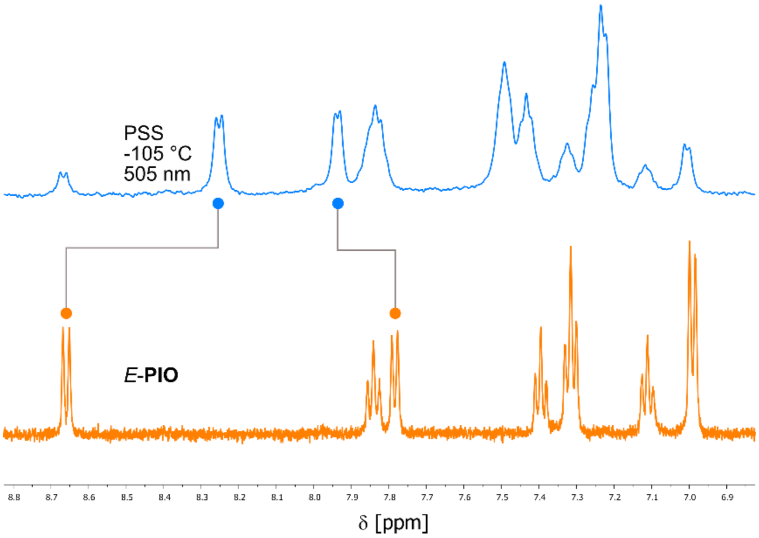

Figure S52. Change in the aromatic region of the NMR spectrum of *E*-**PIO** in Et_2O-d_{10} (1 × 10^{-3} M) upon irradiation with a 505 nm LED at -105 °C.

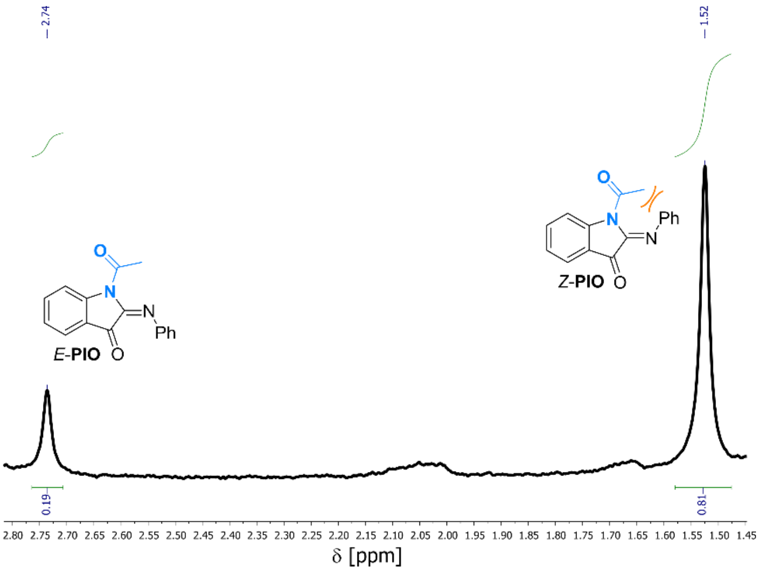

Figure S53. Acetyl region of the NMR spectrum of the photostationary state reached after irradiation of *E*-**PIO** in Et_2O-d_{10} (1 × 10^{-3} M) with a 505 nm LED at -105 °C. The shielding of the signal of the acetylic CH_3 due to the interaction with the phenyl group can be observed. The photostationary distribution at the PSS can be inferred from this measurement as 8:2 *Z*-**PIO**:*E*-**PIO**.

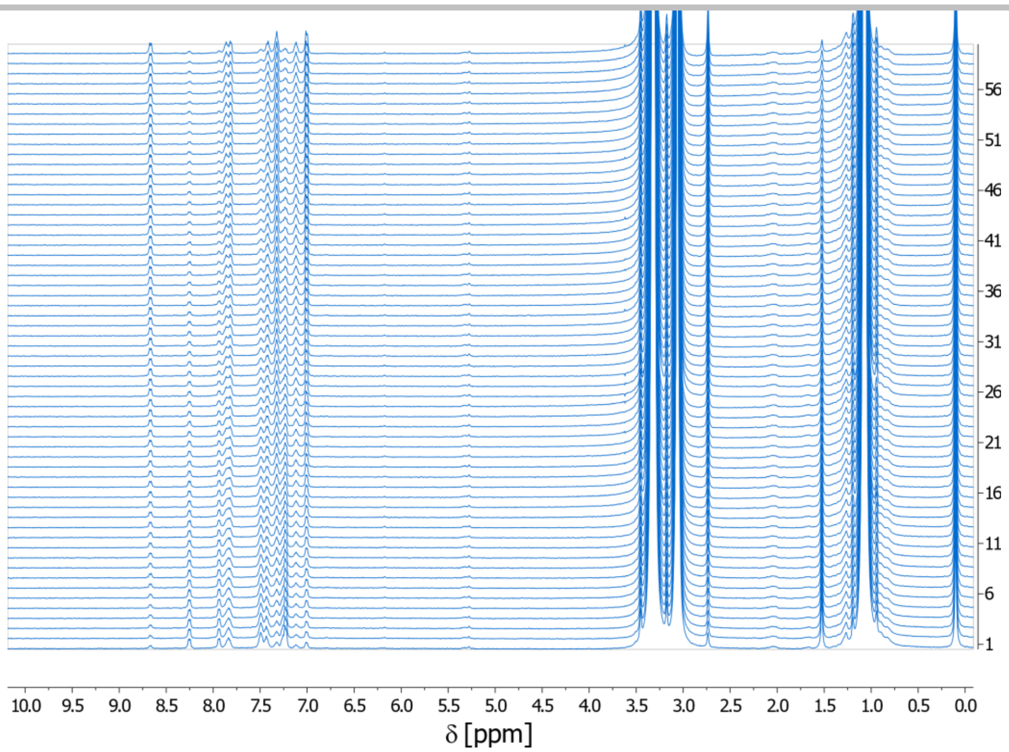

Figure S54. From bottom to top: thermal recovery of the signals of the NMR spectrum, starting from the photostationary state reached after irradiation of *E*-**PIO** in Et_2O-d_{10} (1 × 10^{-3} M) with a 505 nm LED at -105 °C.

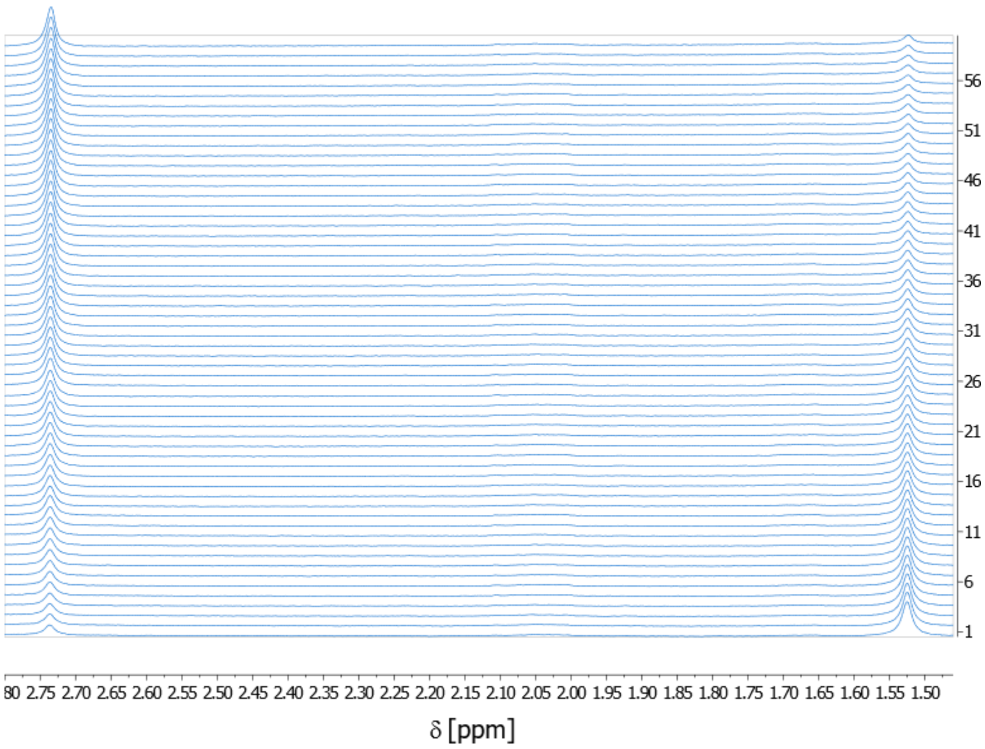

Figure S55. From bottom to top: thermal recovery of the signals in the acetyl region of the NMR spectrum, starting from the photostationary state reached after irradiation of *E*-**PIO** in Et_2O-d_{10} (1 × 10^{-3} M) with a 505 nm LED at -105 °C.

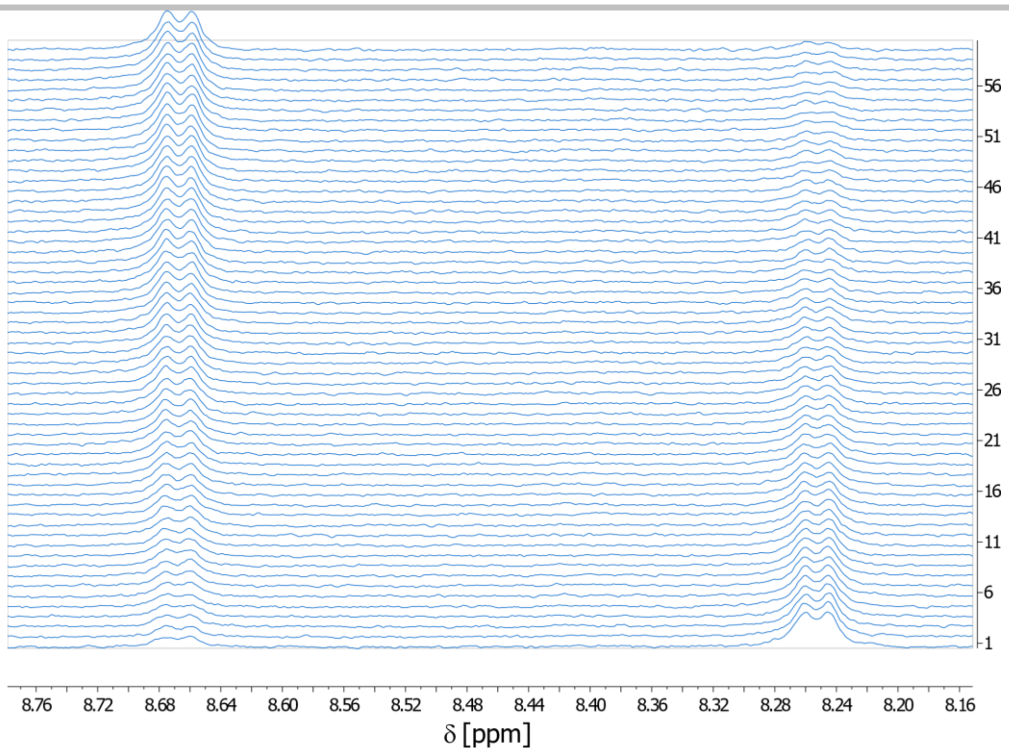

Figure S56. From bottom to top: thermal recovery of the signals in the aromatic region of the NMR spectrum, starting from the photostationary state reached after irradiation of *E*-**PIO** in Et_2O-*d*$_{10}$ (1 × 10^{-3} M) with a 505 nm LED at -105 °C.

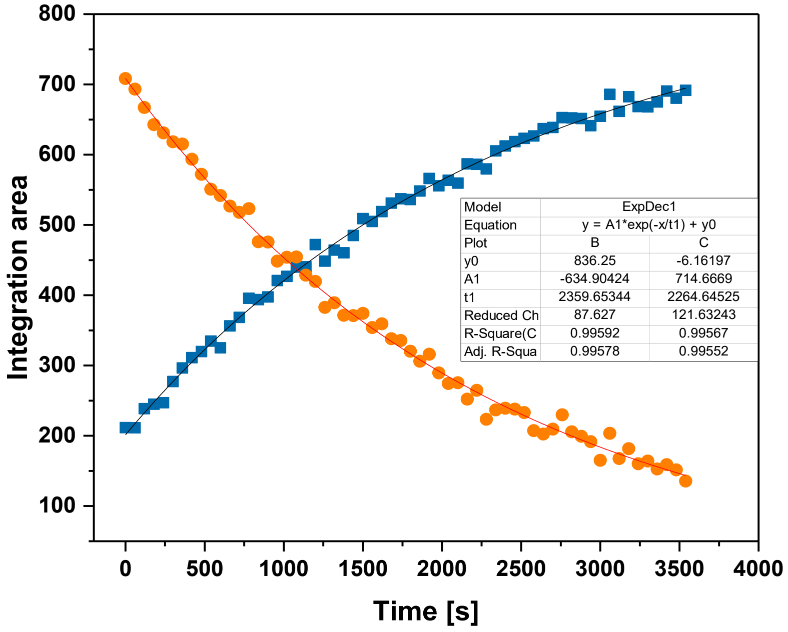

Figure S57. Kinetic of the thermal recovery of the doublet signals in the aromatic region of the NMR spectrum (blue from the signal at 8.67 ppm for *E*-**PIO**, orange from the signal at 8.25 ppm for *Z*-**PIO**), starting from the photostationary state reached after irradiation of *E*-**PIO** in Et_2O-*d*$_{10}$ (1 × 10^{-3} M) with a 505 nm LED at -105 °C. The lifetimes that can be inferred from this measurement differ from the one measured using low-temperature-UV-Vis (2300 s vs 290 s, vide infra). We attribute the cause of this discrepancy to the less accurate reading of the temperature for the NMR measurements, that suffers from a larger error than the UV-Vis itself (±10 °C at -105 °C vs ±1 °C of the UV-Vis). Indeed, a five degree difference recorded around -105 °C can cause the lifetime to increase threefold (see Figure S60). After these considerations and the comparison with the UV-Vis and ns TA data we can confidently state that, within the margin of temperature uncertainty, the same process, viz. the formation of *Z*-**PIO** and its thermal relaxation to the stable *E* form, was observed.

Activation parameters

A sample of *E*-**PIO** in anhydrous Et_2O (c = 3 × 10^{-4} M) was prepared and degassed by bubbling N_2 through the solution. The sample was irradiated at six temperatures (–110, –105, –100, –95, –90 and –85 °C; the error in the temperature measurement is ±1 °C), using a 505 nm LED, and the thermal relaxation was followed on a UV-Vis spectrophotometer. From the change in absorbance at 443 nm, the rate constants (*k*) at the different temperatures were determined by fitting a 1st order rate law. The values were plotted to determine the thermodynamic parameters of isomerization.

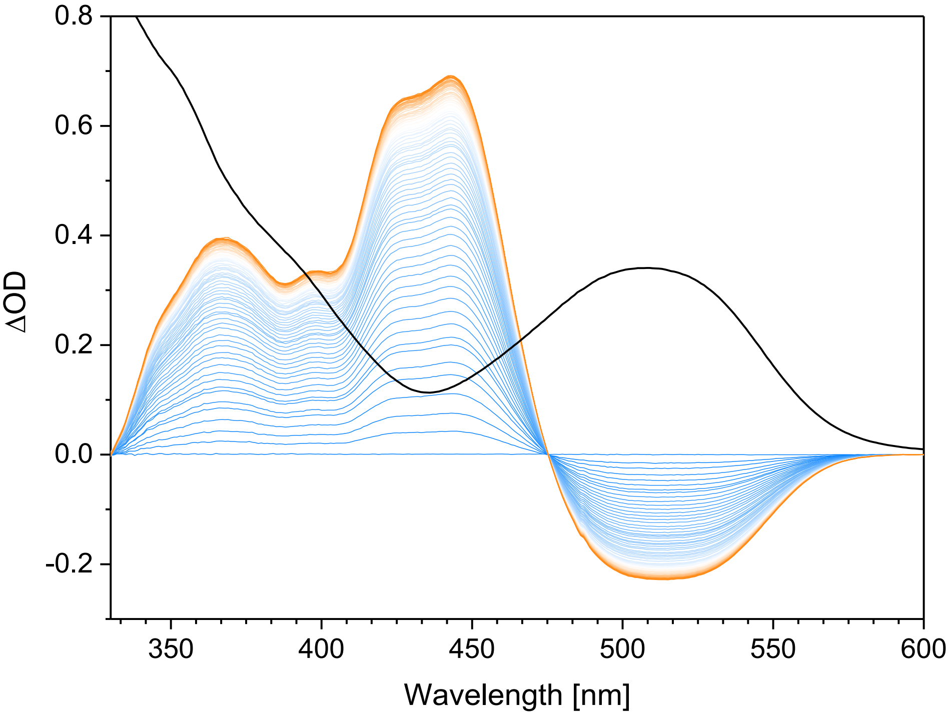

Figure S58. Change in the absorption spectrum of *E*-**PIO** in Et_2O (3 × 10^{-4} M) upon irradiation with a 505 nm LED. PSS was reached after 22 min. In black: original spectrum of *E*-**PIO** before irradiation.

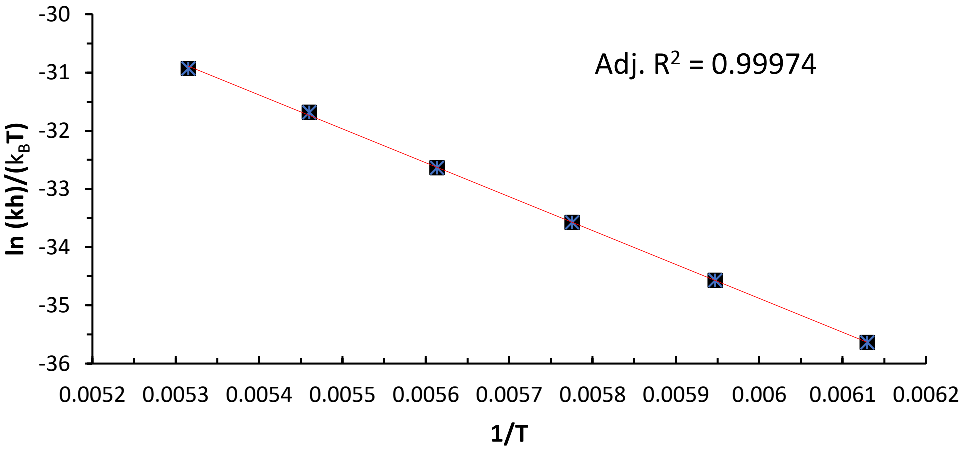

Figure S59. Eyring plot analysis of the thermal isomerization from *Z*-**PIO** to *E*-**PIO** monitored by the decrease in absorption at λ = 443 nm in degassed Et_2O (3 × 10^{-4} M). Parameters at 20 °C: $\Delta G^{\ddagger}$ = 48.3 kJ mol^{-1}, $\Delta H^{\ddagger}$ = 48.4 kJ mol^{-1}, $\Delta S^{\ddagger}$ = 0.54 J mol^{-1} K^{-1}, $t_{1/2}$ = 5.1 × 10^{-5} s, τ = 7.3 × 10^{-5} s, *k* = 1.4 × 10^{4} s^{-1}.

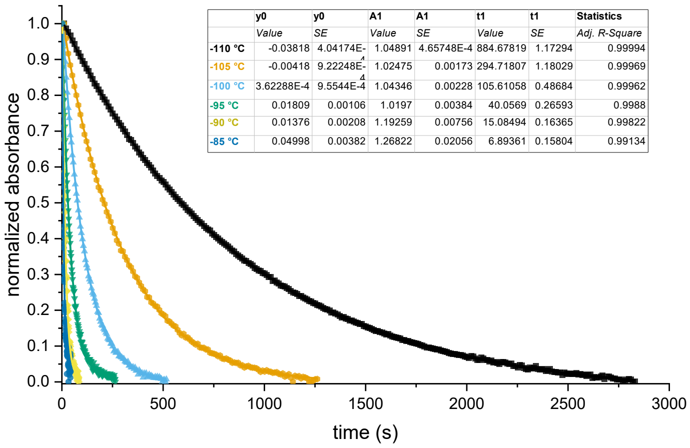

Figure S60. Selected first order decay plots of the band at 443 nm at different temperatures and the respective fits with their relevant parameters obtained by fitting the experimental data to the equation y = A1 $e^{(-x/t1)}$ + y0. The changes in absorbance were normalized for comparison.

Z-to-E isomerisation by irradiation with 455 and 420 nm light

To demonstrate the ability to obtain *E*-**PIO** by irradiation of *Z*-**PIO** at a different wavelength instead of thermal relaxation, a sample of the switch was first irradiated with a 505 nm LED to a ΔOD (443 nm) of ~ 0.35. Subsequently, the isomerization from *E*-**PIO** to *Z*-**PIO** was followed in the case of 1) no irradiation, 2) irradiation with a 455 nm LED, and 3) irradiation with a 420 nm LED. Upon irradiation with a 455 or 420 nm LED the original spectrum was not obtained, but a new PSS was reached following faster kinetics than observed under thermal relaxation conditions.

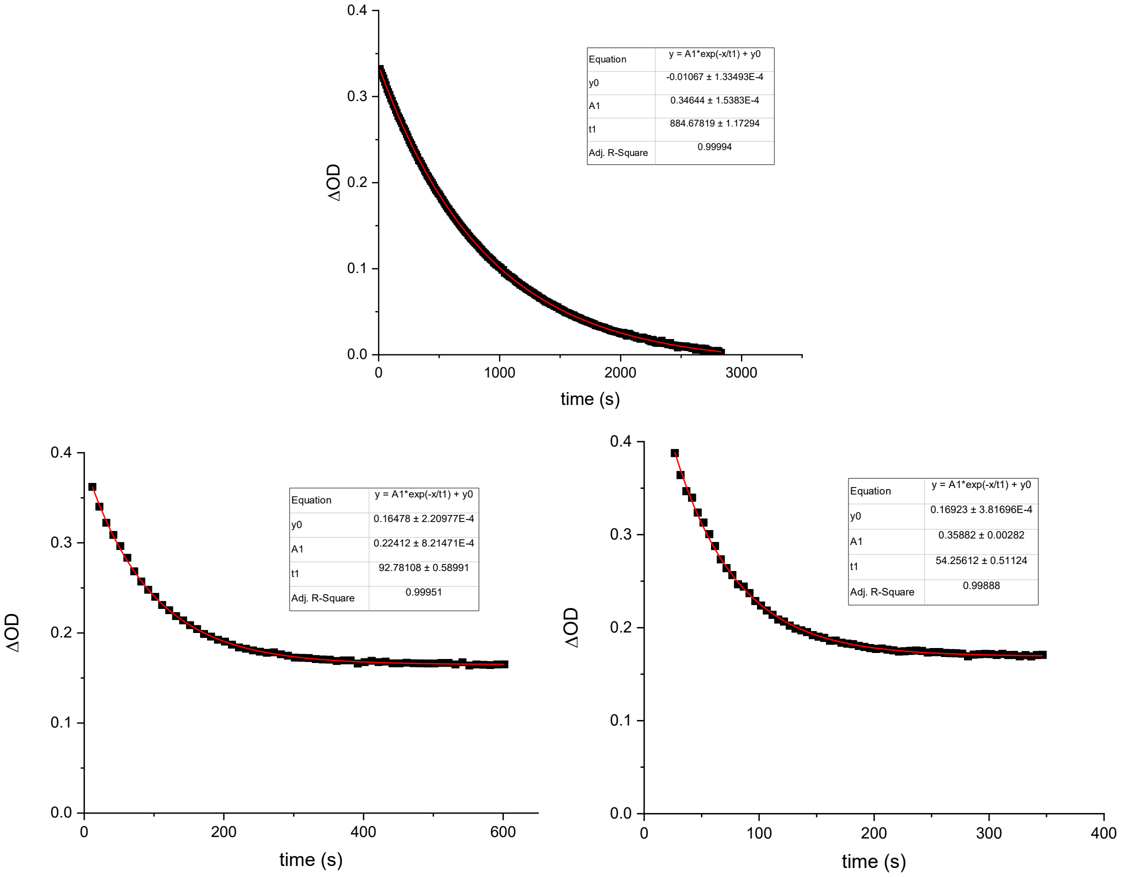

Figure S61. Isomerization of *Z*-**PIO** to *E*-**PIO** at –110 °C after irradiation of *E*-**PIO** with a 505 nm LED. Top: Isomerization in the dark (τ = 885 s). Bottom left: Isomerization upon irradiation with a 455 nm LED (τ = 93 s). Bottom right: Isomerization upon irradiation with a 420 nm LED (τ = 54 s).

Computational analysis

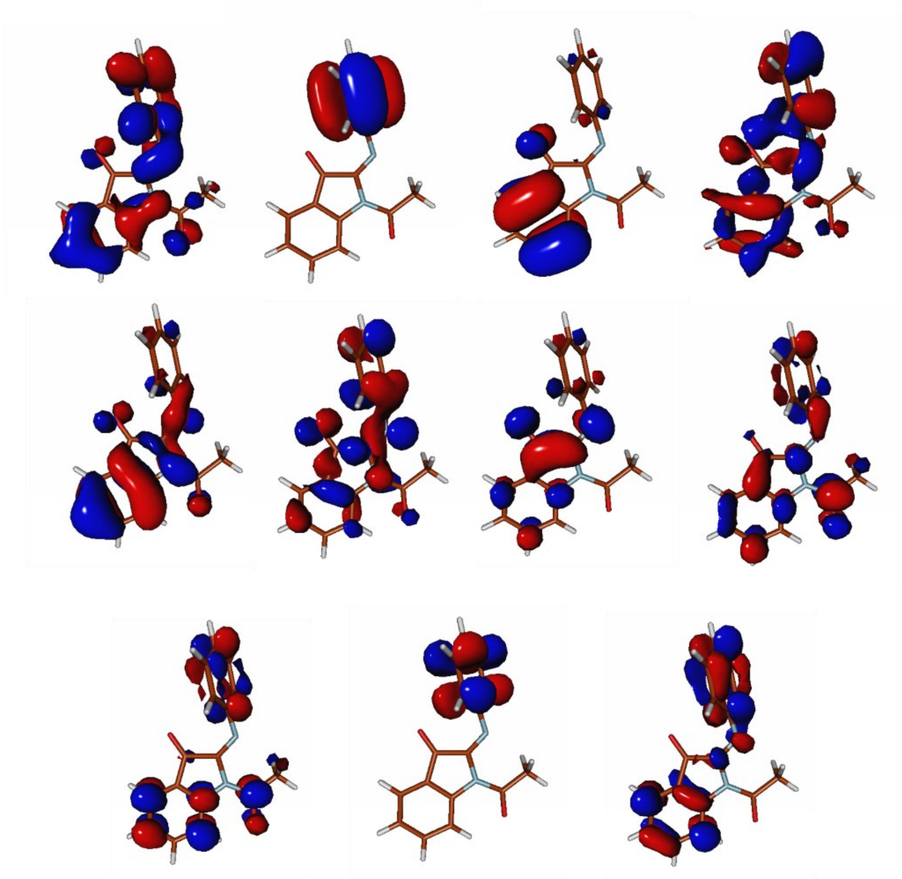

Figure S62. Active space orbitals employed in the OM2/MRCI calculations.

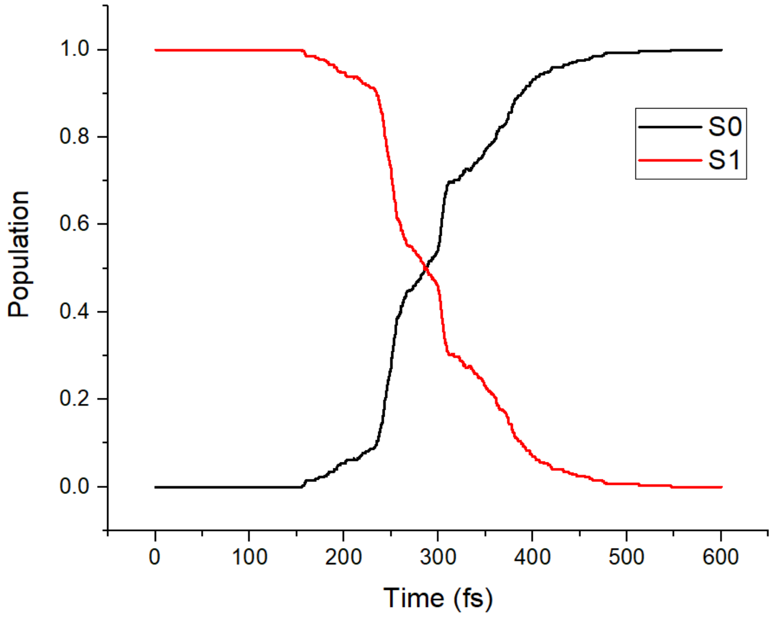

Figure S63. Averaged S_0 and S_1 states population as a function of time.

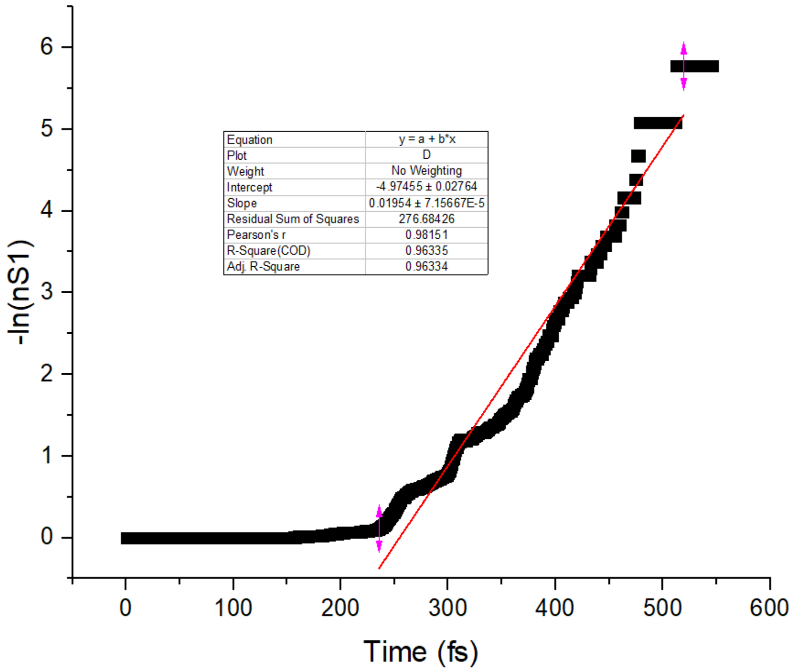

Figure S64. Natural logarithm -ln(n_{S1}) of the S_1 state population as a function of time. The intercept of the linear regression provides an estimate of the excited state lifetime. It is interesting to notice that on top of the decay an oscillatory component is present as well. Despite this conclusion, only one well-defined lifetime can be inferred by the simulation.

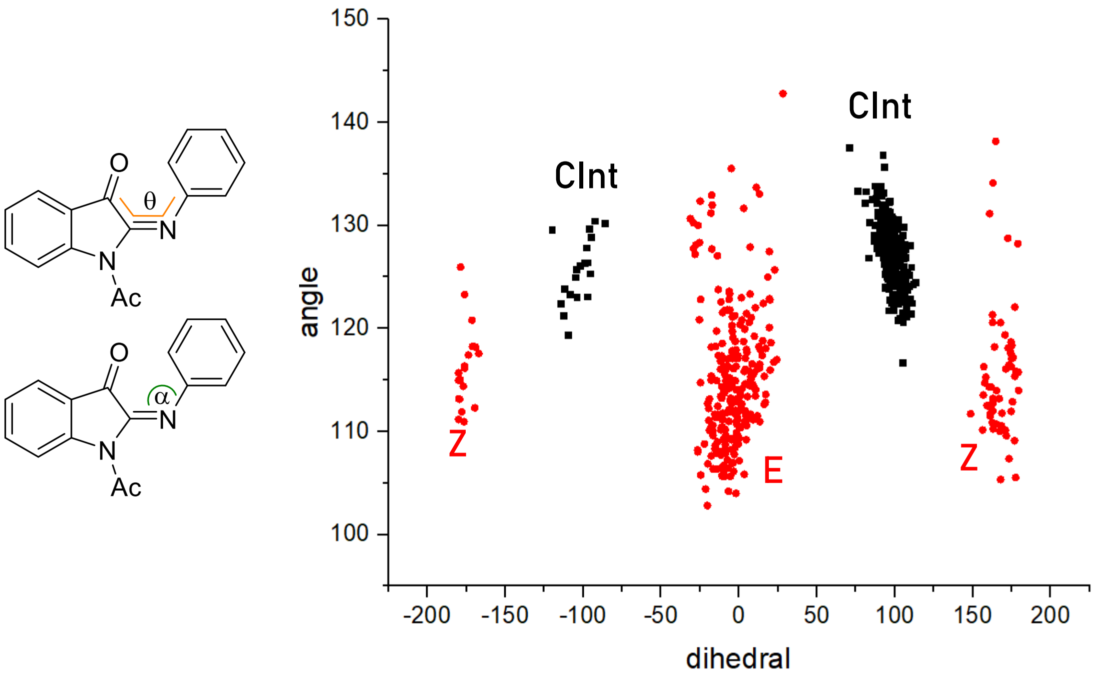

Figure S65. Values of selected dihedral and angle values defining the geometries of the hopping points (in black) and evolution of each trajectory at the time step of 600 fs (in red, ones with a dihedral angle of ca. 0° are geometries corresponding to *E*-**PIO**, ones at ±180° are *Z*-**PIO**). The hopping points (CInt) are mainly distributed around a region defining the so-called "rotation" movement in the excited state with a 90° dihedral angle formally breaking the C=N bond. The estimated quantum yield for the *EZ* isomerization (24%) is obtained by dividing the total number of trajectories that populated the *Z*-form by the total number of trajectories.

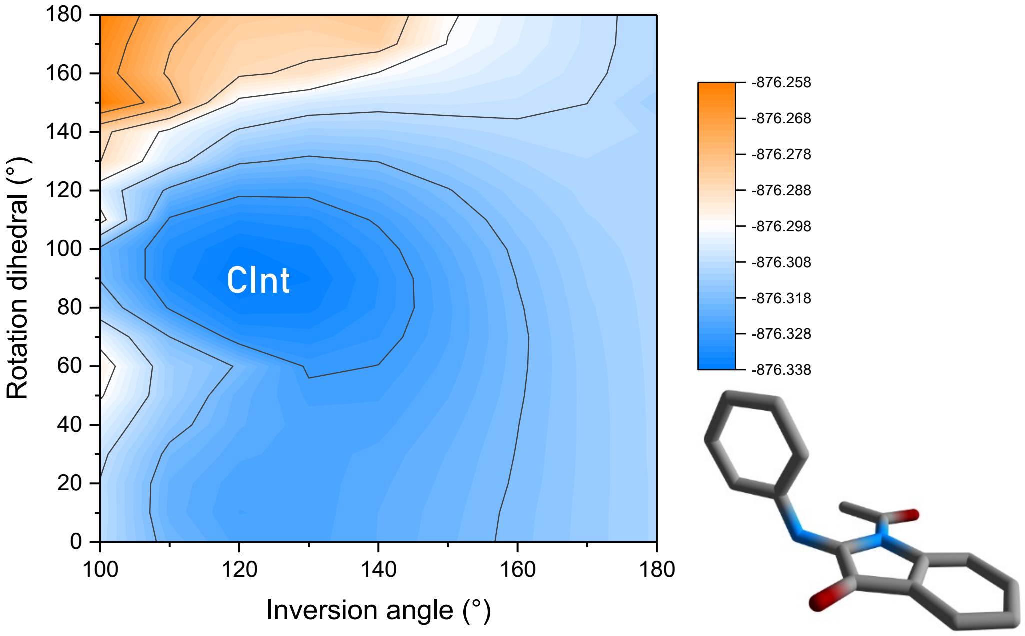

Figure S66. Heatmap of the energy (in Hartrees) of S_1 obtained at the SF-BH&HLYP/cc-PVDZ//TDA-ωB97X-D/MIDI! level of theory (energy diminishes from orange to blue). The scan confirms the presence of an energetic well at ca. 90° of the rotation dihedral (CInt), where the NAMD simulations predict the $S_1 \rightarrow S_0$ hoppings to occur. The excited state around the minimum has a diradical character (e.g S^2=1.09 for angle=120° dihedral=90°), while the ground state is a closed-shell (e.g S^2=0.27 for angle=120° dihedral=90°). For this reason, a monodeterminantal treatment such as DFT can be used to approximate the excited-state geometries even near the crossing point region. Bottom right: the geometry of the minimal energy point at 90° dihedral and 120° inversion angle.

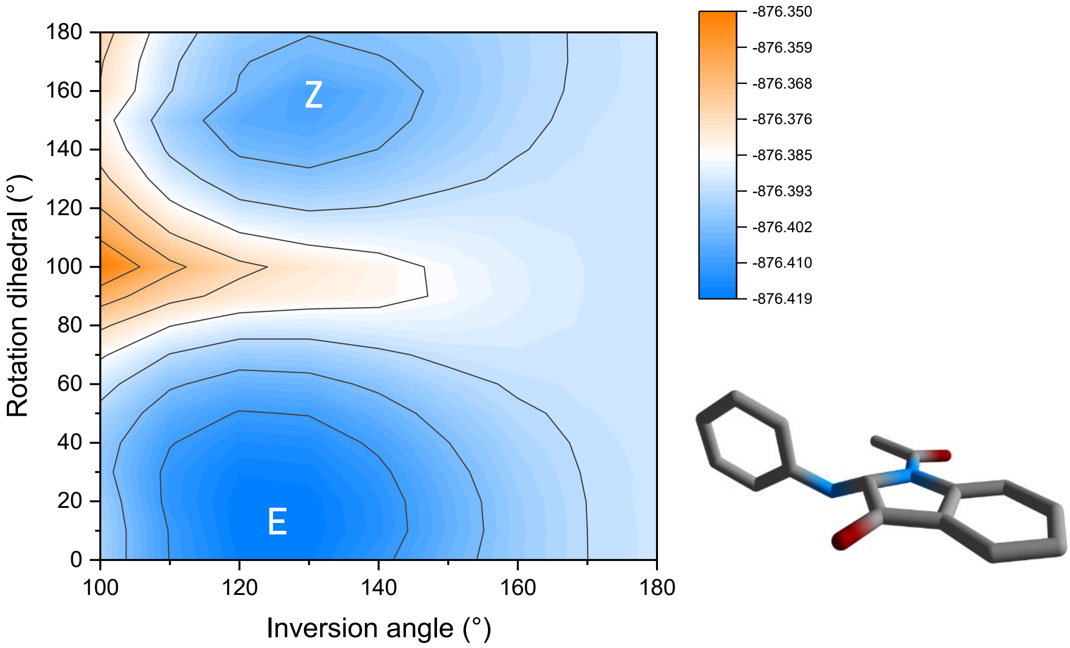

Figure S67. Heatmap of the energy (in Hartrees) of S_0 obtained at the SF-BH&HLYP/cc-PVDZ//GFN2-xTB level of theory (energy diminishes from orange to blue). The scan confirms that the minimum energy path that connects *E* and *Z*-**PIO** passes through a region where the inversion dihedral is around 180°, disfavoring the rotation mechanism in the ground state. All the geometries considered are closed-shell (i.e. $S^2 < 0.5$). The carbon of the CN is pyramidalized in the ground state at geometries with dihedral angles around 90° (see figure in the bottom right corner). Conversely this pyramidalization is lost in the excited state (see Figure S66), confirming the transition from a closed-shell (pyramidalized) to an open-shell (diradical, non-pyramidalized) state.

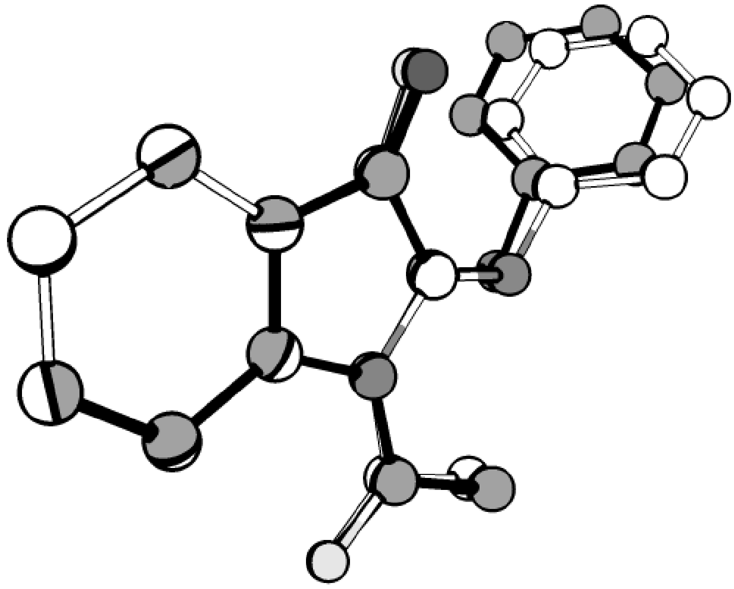

Figure S68. Comparison of the geometries obtained from the X-Ray of *E*-**PIO** (grey) and the corresponding optimized geometries at the M06-2X/def2-SVP level (white). The RMSD is 0.198 (H not considered). The main differences correspond to angle bends, possibly due to the packing in the solid state.

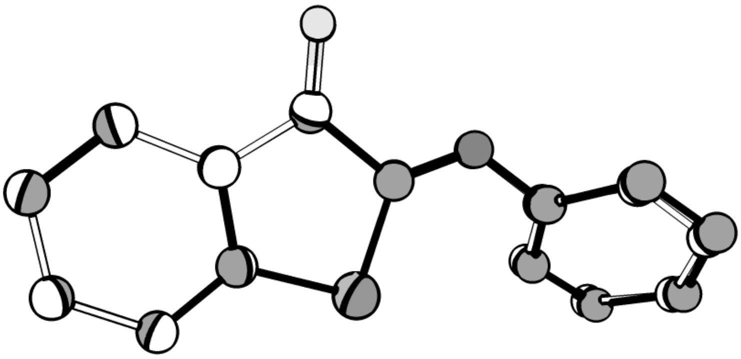

Figure S69. Comparison of the geometries obtained from the X-Ray of *Z*-**ITI** (grey) and the corresponding optimized geometries at the M06-2X/def2-SVP level (white). The RMSD is 0.053 (H not considered).

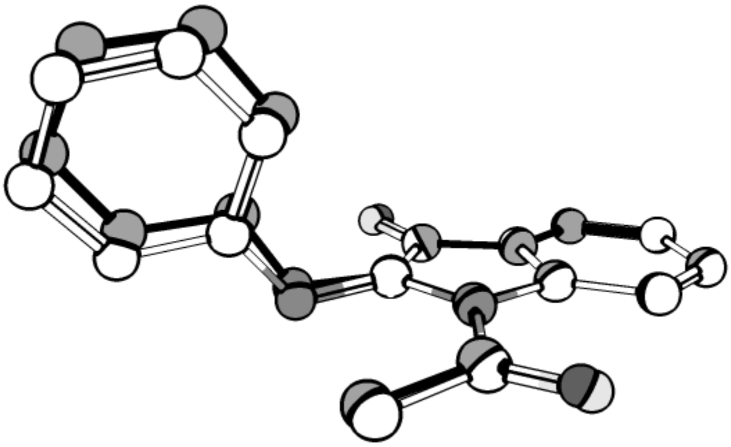

Figure S70. Comparison of the geometries of the CInt of *E*-**PIO** obtained at the SF-BH&HLYP/cc-pVDZ (grey) and the corresponding one optimized at the OM2/MRCI level (white). The RMSD is 0.135. The main difference corresponds to the pyramidalization degree of the C=N bond, slightly more pronounced at the OM2/MRCI level.

Table S16. Energies (electronic, enthalpy and Gibbs Free) and energy differences of the stationary points found at the M06-2X/def2-SVP level.

	E	H	G	ΔE‡	ΔH‡	ΔG‡	ΔE_{E-Z}	ΔH_{E-Z}	ΔG_{E-Z}	δ⁻[d]	δδ⁻[d][e]
		[Hartree]			[kj/mol][a]			[kj/mol][b]			
E-**ITI**	-1066.1598	-1065.9512	-1066.0067								
Z-**ITI**	-1066.1653	-1065.9564	-1066.0115								
TS-**ITI**	-1066.1406	-1065.9332	-1065.9889	50.6	47.3	46.6	14.3	13.5	12.6		
E-***N*-H**	-723.4083	-723.1841	-723.2383								
Z-***N*-H**	-723.4088	-723.1845	-723.2385								
Z-***N*-H**-bis[c]	-723.4079	-723.1845	-723.2362								
TS-***N*-H**	-723.3841	-723.1615	-723.2153	64.8	60.4	60.9	1.3	0.9	0.5		
E-***N*-Me**	-762.6709	-762.4170	-762.4746								
Z-***N*-Me**	-762.6648	-762.4108	-762.4678								
TS-***N*-Me**	-762.6468	-762.3945	-762.4522	47.1	42.8	41.0	-16.0	-16.4	-17.8		
E-**PIO**	-875.8886	-875.6230	-875.6854							-0.48631	
Z-**PIO**	-875.8809	-875.6152	-875.6752							-0.45491	
Z-**PIO**-bis[c]	-875.8729	-875.6075	-875.6689							-0.42479	
TS-**PIO**	-875.8676	-875.6035	-875.6642	34.8	30.9	28.8	-20.3	-20.5	-26.7	-0.45646	-0.00155
E-**PIO-Br**	-3449.1787	-3448.9219	-3448.9887							-0.49061	
Z-**PIO-Br**	-3449.1702	-3448.9134	-3448.9782							-0.45901	
TS-**PIO-Br**	-3449.1574	-3448.9020	-3448.9679	33.7	30.0	27.0	-22.2	-22.3	-27.7	-0.45997	-0.00096
E-**PIO-CN**	-968.0339	-967.7677	-967.8352							-0.48696	
Z-**PIO-CN**	-968.0242	-967.7580	-967.8227							-0.46394	
TS-**PIO-CN**	-968.0140	-967.7492	-967.8138	26.7	23.3	23.4	-25.5	-25.6	-32.7	-0.45705	0.00689
E-**PIO-COOMe**	-1103.5147	-1103.2010	-1103.2748							-0.48279	
Z-**PIO-COOMe**	-1103.5061	-1103.1923	-1103.2636							-0.45821	
TS-**PIO-COOMe**	-1103.4951	-1103.1828	-1103.2539	28.7	25.1	25.3	-22.7	-22.8	-29.4	-0.45337	0.00484
E-**PIO-Me**	-915.1551	-914.8603	-914.9279							-0.48713	
Z-**PIO-Me**	-915.1476	-914.8527	-914.9178							-0.45319	
TS-**PIO-Me**	-915.1334	-914.8401	-914.9064	37.1	33.0	29.8	-19.8	-20.1	-26.5	-0.45682	-0.00363
E-**PIO-NO$_2$**	-1080.1624	-1079.8910	-1079.9605							-0.48705	
Z-**PIO-NO$_2$**	-1080.1522	-1079.8808	-1079.9478							-0.46488	
TS-**PIO-NO$_2$**	-1080.1429	-1079.8728	-1079.9394	24.4	21.1	22.1	-26.8	-26.8	-33.6	-0.45571	0.00917
E-**PIO-OMe**	-990.2801	-989.9790	-990.0464							-0.48873	
Z-**PIO-OMe**	-990.2724	-989.9712	-990.0371							-0.45284	
TS-**PIO-OMe**	-990.2567	-989.9570	-990.0229	41.2	37.4	37.4	-20.4	-20.3	-24.3	-0.47146	-0.01862
E-**iBu-PIO**	-954.4157	-954.0905	-954.1589							-0.48740	
Z- **iBu-PIO**	-954.4102	-954.0850	-954.1509							-0.45807	
TS-**iBu-PIO**	-954.3953	-954.0714	-954.1375	39.1	35.6	35.2	-14.5	-14.5	-20.8	-0.45918	-0.00111

[a] Energy differences calculated between the metastable state and the transition state.

[b] Energy differences calculated between the E-form and the Z-form. Negative values → E more stable

[c] Less stable conformer. Energy differences are always calculated using the most stable conformer.

[d] Natural charge at the imine nitrogen obtained from Natural Population Analysis.

[e] Difference in Natural charges at the imine nitrogen (TS – Z-form).

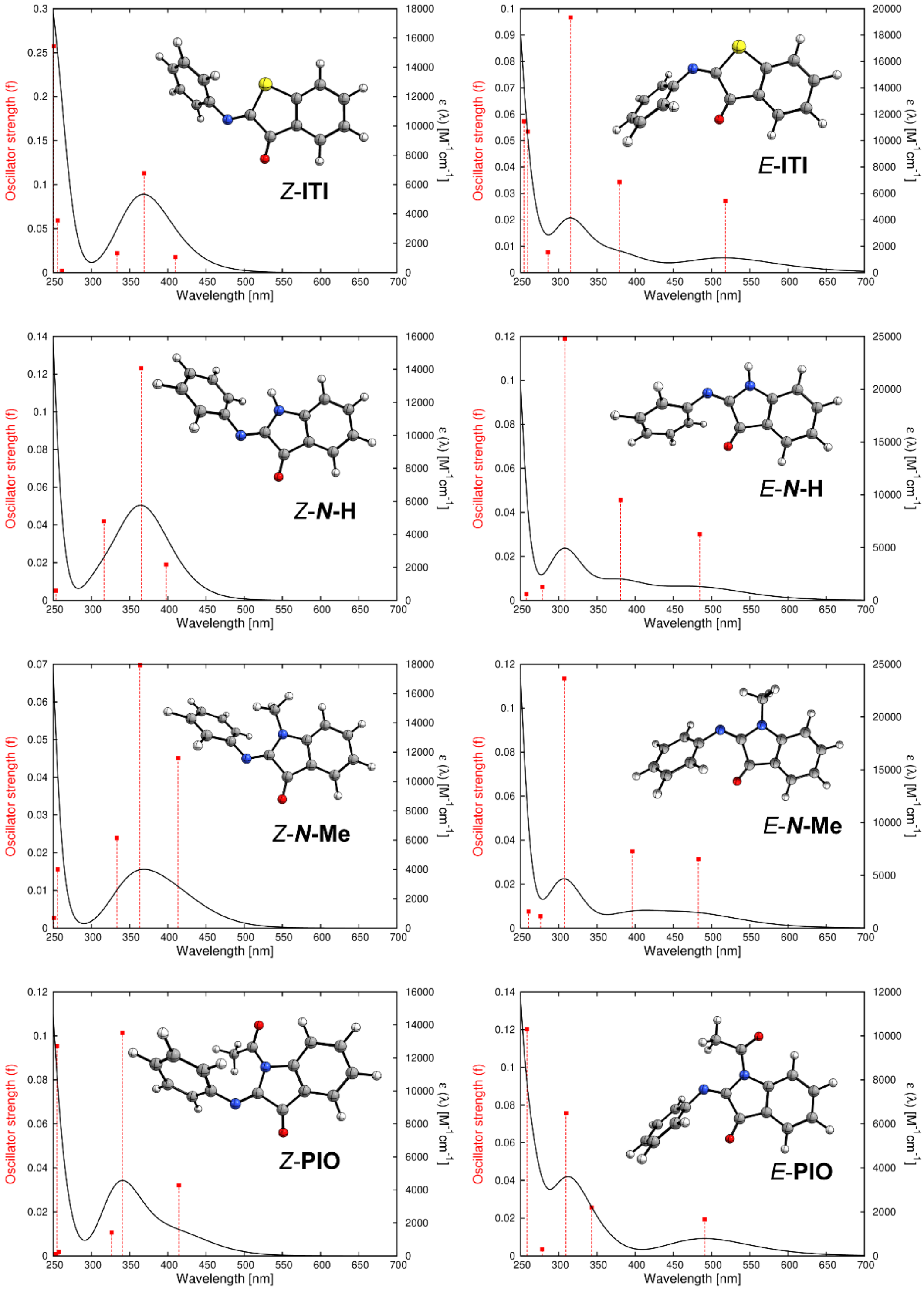

Figure S71. Computed UV-Vis spectra at the M06-2X/6-311+G(2d,p) level of theory for the neutral switches investigated in this work.

References

[1] C. Feldmeier, H. Bartling, E. Riedle, R. M. Gschwind, *J. Magn. Reson.* **2013**, *232*, 39–44.

[2] J. J. Snellenburg, S. Laptenok, R. Seger, K. M. Mullen, I. H. M. van Stokkum, *J. Stat. Softw.* **2012**, *49*, 1–22.

[3] *Bruker, APEX3 (V2019.1-0), SAINT (Version 8.40A) SADABS (Version 2016/1). Bruker AXS Inc., Madison, Wisconsin, USA.* **n.d.**

[4] L. Krause, R. Herbst-Irmer, G. M. Sheldrick, D. Stalke, *J. Appl. Crystallogr.* **2015**, *48*, 3–10.

[5] G. M. Sheldrick, IUCr, *Acta Crystallogr. Sect. A Found. Adv.* **2015**, *71*, 3–8.

[6] G. M. Sheldrick, *Acta Crystallogr Sect. A* **2008**, *A64*, 112–122.

[7] M. Di Donato, M. Segado Centellas, A. Lapini, M. Lima, F. Avila, F. Santoro, C. Cappelli, R. Righini, *J. Phys. Chem. B* **2014**, *118*, 9613–9630.

[8] C. Bannwarth, E. Caldeweyher, S. Ehlert, A. Hansen, P. Pracht, J. Seibert, S. Spicher, S. Grimme, *WIREs Comput. Mol. Sci.* **2021**, *11*, e1493.

[9] C. Bannwarth, S. Ehlert, S. Grimme, *J. Chem. Theory Comput.* **2019**, *15*, 1652–1671.

[10] F. Weigend, R. Ahlrichs, *Phys. Chem. Chem. Phys.* **2005**, *7*, 3297–3305.

[11] Y. Zhao, D. G. Truhlar, *Theor. Chem. Acc.* **2008**, *120*, 215–241.

[12] M. W. H. Hoorens, M. Medved', A. D. Laurent, M. Di Donato, S. Fanetti, L. Slappendel, M. Hilbers, B. L. Feringa, W. Jan Buma, W. Szymanski, *Nat. Commun.* **2019**, *10*, 2390.

[13] M. J. Frisch, G. W. Trucks, H. B. Schlegel, G. E. Scuseria, M. A. Robb, J. R. Cheeseman, G. Scalmani, V. Barone, G. A. Petersson, H. Nakatsuji, X. Li, M. Caricato, A. V. Marenich, J. Bloino, B. G. Janesko, R. Gomperts, B. Mennucci, H. P. Hratchian, J. V. Ortiz, A. F. Izmaylov, J. L. Sonnenberg, D. Williams-Young, F. Ding, F. Lipparini, F. Egidi, J. Goings, B. Peng, A. Petrone, T. Henderson, D. Ranasinghe, V. G. Zakrzewski, J. Gao, N. Rega, G. Zheng, W. Liang, M. Hada, M. Ehara, K. Toyota, R. Fukuda, J. Hasegawa, M. Ishida, T. Nakajima, Y. Honda, O. Kitao, H. Nakai, T. Vreven, K. Throssell, J. Montgomery, J. A., J. E. Peralta, F. Ogliaro, M. J. Bearpark, J. J. Heyd, E. N. Brothers, K. N. Kudin, V. N. Staroverov, T. A. Keith, R. Kobayashi, J. Normand, K. Raghavachari, A. P. Rendell, J. C. Burant, S. S. Iyengar, J. Tomasi, M. Cossi, J. M. Millam, M. Klene, C. Adamo, R. Cammi, J. W. Ochterski, R. L. Martin, K. Morokuma, O. Farkas, J. B. Foresman, D. J. Fox, *Gaussian 16, Rev. B.01* **2016**, Gaussian, Inc., Wallingford CT.

[14] W. Weber, W. Thiel, *Theor. Chem. Acc.* **2000**, *103*, 495–506.

[15] W. Thiel, *Wiley Interdiscip. Rev. Comput. Mol. Sci.* **2014**, *4*, 145–157.

[16] W. Thiel, *MNDO Ver. 7.0* **2017**.

[17] T. W. Keal, A. Koslowski, W. Thiel, *Theor. Chem. Acc.* **2007**, *118*, 837–844.

[18] M. R. Manaa, D. R. Yarkony, *J. Chem. Phys.* **1993**, *99*, 5251–5256.

[19] E. Fabiano, T. W. Keal, W. Thiel, *Chem. Phys.* **2008**, *349*, 334–347.

[20] G. Granucci, M. Persico, A. Zoccante, *J. Chem. Phys.* **2010**, *133*, 134111.

[21] N. Minezawa, M. S. Gordon, *J. Phys. Chem. A* **2011**, *115*, 7901–7911.

[22] G. M. J. Barca, C. Bertoni, L. Carrington, D. Datta, N. De Silva, J. E. Deustua, D. G. Fedorov, J. R. Gour, A. O. Gunina, E. Guidez, T. Harville, S. Irle, J. Ivanic, K. Kowalski, S. S. Leang, H. Li, W. Li, J. J. Lutz, I. Magoulas, J. Mato, V. Mironov, H. Nakata, B. Q. Pham, P. Piecuch, D. Poole, S. R. Pruitt, A. P. Rendell, L. B. Roskop, K. Ruedenberg, T. Sattasathuchana, M. W. Schmidt, J. Shen, L. Slipchenko, M. Sosonkina, V. Sundriyal, A. Tiwari, J. L. Galvez Vallejo, B. Westheimer, M. Włoch, P. Xu, F. Zahariev, M. S. Gordon, *J. Chem. Phys.* **2020**, *152*, 154102.

[23] R. E. Easton, D. J. Giesen, A. Welch, C. J. Cramer, D. G. Truhlar, *Theor. Chem. Acc.* **1996**, *93*, 281–301.

[24] J. Da Chai, M. Head-Gordon, *Phys. Chem. Chem. Phys.* **2008**, *10*, 6615–6620.

[25] E. D. Glendening, C. R. Landis, F. Weinhold, *J. Comput. Chem.* **2019**, jcc.25873.

[26] G. Schwertz, M. C. Witschel, M. Rottmann, U. Leartsakulpanich, P. Chitnumsub, A. Jaruwat, W. Amornwatcharapong, W. Ittarat, A. Schäfer, R. A. Aponte, N. Trapp, P. Chaiyen, F. Diederich, *ChemMedChem* **2018**, *13*, 931–943.

Author Contributions

SC, NAS and WS conceptualized the project. NAS designed the synthetic route. FLK and NAS synthesized the compounds. RT performed the X-Ray analysis. NAS and SC performed UV-Vis and NMR experiments. CNS performed VT-UV-Vis experiments. MDD and SD performed fs spectroscopy and analyzed the data. MH and WJB performed ns TA experiments, WJB supervised the analysis of the TA experiments. SW performed preliminary DFT calculations at the ground state. SC coordinated the work, performed the quantum chemical calculations, helped with the ns TA experiments and performed data analysis. BLF and WS supervised the work. NAS and SC wrote the manuscript. All authors contributed to and approved the final version of the manuscript.
